# Simultaneous Multi‐Resonant Thermally Activated Delayed Fluorescence and Room Temperature Phosphorescence from Biluminescent Nitrogen‐Containing Indolocarbazoles

**DOI:** 10.1002/advs.202503175

**Published:** 2025-05-14

**Authors:** Oliver S. Lee, Aidan P. McKay, David B. Cordes, Stuart L. Warriner, Malte C. Gather, Eli Zysman‐Colman

**Affiliations:** ^1^ Organic Semiconductor Centre EaStCHEM School of Chemistry University of St Andrews St Andrews KY16 9ST UK; ^2^ Organic Semiconductor Centre SUPA School of Physics and Astronomy University of St Andrews St Andrews KY16 9SS UK; ^3^ School of Chemistry University of Leeds Leeds LS2 9JT UK; ^4^ Humboldt Centre for Nano‐ and Biophotonics Department of Chemistry and Biochemistry University of Cologne Greinstr. 4–6 50939 Köln Germany

**Keywords:** biluminescence, MR‐TADF, RTP

## Abstract

Organic biluminescence, the simultaneous emission from both the singlet and triplet excited state manifolds, is a rare and incompletely understood emission process. However, biluminescent compounds have wide‐reaching applications, such as in sensing, anti‐counterfeiting, and optoelectronics, owing to the complex interplay of excited states having distinct spectral profiles and lifetimes. Herein, the biluminescence of a family of polycyclic aromatic heterocycles known as nitrogen‐containing indolocarbazoles (**NICz**) is described. As 1 wt.% doped films in polymethylmethacrylate (PMMA), these compounds exhibit dual fluorescence/room temperature phosphorescence (RTP) with λ_PL_ in the near‐UV (*≈*375 nm) and green (*≈*500 nm), respectively, and remarkably long phosphorescence lifetimes extending into the multi‐second regime. This RTP is shown to persist even at doping concentrations as low as 0.1 wt.%. Additionally, two of the emitters exhibit multi‐resonant thermally activated delayed fluorescence (MR‐TADF)/RTP biluminescence, which, to the best of knowledge, would be the first examples of such behavior. Finally, insight is provided into the dependence of these competing emission pathways on the temperature and concentration, with supporting wavefunction‐based computations.

## Graphical abstract

An extremely rare type of organic biluminescent emission is reported that consists of delayed emission resulting from both the fluorescence (multi‐resonance thermally activated delayed fluorescence) and phosphorescence spectra at room temperature, and the lifetimes of the two processes exhibit opposing dependence with temperature. The emitters are extensively characterized and elucidation of the underlying emission mechanism, and its dependence on molecular structure, is provided.

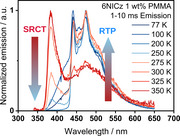

## 1 Introduction

**Figure 1:**
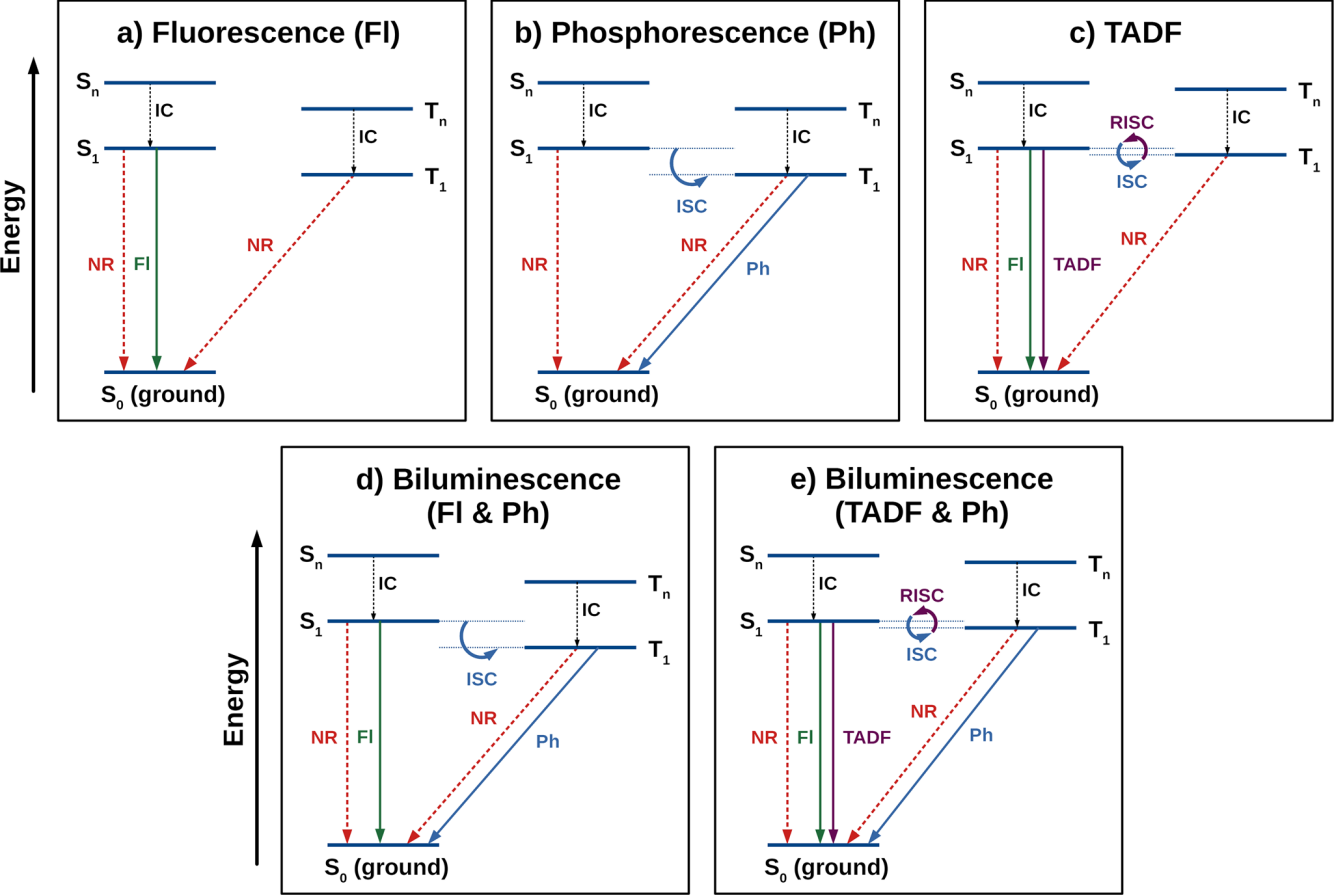
Simplified Jablonski diagrams for a) fluorescence, b) phosphorescence, c) thermally activated delayed fluorescence (TADF), d) fluorescence/phosphorescence biluminescence, and e) TADF/phosphorescence biluminescence. IC: internal conversion, NR: non‐radiative decay, Fl: fluorescence, Ph: phosphorescence, ISC: intersystem crossing, RISC: reverse‐intersystem crossing.

There are many known mechanisms by which a molecule can emit light, classified on the basis of the excited states that are involved in the emission process (**Figure** **1**). By far the most common for all‐organic molecules is fluorescence, the spontaneous emission of light associated with the relaxation of an excited state of the same multiplicity as the ground state (Figure 1a).^[^ ^1^ ^]^ In most organic emitters, which have a closed‐shell configuration in the ground state, this occurs from a singlet excited state and typically has a very short (nanosecond) emission lifetime. Additionally, because high‐lying excited states rapidly relax down to the lowest excited state of the same multiplicity via internal conversion, fluorescence is generally only observed from the lowest‐lying singlet excited state, S_1_. A second emission mechanism involves radiative decay from an excited state of a different multiplicity to the ground state, typically the lowest‐lying triplet excited state (T_1_), and is termed phosphorescence (Figure 1b).^[^ ^2^ ^]^ Phosphorescence necessitates a spin‐flip, termed intersystem crossing, of the relaxing electron from the triplet excited state, where the electrons of the two singly‐occupied molecular orbitals (SOMOs) have parallel spin, to the singlet ground state where they antiparallel spin. Because spin must be conserved during an electronic transition, phosphorescence is formally forbidden in a molecule with well‐defined spin states, and as such, the rate of phosphorescence in organic molecules, which is inversely proportional to the energy gap between the triplet excited state and the ground state, is several orders of magnitude slower than either fluorescence or nonradiative decay from S_1_ or T_1_ and thus is typically not observed. For phosphorescence to become more likely, it is necessary for the singlet and triplet excited states to mix, which is most commonly achieved through spin‐orbit coupling (SOC). SOC is dependent on the difference of the symmetry of the orbitals involved in the transitions to the singlet and triplet excited states as well as scaling rapidly with the atomic number of the atoms involved in these transitions.^[^ ^1^, ^3^, ^4^ ^]^ It is for these reasons that transition metal complexes containing platinoid metals are so frequently phosphorescent.^[^ ^2^ ^]^ As the energy gap between S_1_ and T_1_ (ΔE_ST_) is significantly smaller than that between S_1_ and S_0_, and large SOC in these compounds readily facilitates the mixing of the excited states of different multiplicity, ISC outcompetes fluorescence, and so only triplet emission is observed. However, because of the relatively large gap between T_1_ and S_0_ and the required spin‐flip, the rate of phosphorescence (*k_Ph_*) is slow compared to the typical rate of fluorescence (*k_Fl_*), with a lifetime that typically ranges from microsecond to seconds.^[^ ^1^ ^]^ Phosphorescence can also occur from organic compounds, where it is known as room temperature phosphorescence (RTP) in the community. As these compounds typically do not contain any heavy atoms, they show much smaller SOC, and so RTP generally has to rely on strongly suppressing non‐radiative decay channels.^[^ ^5^, ^6^, ^7^ ^]^

An alternative triplet harvesting mechanism, which does not rely on heavy metals, is thermally activated delayed fluorescence (TADF).^[^ ^8^, ^9^ ^]^ In this process, the ΔE_ST_ of the emitter is sufficiently small to permit endothermic up‐conversion of triplets to singlets via reverse‐intersystem crossing (RISC). This is possible even with negligible SOC, which also makes phosphorescence a disfavoured radiative decay process.^[^ ^10^ ^]^ Therefore, TADF materials exhibit dual fluorescence with two distinct lifetimes: 1) prompt fluorescence from radiative decay from directly populated singlet excited states, a process that is identical to the fluorescence introduced above and therefore has a typical lifetime of nanoseconds; and 2) delayed fluorescence from the same singlet excited state that is repopulated by ISC/RISC cycling.^[^ ^11^ ^]^ Because this up‐conversion relies on a formally forbidden and thermodynamically uphill spin‐flip process, it is slow, and thus delayed fluorescence typically occurs on a microsecond‐to‐millisecond timescale.^[^ ^1^ ^]^ As RISC is an endothermic process, the rate of TADF is strongly dependent on the temperature, exhibiting faster rates at higher temperatures and slower or absent rates at lower temperatures. This is opposite to the behavior observed for phosphorescent compounds wherein at higher temperatures there is a decrease in the delayed emission contribution owing to increased non‐radiative deactivation of the T_1_ state through molecular vibrations.^[^ ^5^ ^]^ To enable TADF in an emitter it is necessary to adopt a molecular design that minimizes the exchange integral between the frontier orbitals involved in the radiative transition. There are numerous design strategies used in the literature to achieve this, with multi‐resonant thermally activated delayed fluorescence (MR‐TADF) having received particular attention in recent years. This mechanism relies on a much shorter charge‐transfer distance in the S_1_ → S_0_ transition than is found in the more traditional donor‐acceptor based long‐range charger‐transfer TADF compounds, resulting in a significantly narrower emission spectrum in the former compared to the latter.^[^ ^12^ ^]^ Many of the compounds that exhibit MR‐TADF emission are based on a p‐ and/or n‐doped nanographene backbone.^[^ ^13^ ^]^

Biluminescence, or dual emission, is the simultaneous emission from multiple excited states of the same molecules.^[^ ^14^ ^]^ This diverse category of emission processes encompasses many different mechanisms, including S_1_/S_2_ non‐Kasha emission which can be observed in some organic molecules such as porphyrins^[^ ^15^, ^16^ ^]^ and azulenes,^[^ ^17^ ^]^ and S_1_/T_1_ dual fluorescence (or TADF)/phosphorescence that is observed in several Cu(I)‐based organometallic emitters.^[^ ^18^ ^]^ Examples of S_1_/T_1_ biluminescence in all‐organic emitters are rare, and the first report of an organic S_1_/T_1_ biluminescent emitter to be rigorously characterized was only in 2013 by Reineke and co‐workers,^[^ ^19^ ^]^ although the phenomenon itself is older.^[^ ^9^, ^20^, ^21^ ^]^ The mechanism(s) of these processes are poorly understood, but in qualitative terms they necessitate a careful balance of the rate constants of fluorescence, intersystem crossing, phosphorescence, and non‐radiative decay. Additionally, many of the reported examples of S_1_/T_1_ biluminescence exhibit TADF in addition to conventional fluorescence,^[^ ^14^, ^19^, ^22^, ^23^ ^]^ which further necessitates the balancing of the rate of reverse‐intersystem crossing with the other photophysical processes. Full design rules by which this can be reliably achieved have not yet been established, but it can be assumed that S_1_/T_1_ biluminescence requires intermediate values of ΔE_ST_ and SOC, to obtain competitive rates from fluorescence/TADF and RTP while minimizing non‐radiative decay. Biluminescence promises to be a hugely important field because it possesses two distinct spectral bands with distinct lifetimes, oxygen dependence, and, in the case of TADF/RTP biluminescence, contrasting temperature dependences. This enables a diverse range of applications, including the simultaneous sensing of temperature and oxygen,^[^ ^24^ ^]^ data security and counterfeit protection,^[^ ^25^, ^26^ ^]^ and white‐light‐emitting OLEDs.^[^ ^22^ ^]^ As such, there is enormous value in both providing insight into this mechanism and in introducing new molecular scaffolds that exhibit biluminescence. In particular, we note that there are no previous reports of a biluminescent MR‐TADF/RTP emitter.

**Figure 2:**
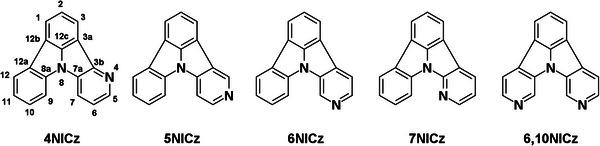
Structures, names, and numbering schemes of the five emitters investigated.

With the initial motivation to develop deep‐blue/near‐UV MR‐TADF emitters, and building on our previous work on diindolocarbazole‐based emitters,^[^ ^27^ ^]^ we chose to investigate the photophysical properties of the nitrogen‐containing indolocarbazole (NICz) family of emitters. This family of compounds was previously described by Kader *et al.*,^[^ ^28^ ^]^ and in solution, these compounds have been shown to be near‐UV fluorescent emitters. However, their photophysical properties in the film state have, to the best of our knowledge, not previously been explored. Here, we evaluated the photophysical properties of four singly substituted NICz molecules, **4NICz**, **5NICz**, **6NICz**, and **7NICZ**, as well as one‐doubly substituted molecule, **6,10NICz** (**Figure** **2**). We find that three of the emitters exhibit dual fluorescence/RTP emission in doped PMMA films, even at very low doping concentrations, and dual MR‐TADF/RTP emission in the emitters **6NICz** and **6,10NICz**, which is likely to be the first example of S_1_/T_1_ biluminescence from a MR‐TADF emitter. The lifetimes associated with both the RTP and TADF are very long, with perceptible afterglow from the phosphorescence extending into the multi‐second regime. By varying doping concentrations and temperature, we can influence the relative contributions from fluorescence, TADF, phosphorescence, and non‐radiative deactivation. Finally, we provide insight into the change in the emissive pathways of the different isomers based on the changing electronics of the pyridine substitution patterns.

## 2 Computations

We began our investigation by first evaluating the family of NICz compounds as potential TADF emitters. Although there is a multitude of conceivable substitution patterns, we chose to focus only on the singly‐substituted emitters (**4NICz**, **5NICz**, **6NICz**, and **7NICz**) and one example of a doubly substituted NICz, **6,10NICz**, as these are by‐far the most synthetically accessible. By screening all five emitters, we aim to provide greater insight into the structure‐property relationship of the molecular family. The excited states of multi‐resonant TADF molecules emitting via a short‐range charge‐transfer (SRCT) mechanism are well‐known to be poorly modeled by time‐dependent density functional theory (TD‐DFT),^[^ ^29^ ^]^ owing to the lack of double excitation character in those methods. As such, we opted instead for the second‐order algebraic diagrammatic construction method (ADC(2)),^[^ ^30^, ^31^, ^32^ ^]^ which we had previously demonstrated to accurately predict the properties of MR‐TADF emitters.^[^ ^29^, ^33^ ^]^ All ADC(2) calculations were performed using the Turbomole package,^[^ ^34^ ^]^ using the resolution of the identity approximation (RI) to reduce the calculation duration,^[^ ^35^ ^]^ spin‐component scaling (SCS) for improved accuracy,^[^ ^36^ ^]^ and the cc‐pVDZ basis set.^[^ ^37^, ^38^, ^39^ ^]^ All computations were managed with the Digichem software package.^[^ ^40^ ^]^ Full computational details are available in the SI (Section S3, Supporting Information).

**Figure 3:**
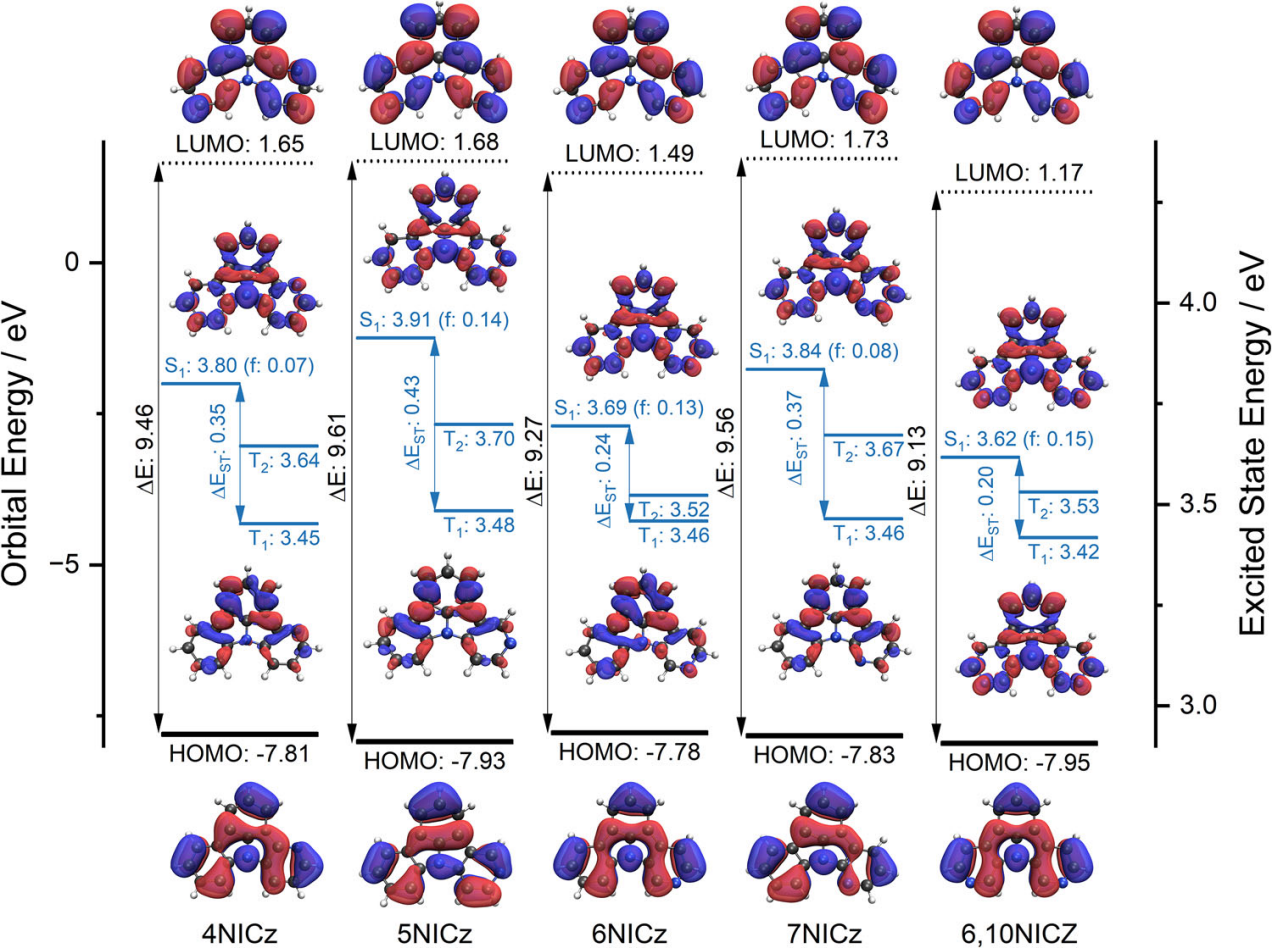
Computed ground state (HOMO and LUMO) and excited state (S_1_,_,_T_1_, and T_2_) properties of **4NICz**, **5NICz**, **6NICz**, **7NICz**, and **6,10NICz**, calculated at the RI‐SCS‐ADC(2)/cc‐pVDZ level of theory in the gas phase. All values are given in eV. For each emitter, the rendered images are, in order from top to bottom: 1) electron density plot of the LUMO; 2) electron difference density plot between the ground state (S_0_) and S_1_; 3) electron difference density plot between S_0_ and T_1_; and electron density plot of the HOMO. All density plots are rendered using an isovalue of 0.02. The red region of the difference density plots corresponds to an increase in electron density in the excited state; the blue to a decrease.

We first optimized the geometry of each emitter (**Figure** **3**), starting from structures drawn in silico. In all cases, the emitters adopt a planar geometry, with a 0.1° or smaller dihedral angle across the central indole nitrogen (**Table** **1**), leading to complete conjugation of the π‐system throughout each emitter. In general, all five molecules have a low‐lying HOMO at ≈‐7.9 eV that is relatively invariant with structure, varying between **─**7.78 eV for **6NICz** and **─**7.95 eV for **6,10NICz**. No trend between HOMO energy and pyridine nitrogen position can be ascertained, with the structurally analogous **6NICz** and **6,10NICz** having the highest and lowest HOMO levels, respectively. By contrast, the LUMOs of these compounds are very high‐lying, with a mean predicted energy of 1.5 eV, and showed greater variation. **6,10NICz** has the most stabilized LUMO (1.17 eV), followed by **6NICz** (1.49 eV), **4NICz** (1.65 eV), **5NICz** (1.68 eV), and **7NICz** (1.73 eV). Here, a structure‐property relationship emerges that we will see repeated throughout this study and that can be empirically derived based on the relative position of the pyridine nitrogen to that of the central indole nitrogen. Namely, structures in which the pyridine N is situated in an *ortho*‐ or *para*‐like position (**7NICz** and **5NICz**, respectively) tend to have higher energies (LUMO, ΔE_HOMO‐LUMO_, S_1,_ and ΔE_ST_) than those in a *meta*‐like position (**4NICz**, **6NICz**, and **6,10NICz**). Among the *meta‐*substituted emitters, **6NICz** and **6,10NICz**, which have nitrogen at the 6‐position, have more stabilized LUMOs than **4NICz**, where the nitrogen is instead at the 4‐position. As the HOMO energies are insensitive to structural variation, this pattern is naturally reproduced in the calculated HOMO‐LUMO gap (ΔE_HOMO‐LUMO_). The ΔE_HOMO‐LUMO_ is very large overall, but the greatest occurs in **5NICz** and **7NICz** (9.61 and 9.56 eV, respectively) and smallest in both **6NICz** and **6,10NICz** (9.27 and 9.13 eV, respectively), while the value for **4NICz** is intermediate at 9.61 eV.

**Table 1:** Calculated ground and excited state properties.

Emitter	HOMO^a)^ / eV	LUMO^a)^ / eV	E_HOMO‐LUMO_^a)^ / eV	S_1_^b)^ / eV	T_1_^b)^ / eV	ΔE_STb)_ / eV	θ^c)^ / °
4NICz	−7.81	1.65	9.46	3.80	3.45	0.35	< 0.1
5NICz	−7.93	1.68	9.61	3.91	3.48	0.43	< 0.1
6NICz	−7.78	1.49	9.27	3.69	3.46	0.24	< 0.1
7NICz	−7.83	1.73	9.56	3.84	3.46	0.37	< 0.1
6,10NICz	−7.95	1.17	9.13	3.62	3.42	0.20	0.1

^a)^ Calculated at the ground state;

^b)^ Vertical excited states, calculated at the ground‐state geometry;

^c).^ The mean dihedral angle between atoms C_7_, C_7a_, N_8_, C_8a_, and C_7a_, N_8_, C_8a_, C_9_. All calculations were performed using the RI‐SCS‐ADC(2)/cc‐pVDZ level of theory, in the gas phase. See Table S2 (Supporting Information) for extended excited‐state calculation results.

In contrast to the variation of the LUMO energies, the electron density plot of the LUMO is identical in all five emitters (Figure 3), having significant contributions from all carbon and nitrogen atoms except C_2_, C_12c_, and the central N_8_ (see Figure 2 for numbering). The HOMO densities, however, form two distinct groups in their distribution. The HOMOs of **6NICz** and **6,10NICz** are symmetrically distributed in a three‐tiered radial pattern around the central N_2_, while the HOMOs of **4NICz**, **5NICz,** and **7NICz** are perturbed away from this symmetry, forming more distinct lobes and overall possessing more nodal planes. Again, the electron density of **4NICz** lies intermediate of the two patterns. The indole nitrogen has a significant density in the HOMO but not the LUMO because of its conjugated lone pair. The peripheral pyridyl nitrogen is noticeably electron‐accepting in **6NICz** and **6,10NICz**, having density only in the LUMO, while in **5NICz** and **7NICz** it has both accepting and donating character, reflected in contributions to both frontier molecular orbitals. In general, there is incomplete separation of the HOMO‐LUMO densities in all five emitters, which results in both a larger S_1_ oscillator strength and ΔE_ST_.

The calculated S_1_ energies are all very high for a conjugated organic compound (Table 1), mirroring both the magnitude and the trend of the large calculated HOMO‐LUMO gaps, with all excited‐state energies lying > 3.1 eV. The lowest S_1_ energy is 3.62 eV in **6,10NICz**, and the highest is 3.91 eV in **5NICz**. The S_0_‐S_1_ oscillator strengths (*f*) do not follow this pattern, however, with values of *≈*0.14 for **5NICz**, **6NICz**, and **6,10NICz** while for **4NICz** and **7NICz** the value is half that at *≈*0.07. The T_1_ energies, on the other hand, vary very little with structure, changing by only 60 meV between **6,10NICz** (3.42 eV) and **5NICz** (3.48 eV). As such, the trend in calculated ΔE_ST_ matches that for the S_1_ energies, with **6,10NICz** and **6NICz** having the smallest ΔE_ST_ at 0.20 and 0.24 eV, respectively, followed by **4NICz** at 0.35 eV, and then **7NICz** and **5NICz** at 0.37 and 0.43 eV, respectively. Notably, both **6NICz** and **6,10NICz** have a predicted ΔE_ST_ that is sufficiently small to show TADF.^[^ ^29^ ^]^ All five emitters additionally have a T_2_ state of different orbital type to S_1_ (Figure S38, Supporting Information) that is energetically intermediate to that of S_1_ and T_1_ (T_2_ = 3.64, 3.70, 3.52, 3.67, and 3.53 for **4NICz**, **5NICz**, **6NICz**, **7NICz**, **6,10NICz** respectively). This has been shown to be beneficial for enhancing RISC as the RISC mechanism may proceed via the intermediate T_2_ state, and when different orbital types are involved in T_2_ and S_1_ then this results in stronger SOC between these two states and thus faster *k*_RISC_, according to El‐Sayed's rule.^[^ ^41^, ^42^ ^]^

By calculating the difference in total electron density between each excited state and the ground state (S_0_), it is possible to visualize the spatial distribution of each excited state (Figure 3). The S_1_ density is identical across all five emitters and demonstrates clear SRCT character, i.e. there is an alternating pattern of increasing and decreasing density on the donor and acceptor atoms; this is the behavior typically observed for MR‐TADF compounds. The T_1_ density distributions show greater variation than the S_1_, despite the consistency in T_1_ energies, and form three distinct groups. The *ortho‐*/*para‐* emitters **5NICz** and **7NICz** possess a largely symmetrical T_1_, with a nodal plane passing through C_2_, C_12c,_ and N_8_, while in the *meta*‐like emitters **4NICz** and **6NICz** this symmetry is distorted, with additional contribution from C_2_ and N_8_. Finally, **6,10NICz** uniquely adopts the same distribution in its T_1_ state as its S_1_ state (Figure S38, Supporting Information).

We have additionally calculated the vibrationally‐resolved emission spectrum from S_1_ for each emitter (Figure S44, Supporting Information). As analytical vibrational frequencies of excited states are not currently available for ADC(2), we have instead calculated these spectra using DFT at the PBE0^[^ ^43^, ^44^, ^45^ ^]^ level using Gaussian 16.^[^ ^46^ ^]^ Although the resulting predicted emission energies are too high by *≈*0.23 eV, the resulting spectral shapes are in excellent agreement with the experimental results. We discuss these calculations alongside the experimental PL spectra in toluene below. Full details of the DFT methodology are available in the SI (Section S3, Supporting Information).

## 3 Synthesis

**4NICz**, **5NICz**, **6NICz**, **7NICz**, and **6,10NICz** were synthesized according to the scheme reported by Kader *et al.*,^[^ ^28^ ^]^ full synthetic details are provided in the supplementary information. The structures of all intermediates and target compounds were unambiguously verified using ^1^H NMR and ^13^C NMR spectroscopy. The target compounds **4NICz, 5NICz, 6NICz, 7NICz,** and **6,10NICz** were additionally characterized by HRMS, melting point determination, and single‐crystal X‐ray diffraction (Figure **S37** **, Supporting Information**). The purity of **4NICz, 5NICz, 6NICz, 7NICz,** and **6,10NICz** was confirmed via HPLC (Figures S11,S16,S21,S26, and S31, Supporting Information).

## 4 Solution‐State Photophysics

**Figure 4:**
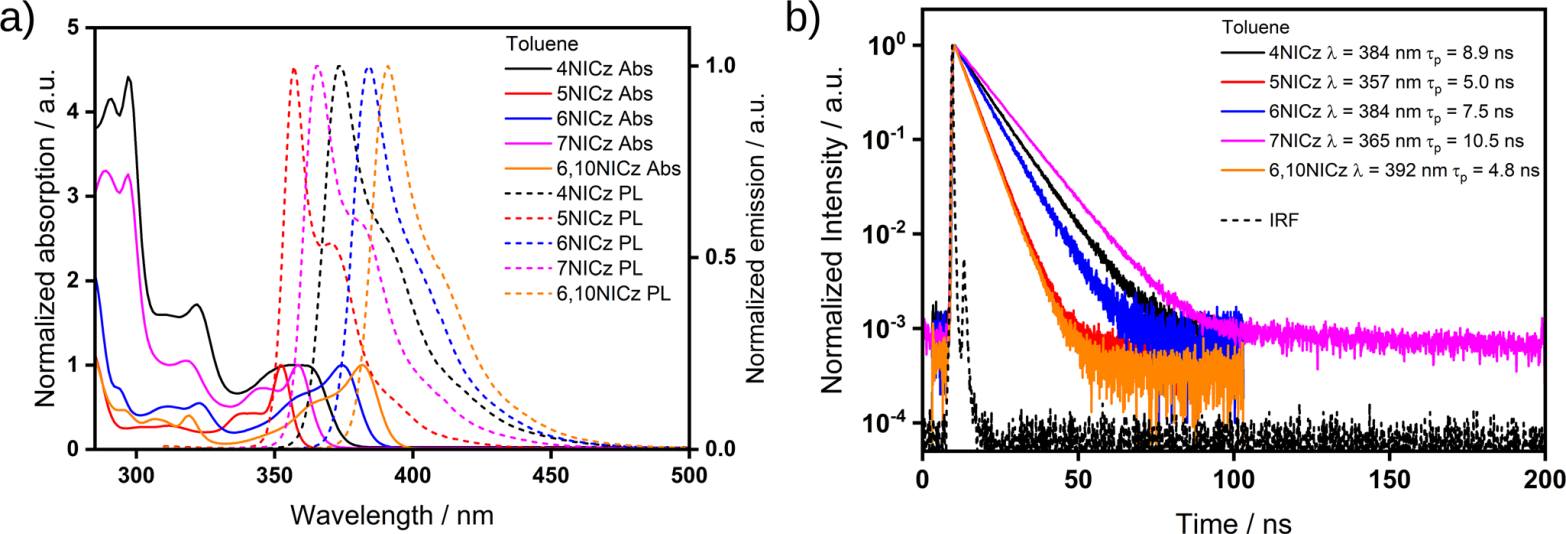
a) The UV–vis absorption and photoluminescence (PL) spectra of the emitters in optically dilute toluene solutions (≈10^−5^ m), Abs: absorption, PL: photoluminescence. The absorbance has been normalized to the band at *≈*350–375 nm. *λ*_exc_ = 297 nm (**4NICz**), 287 nm (**5NICz**), 325 nm (**6NICz**), 297 nm (**7NICz**) and 286 nm (**6,10NICz**). b) Time‐resolved PL decays of the emitters in optically dilute solutions (≈10^−5^ m) recorded by time‐correlated single photon counting (TCSPC), λ_exc_ = 375 nm. λ values in the legend indicate the emission collection wavelength for each compound; τ_p_ values represent the results of a monoexponential fit to the data. IRF: instrument response function.

There are three distinct features in the UV–vis absorption spectra of each emitter in dilute toluene solution (**Figure** **4**a). These are: 1) a low energy band with a peak between 350–400 nm with an additional high‐energy resolved shoulder; 2) a band with a peak ≈320 nm; and 3) a structured high‐energy band with a peak below 300 nm. The high‐energy shoulder of the low‐energy band is blue‐shifted by ≈15 nm relative to the main peak and is less intense in all compounds except in **4NICz**, where the shoulder and peak have almost identical intensity. The trend in the λ_abs_ of the low‐energy band matches the trend in the predicted S_1_ energies, but the energies are generally more stabilized (**Table** **2**). The magnitude of the molar attenuation coefficient of the low energy band (ε) is roughly comparable for **4NICz**, **7NICz** and **6,10NICz** (ε = 8.0, 8.2, and 9.5 10^3^ M^−1^ cm^−1^, respectively), while it is approximately double and triple this value for **6NICz** and **5NICz** (ε = 14.0 10^3^ M^−1^ cm^−1^) and (ε = 22.9 10^3^ M^−1^ cm^−1^), respectively. Considering the moderate absorptivity and low energy of this band, we assign it to the SRCT absorption of the ring system (S_1_). The absorptivity data reported here in toluene are similar, both in trend and absolute values, to those previously reported by Kader *et al.* in dichloromethane.^[^ ^28^ ^]^

**Table 2:** Selected photophysical properties in dilute toluene solution.

Emitter	λ_abs,1_ / nm (shoulder)	ε_abs,1_ / 10^3^M^−1^cm^−1^ (shoulder)	λ_PL_ / nm (shoulder)	Stokes shift / nm (/ eV)	FWHM_PL_ / nm	Φ_PL_ ^a)^ / % (N_2_)	τ_P_ ^b)^ / ns
4NICz	360 (353)	8.0 (7.9)	374 (393)	14 (0.13)	27	6 (11)	8.9
5NICz	352 (340)		357 (371)	5 (0.05)	22	12 (24)	5.0
6NICz	374 (359)	14.0 (8.9)	384 (404)	10 (0.09)	24	50 (53)	7.5
7NICz	359 (345)	8.2 (6.0)	365 (381)	6 (0.06)	26	15 (24)	10.5
6,10NICz	382 (365)	9.5 (5.4)	391 (412)	9 (0.07)	23	12 (15)	4.8

^a)^ Photoluminescence quantum yield of degassed and aerated solutions;

^b)^ Prompt decay lifetime of emission, λ_exc_ = 375 nm. No delayed lifetimes were detected for any of the emitters. All spectra were obtained in optically dilute solutions (≈10^−5^ m). See Table S3 (Supporting Information) for full absorption data.

In contrast to the lowest energy band, which shifts with the varying position of the pyridine N, the energy of the medium‐energy band is largely invariant. It is most red‐shifted in **6NICz** at 323 nm, while it is the most blue‐shifted in **5NICz** at 312 nm, a difference of only 11 nm across all five structures. Similar to the profile of the lowest energy band, there is a blue‐shifted, lower intensity shoulder of the medium energy band in **4NICz**, **6NICz,** and **6,10NICz**, appearing ≈10 nm shorter in wavelength; however, this shoulder is absent from the spectra of **5NICz** and **7NICz**. The ratio of the intensity between the low‐energy and medium‐energy bands (Table S3, Supporting Information) trends very well with the ratio of the calculated oscillator strengths between the transitions to the S_1_ and S_2_ states (Table S2, Supporting Information. Specifically, *f*_S1_ and ε_abs,1_ are both less intense than *f*_S2_ and ε_abs,2_ in **4NICz** and **7NICz**, but *f*_S1_ and ε_abs,1_ are more intense than *f*_S2_ and ε_abs,2_ in **5NICz**, **6NICz**, and **6,10NICz**. Considering this, and that the calculated S_2_ energies are predicted to be invariant with molecular structure, we assign this medium energy band to a transition to S_2_.

**Figure 5:**
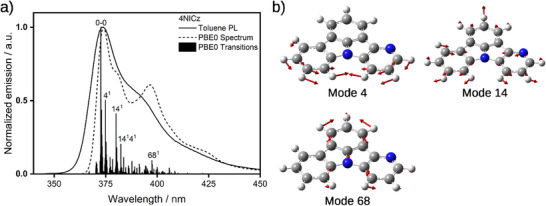
a) Comparison of the experimental PL spectrum in toluene and the vibrationally‐resolved PBE0 FCHT emission spectrum from the S_1_ excited state of **4NICz**. The computed spectrum has been red‐shifted by *≈*0.24 eV such that the peak maximum coincides with the experimental λ_PL_. b) Force vectors (red arrows) of the ground‐state vibrational modes with significant contribution to the FCHT spectrum.

The photoluminescence (PL) spectrum of each emitter in toluene shows a single, narrow, high‐energy band with a peak, λ_PL_, < 400 nm (Figure 4). The trend in the λ_PL_ mirrors that for the lowest‐energy absorption band and the calculated S_1_ energies, with the highest energy emitter being **5NICz** (357 nm), followed by **7NICz** (365 nm), **4NICz** (374 nm), **6NICz** (384 nm) and **6,10NICz** (391 nm). Each emitter exhibits a remarkably small Stokes shift of < 0.14 eV (< 15 nm, Table 2), which is consistent with both the short‐range charge transfer excited state character and their rigid structure. Having a mirror‐image profile to that of the lowest energy absorption band, the PL spectra have a lower intensity, lower energy shoulder, which is typical for vibrational sub‐states. Analysis of the vibronic coupling of the S_1_ state using DFT reveals that three vibrational modes are largely responsible for this spectral shape. Using **4NICz** as a model emitter (**Figure** **5**), two of these modes are in‐plane bends of the whole molecule around the central N (modes 4 and 14), one with an additional displacement of the top‐most ring (mode 14). Coupling to these modes results in a slight broadening of the main emission peak. Meanwhile, the shoulder at ≈390 nm originates from coupling to mode 68, which is an in‐plane stretch of several bonds throughout the molecule. This pattern is repeated in each of the five emitters (Figures S39 and S40, Supporting Information). Despite the broadening induced by the shoulder, the full width at half‐maximum (FWHM) of each emission band is extremely narrow, ranging from 27 nm in **4NICz**, to just 22 nm in **5NICz**. In air, the photoluminescence quantum yield, Φ_PL_, of the emitters ranges from 50% for **6NICz,** to 15% (**7NICz**) 12% (**5NICz**), 12% (**6,10NICz**), and 6% (**4NICz**). Under deaerated conditions, the Φ_PL_ of **4NICz**, **5NICz,** and **7NICz** increased by a factor of 1.8, 2.0, and 1.6 respectively, while the Φ_PL_ of **6NICz** and **6,10NICz** remained relatively constant, changing by only a factor of 1.1 and 1.3, respectively (Table 2). The decrease in Φ_PL_ under aerated conditions is caused by quenching of triplet excitons by triplet oxygen and is typical of TADF and/or RTP emitters.^[^ ^47^ ^]^

Analysis of the time‐resolved PL decays reveals the kinetics of the photoluminescence (Figure 4b). For all emitters, there is a prompt, monoexponential fluorescence decay process, with **6,10NICz** possessing the fastest decay with lifetimes (τ_PL_) of 4.8 ns, followed by **5NICz** at 5.0 ns, **6NICz** at 7.5 ns, **4NICz** at 8.9 ns, and the slowest being **7NICz** at 10.5 ns (Table 2). There is no discernible pattern between the photoluminescence lifetimes and structure. No delayed emission was detected from any of the emitters, indicating an absence of appreciable triplet‐related processes in this medium.

## 5 Polymethylmethacrylate (PMMA) Photoluminescence Study

We next investigated the photophysical properties of the emitters when dispersed in the inert polymeric host, polymethylmethacrylate (PMMA), first at a doping concentration of 10 wt.% emitters compared to the host. When recorded in air, the PL spectrum of each emitter was noticeably broader compared to that in toluene, which is unusual considering the suppression of molecular vibrations that are induced by the solid matrix compared to the free‐flowing solution. The degree of broadening experienced by each emitter is not constant, with **6NICz** and **5NICz** having a FWHM that is 3.0× and 2.6× wider than in toluene, respectively, while the FWHM of **6,10NICz** and **4NICz** are approximately twice as wide (1.9× and 1.8×, respectively), and the FWHM of **7NICz** remained relatively narrow, being only 1.5× wider than in toluene. This broadening is likely caused by contributions to the emission from aggregates, considering the relatively high doping concentration and the planar structure of the emitters. Because of this variable broadening, the λ_PL_ of the 10 wt.% doped films in PMMA no longer follows the expected pattern of **6/6,10NICz** > **4NICz** > **5/7NICz**, but in general, the PL of all the emitters is slightly red‐shifted. However, a similar pattern to the trend in λ_PL_ in toluene is observable in terms of the onset of the emission, except that **6NICz** and **6,10NICz** have almost identical onsets. The shoulder feature observed in the solution is largely absent in the film PL, with only **5NICz** retaining a significant shoulder in PMMA.

**Figure 6:**
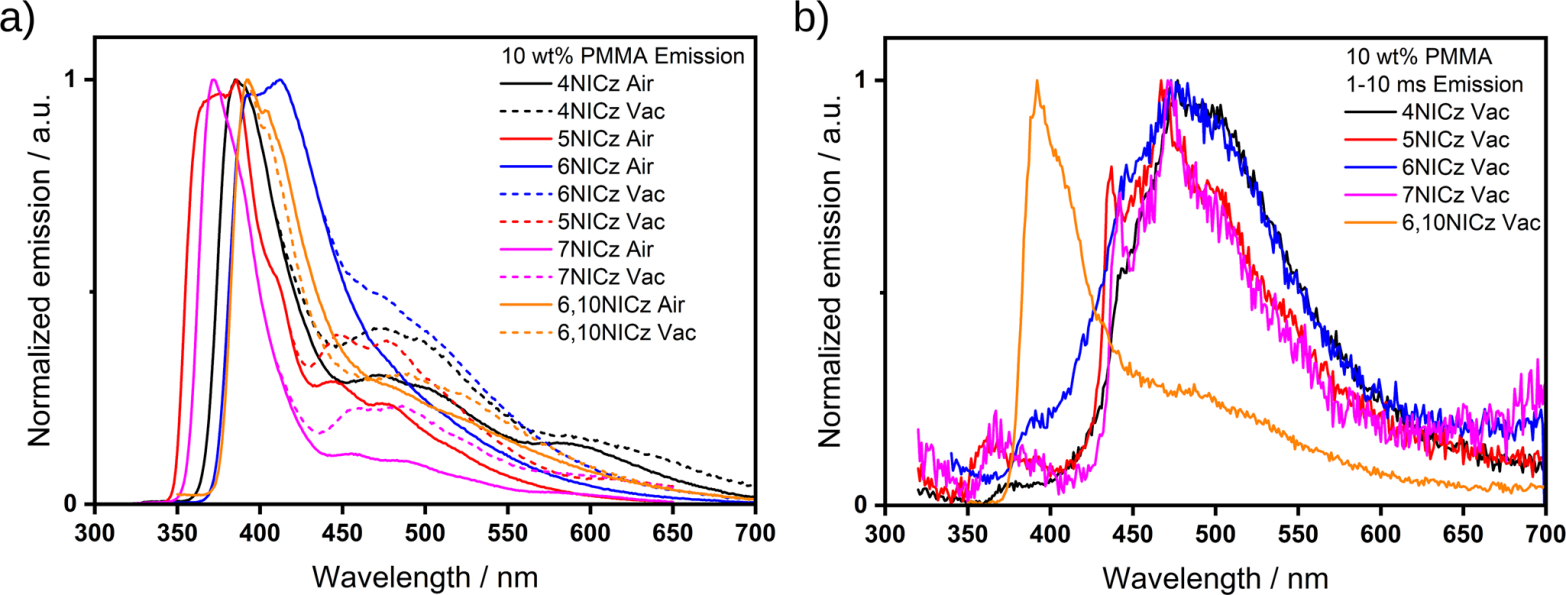
a) PL spectra of 10 wt.% doped films in PMMA in the air (solid lines) and under vacuum (dashed lines). The PL under vacuum has been cut at high energy where it matches the PL in the air for clarity, i.e. at ≈400 nm for **4NICz**, **5NICz**, **7NICz,** and **6,10NICz**, and 450 nm for **6NICz**. b) Time‐gated PL spectra (1–10 ms) under vacuum. λ_exc_ = 295 nm (**4NICz**), 280 nm (**5NICz**), 282 nm (**6NICz**), 294 nm (**7NICz**) and 278 nm (**6,10NICz**) for both a) and b).

Strikingly, each compound now exhibits a second, less‐intense emission band at longer wavelengths (**Figure** **6**a, solid lines). In **6NICz** and **6,10NICz** this band is partially masked by the tail of the higher energy emission, assigned to emission from S_1_, but in **4NICz**, **5NICz,** and **7NICz** distinct peaks can be observed at *≈*450, 475, and 500 nm, with an additional peak at 590 nm in **4NICz** only. Under vacuum (**Figure** 6a, dashed lines), the intensity of this low‐energy band increases significantly relative to the intensity of the S_1_ band, and clear peaks can now be observed in the steady‐state PL spectra of all five emitters. The shape of the S_1_ band, meanwhile, is unaffected by the removal of oxygen, except in **6,10NICz**, which is slightly narrower under vacuum. The onset of emission is identical in all cases under both aerated and deaerated conditions.

Time‐gated PL spectra acquired between 1 and 10 ms after excitation do not capture the prompt fluorescence, detecting only delayed emission from long‐lived excited states (Figure 6b). Here, a broad emission band is observed with a peak at ≈470 nm in **4NICz**, **5NICz**, **6NICz**, and **7NICz** (**Table** **3**). The time‐gated PL spectrum of **6,10NICz**, meanwhile, shows significant delayed emission at the same energy as that observed in the steady‐state PL spectrum, and which largely masks the emission from any lower‐energy PL process; however, a small shoulder can still be observed at ≈486 nm. In **4–7NICz**, the peak of the low‐energy band varies little with structure, ranging from 467 nm (**5NICz**) to 479 (**6NICz**), and in **5NICz** and **7NICz** it is well resolved into a second peak at higher energy, at 438 and 442 nm, respectively. We assign this low‐energy process to room temperature phosphorescence from T_1_, for the following reasons: 1) the band is at lower energy than the fluorescence (S_1_) band; 2) it is observed as delayed PL; and 3) the λ_PL_ is approximately invariant with structure, matching the trend in T_1_ energies predicted by computations. Further evidence for this assignment is provided in the emission behavior of the 1 wt.% doped films in PMMA (vide infra).

**Table 3:** Selected photophysical properties in 10 wt.% doped films in PMMA.

Emitter	λ_PL,Fl_ ^a)^ / nm	λ_PL,Ph_ ^b)^ / nm (shoulder)	FWHM_PL_ / nm (Vac)	λ_DE,Ph_ ^c)^ / nm (2^nd^ Peak)	Φ_PL_ ^d)^ / % (Vac)	τ_p_ ^e)^ / ns	τ_d_ ^f)^ / ms
4NICz	385	472	47 (50)	477	15 (15)	9.8	‐
5NICz	386	448 (478)	58 (58)	467 (437)	23 (26)	3.3	‐
6NICz	412	475 (505)	72 (86)	479	31 (35)	4.7	‐
7NICz	372	483 (457)	38 (38)	472 (442)	23 (25)	10.6	‐
6,10NICz	392	485 (523)	44 (52)	≈486	10 (12)	3.7	11.9/0

^a)^ Peak of the S_1_ steady‐state band under air;

^b)^ Apparent peak of the steady‐state T_1_ band under vacuum;

^c)^ Peak of the phosphorescence, as determined by gated emission under vacuum at 1–10 ms after excitation;

^d)^ PLQY of the entire PL of the films in air and under vacuum;

^e)^ Prompt decay lifetime of the fluorescence, λ_exc_ = 375 nm;

^f)^ Delayed decay lifetime of the S_1_ emission, λ_exc_ = 278 nm. Lifetimes of the phosphorescence were not recorded in this medium, see 1 wt.% doped films in PMMA (**Table** **4**).

In air, the trend found for the Φ_PL_ of the emitters in 10 wt.% doped films in PMMA differs slightly from that measured in toluene (Table 3). The positions of **6,10NICz** and **4NICz** are now reversed, where **6,10NICz** shows the lowest Φ_PL_ (10%), followed by **4NICz** (15%). Meanwhile, **5NICz** and **7NICz** have an equivalent Φ_PL_ of 23%, whereas in toluene, **7NICz** was slightly brighter, and **6NICz** remains the brightest at 31%. The average Φ_PL_ across all five emitters remains the same as in toluene (20% in PMMA, 19% in toluene), but the spread in Φ_PL_ values is reduced. There is effectively no change in Φ_PL_ under deaerated conditions in 10 wt.% doped films in PMMA.

**Figure 7:**
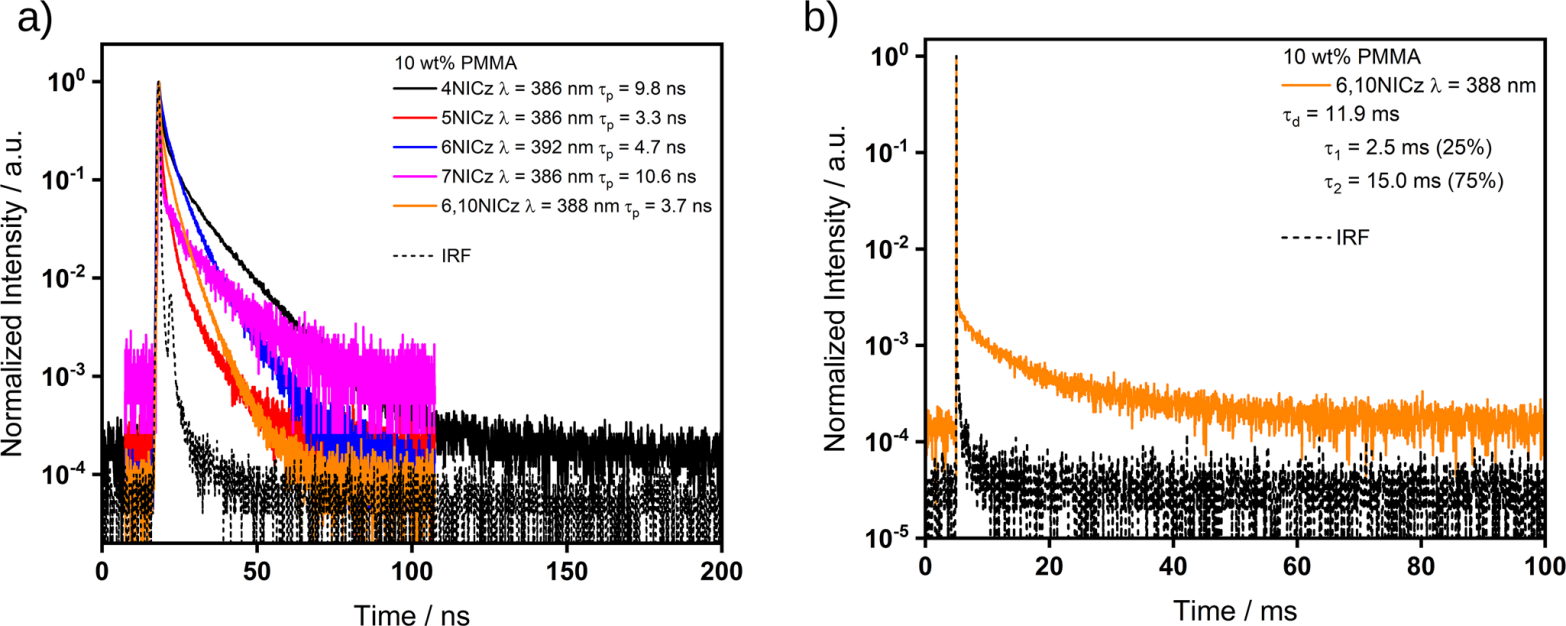
a) Time‐resolved decays of the prompt emission of the emitters in 10 wt.% doped films in PMMA, λ_exc_ = 375 nm, collected by TCSPC. b) Time‐resolved decays of the delayed emission of **6,10NICz** in 10 wt.% doped films in PMMA, collected by multi‐channel scaling (MCS), λ_exc_ = 278 nm. The lifetimes given in a) are the average lifetimes of the decay, a breakdown of individual fitting parameters is available in Table S4 (Supporting Information). The lifetimes given in b) are the biexponential fitting parameters of the decay. λ values in the legend indicate the emission collection wavelength for each compound.

Time‐resolved PL decay of the prompt emission shows biexponential (**4NICz**, **6NICz**, and **7NICz**) or triexponential (**5NICz** and **6,10NICz**) decay kinetics, with average lifetimes that are comparable to those measured in toluene (**Figure** **7**a, Tables 2 and 3). The trend in the magnitude of the prompt lifetimes of the 10 wt.% doped films in PMMA is also the same as that in toluene, except that **5NICz** has a shorter lifetime than **6,10NICz** in the doped PMMA film, while the opposite is true in toluene. Finally, **6,10NICz** exhibits delayed fluorescence, with a lifetime, τ_d_, of 11.9 ms, which accounts for the high‐energy band observed in the gated PL spectrum (Figure 6b). We attribute this delayed emission to TADF. Evidence for this assignment is given when we discuss our findings for the 1 wt.% PMMA films (vide infra), but for now we note that **6,10NICz** exhibits simultaneous TADF/RTP biluminescence at 10 wt.% doping in PMMA films. No delayed fluorescence emission was observed for the other emitters at room temperature.

**Figure 8:**
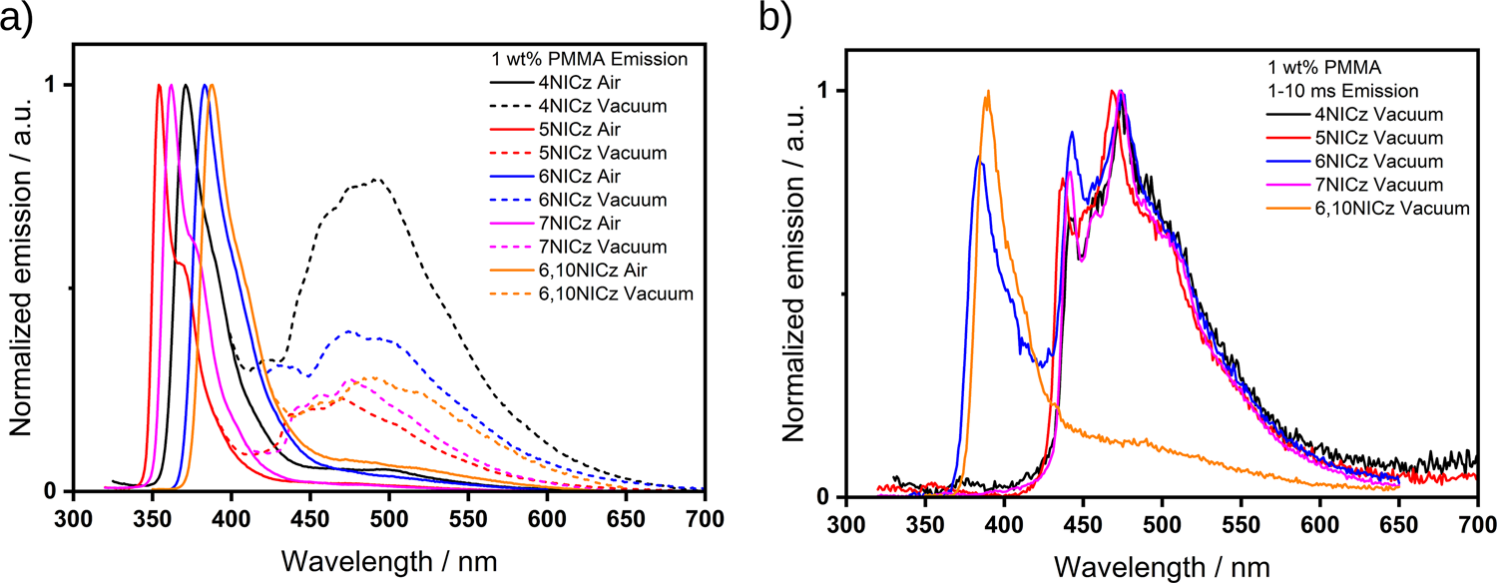
a) PL spectra of 1 wt.% doped films in PMMA in the air (solid lines) and under vacuum (dashed lines). For clarity, the PL under vacuum has been cut at high energy where it matches the PL in air, i.e., at ≈400 nm for **4NICz**, **5NICz**, and **7NICz**, and 425 nm for **6NICz** and **6,10NICz**. b) Time‐gated (1–10 ms) PL spectra under vacuum. λ_exc_ = 295 nm (**4NICz**), 280 nm (**5NICz**), 283 nm (**6NICz**), 294 nm (**7NICz**) and 278 nm (**6,10NICz**) for both a) and b).

We next prepared films at 1 wt.% emitter doping to mitigate against the formation of aggregates and their potential contribution to the photophysical picture. In air, the steady‐state PL spectra were significantly narrowed (**Figure** **8**a, Table 4) compared to those of the 10 wt.% doped films, with FWHM of 30 nm or less for all five emitters. The PL spectra were also blue‐shifted by *≈*10 nm, both in terms of the λ_PL_ and the onset, and the expected relationship between the λ_PL_ and molecular structure was re‐established. Further, the broad, low‐energy emission band at *≈*470 nm is absent at this doping concentration in the presence of oxygen. Yet, under vacuum, the low‐energy band returns with an equal or greater intensity to that observed in the 10 wt.% doped film, relative to the high‐energy band (Figure 8a, dashed line). This highlights the intense oxygen sensitivity of this emission process, which we assign to the same room‐temperature phosphorescence from T_1_ that we observed in the 10 wt.% doped film. Under vacuum, the time‐gated PL spectra (1–10 ms) of **4NICz**, **5NICz**, **6NICz,** and **7NICz** exhibit the same broad phosphorescence that was observed in the 10 wt.% doped films, but here the band is structured and clearly resolved into two distinct peaks in each of these four emitters. The positions of these peaks are largely invariant with structure, with a minor peak at ≈440 nm and a major peak at ≈470 nm. For **6,10NICz**, the phosphorescence remains largely hidden by the tail of the delayed fluorescence emission at λ_PL_ of 389 nm, a slight bump is nonetheless observed at *≈*485 nm corresponding to emission from T_1_. Meanwhile, the time‐gated PL spectrum of **6NICz** shows delayed fluorescence at 384 nm which is approximately equal in intensity to the phosphorescence at 473 nm. This behavior is distinct from the 10 wt.% doped films in PMMA, where only delayed emission from T_1_ was observed. Therefore, at this lower doping concentration in PMMA, both **6NICz** and **6,10NICz** exhibit dual TADF/RTP emission at room temperature.

**Table 4:** Selected photophysical properties in 1 wt.% doped films in PMMA.

Emitter	λ_PL,Fl_ ^a)^ / nm (shoulder)	λ_PL,Ph_ ^b)^ / nm (shoulder)	FWHM_PL_ / nm	λ_DE,Ph_ ^c)^ / nm (2nd Peak)	τ_p_ ^d)^ / ns	τ_d_ ^e)^ / ms	τ_Ph_ ^f)^ / s
4NICz	371	492 (461)	30	474 (443)	–^g)^	–^h)^	–^i)^
5NICz	354 (369)	470 (439)	24	468 (437)	–^g)^	–^h)^	1.5
6NICz	384	473 (500)	31	474 (443)	3.6	489.2	0.8
7NICz	362 (376)	475 (455)	27	473 (441)	–^g)^	–^h)^	1.9
6,10NICz	388	487 (521)	30	≈485	2.9	34.0	–^j)^

^a)^ Peak of the S_1_ steady‐state band in air;

^b)^ Apparent peak of the steady‐state T_1_ band under vacuum;

^c)^ Peak of the phosphorescence, as determined by the time‐gated emission (1 – 10 ms), under vacuum;

^d)^ Prompt decay lifetime of the fluorescence;

^e)^ Delayed decay lifetime of the fluorescence;

^f)^ Delayed decay lifetime of the phosphorescence;

^g)^ Not recordable due to the peak position being higher in energy than the excitation source;

^h)^ Not observed;

^i)^ Not recordable in the maximum time window offered by the spectrometer;

^j)^ Not recordable due to the very low intensity.

**Figure 9:**
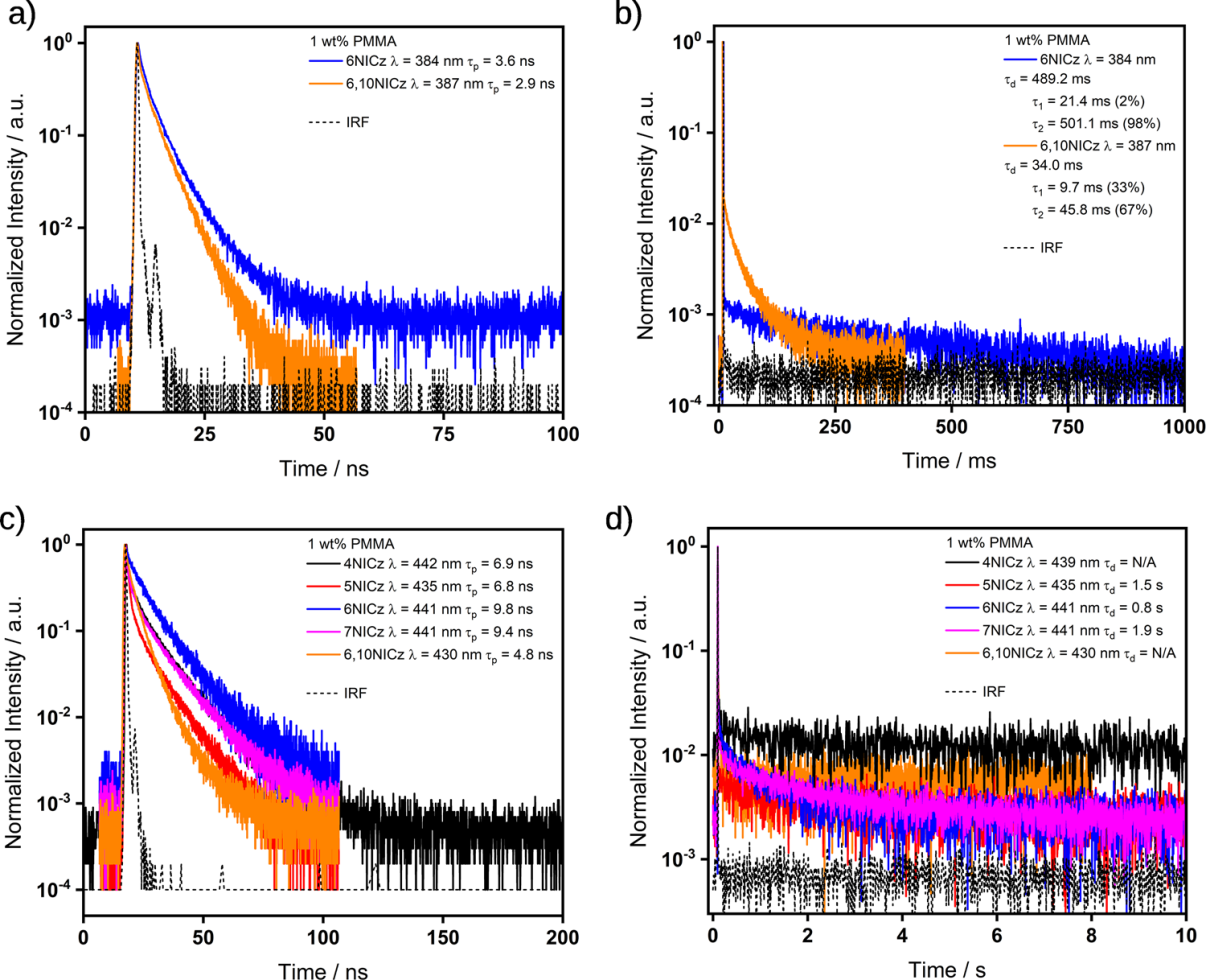
Time‐resolved decays of a) prompt emission (380 – 390 nm), b) delayed emission (380 – 390 nm), c) prompt emission (420 – 450 nm), and d) delayed emission (420 – 450 nm) in 1 wt.% doped PMMA films. λ_exc_ = 375 nm (a and c), 283 nm (**4NICz**, b, and d), 280 nm (**5NICz**, b, and d), 283 nm (**6NICz**, b, and d), 294 nm (**7NICz**, b and d), 278 nm (**6,10NICz**, b and d). Decays in a) and c) were collected by TCSPC, and b) and d) using the multi‐channel scaling (MCS) technique. The lifetimes given are the average lifetimes of the decay, a breakdown of individual fitting parameters is available in Tables S5 and S6 (Supporting Information).

**Figure 10:**
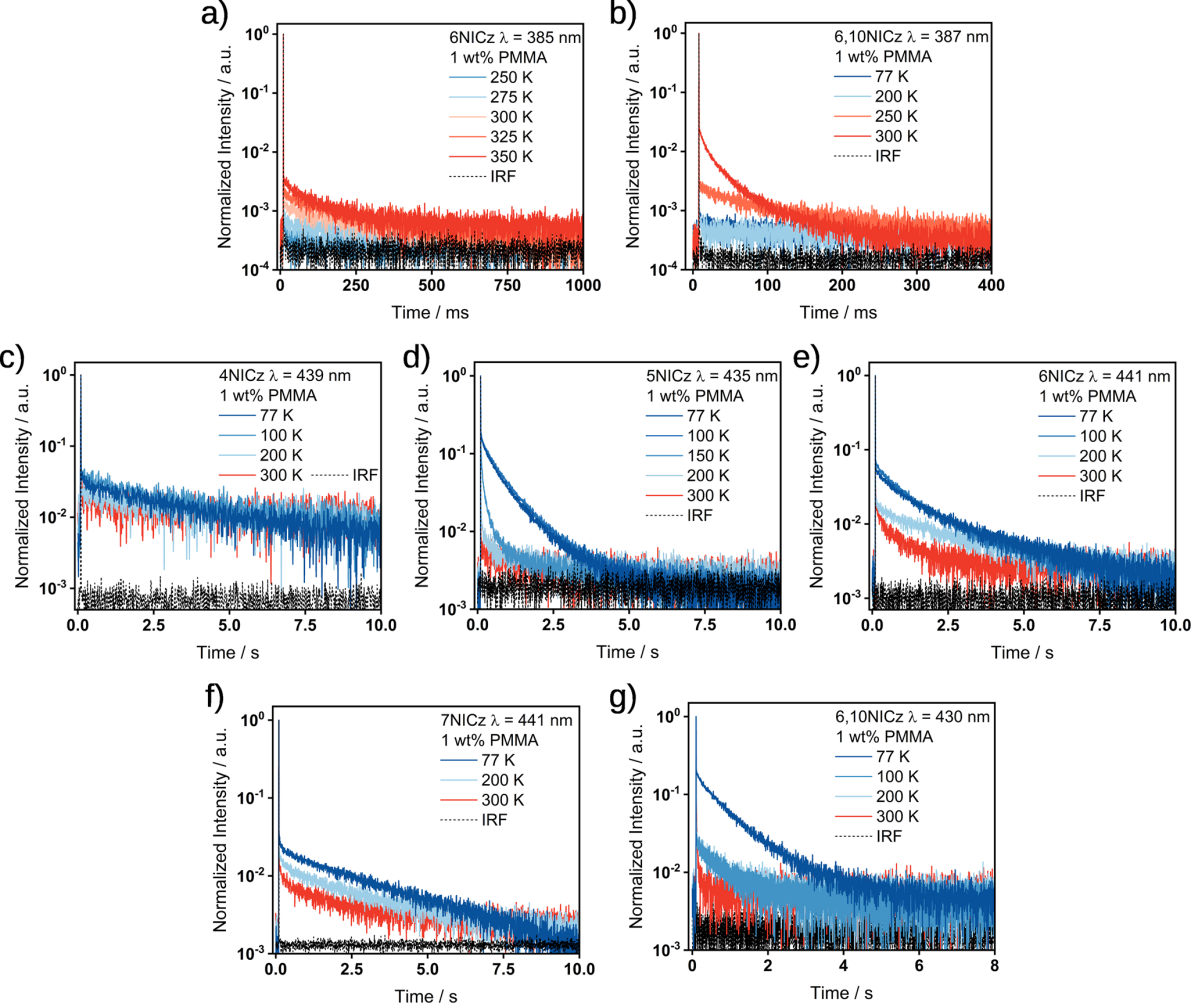
Temperature‐dependant decay processes of the 1 wt.% doped PMMA films of a) TADF of **6NICz**, *λ_exc_* = 283 nm; b) TADF of **6,10NICz**, *λ_exc_* = 278 nm; c) Phosphorescence of **4NICz**, *λ_exc_* = 295 nm; d) Phosphorescence of **5NICz**, *λ_exc_* = 280 nm; e) Phosphorescence of **6NICz**, *λ_exc_* = 283 nm; f) Phosphorescence of **7NICz**, *λ_exc_* = 294 nm; g) Phosphorescence of **6,10NICz**, *λ_exc_* = 278 nm. All decays were recorded using the multi‐channel scaling (MCS) technique.

Unfortunately, the λ_PL_ of each of **4NICz**, **5NICz,** and **7NICz** is shorter in wavelength than the pulsed laser excitation source of the fluorimeter, and so reliable decay profiles for the prompt fluorescence could not be obtained for these emitters. However, the lifetimes of the prompt fluorescence of **6NICz** and **6,10NICz** (**Figure** **9**a) are 1.3× faster than those of the 10 wt.% doped films in PMMA, at 3.6 and 2.9 ns, respectively. There is also a delayed fluorescence with τ_d_ of 489.2 and 34.0 ms for **6NICz** and **6,10NICz**, respectively (Figure 9b). Compared to the delayed fluorescence observed in the 10 wt.% doped films in PMMA, the τ_d_ of **6,10NICz** is 2.9× slower in the 1 wt.% doped films. The ‘switching‐on’ of TADF at this lower doping concentration in **6NICz** and the simultaneous slowing of the TADF process in **6,10NICz** highlight the complex interplay of the competing decay processes in these molecules, and the sensitivity of the photophysics to the environment. In the time‐gated PL spectra, all five emitters exhibited phosphorescence associated with extremely long lifetimes extending to the time scale of seconds (Figure 9d). Notably, the phosphorescence of **4NICz** was too long‐lived to be accurately fitted over a 10 s time window, but it could be readily observed with the naked eye (Figure S40, Supporting Information); additionally, the phosphorescence of **6,10NICz** at room temperature was too weak for the decay to be accurately fitted, but the tail of the decay can still be observed in the temperature‐dependent TRPL measurement even at room temperature (Figure 10g). Phosphorescence lifetimes for **4NICz** and **6,10NICz** are thus not reported. The phosphorescence lifetimes, τ_Ph_, of **5NICz**, **6NICz**, and **7NICz** are 1.5, 0.8, and 1.9 s at room temperature. Finally, a much shorter component to the decay associated with the phosphorescence was recorded over 100/200 ns (Figure 9c) in all five emitters. We attribute this process to residual prompt fluorescence, considering that the tail of the higher energy emission band clearly extends to overlap with the phosphorescence band (Figure 8a); we have included the decay and fitted lifetime of this process for completeness. Compared to the prompt fluorescence lifetime, the lifetimes of this “fast” process are 2.7× and 2.0× slower for **6NICz** and **6,10NICz**, respectively, at 9.8 and 4.8 ns.

To fully assign the origin of the delayed emission, we next recorded the change in delayed lifetimes with temperature. The delayed fluorescence observed in **6NICz** and **6,10NICz** is noticeably faster at elevated temperatures (**Figure** **10**a,b), and is assigned as TADF. As all five emitters exhibit negligible solvatochromism (Figure S39, Supporting Information), this process can be assigned as SRCT emission, which is characteristic of MR‐TADF. The ΔE_ST_ of **6NICz** and **6,10NICz** in 1 wt.% doped films in PMMA, measured from the difference in the onset of the steady‐state PL and time‐gated emission (1–10 ms) at 77 K (Figure S41, Supporting Information), is 0.46 and 0.36 eV, respectively, which explains the very long delayed fluorescence lifetimes observed in this medium. The ΔE_ST_ of **4NICz**, **5NICz,** and **7NICz** are 0.59, 0.59, and 0.65 eV, respectively, which are too large for efficient TADF. We note that the ΔE_ST_ of **5NICz** is likely to be slightly underestimated, due to the broadening of the phosphorescence spectrum at low temperature, which makes measuring the true onset difficult. Despite this, the trend in ΔE_ST_ largely matches that predicted by computations, with **6NICz** and **6,10NICz** having the smallest ΔE_ST_, followed by **4NICz** and **5NICz** and then **7NICz** having the largest ΔE_ST_.

**Figure 11:**
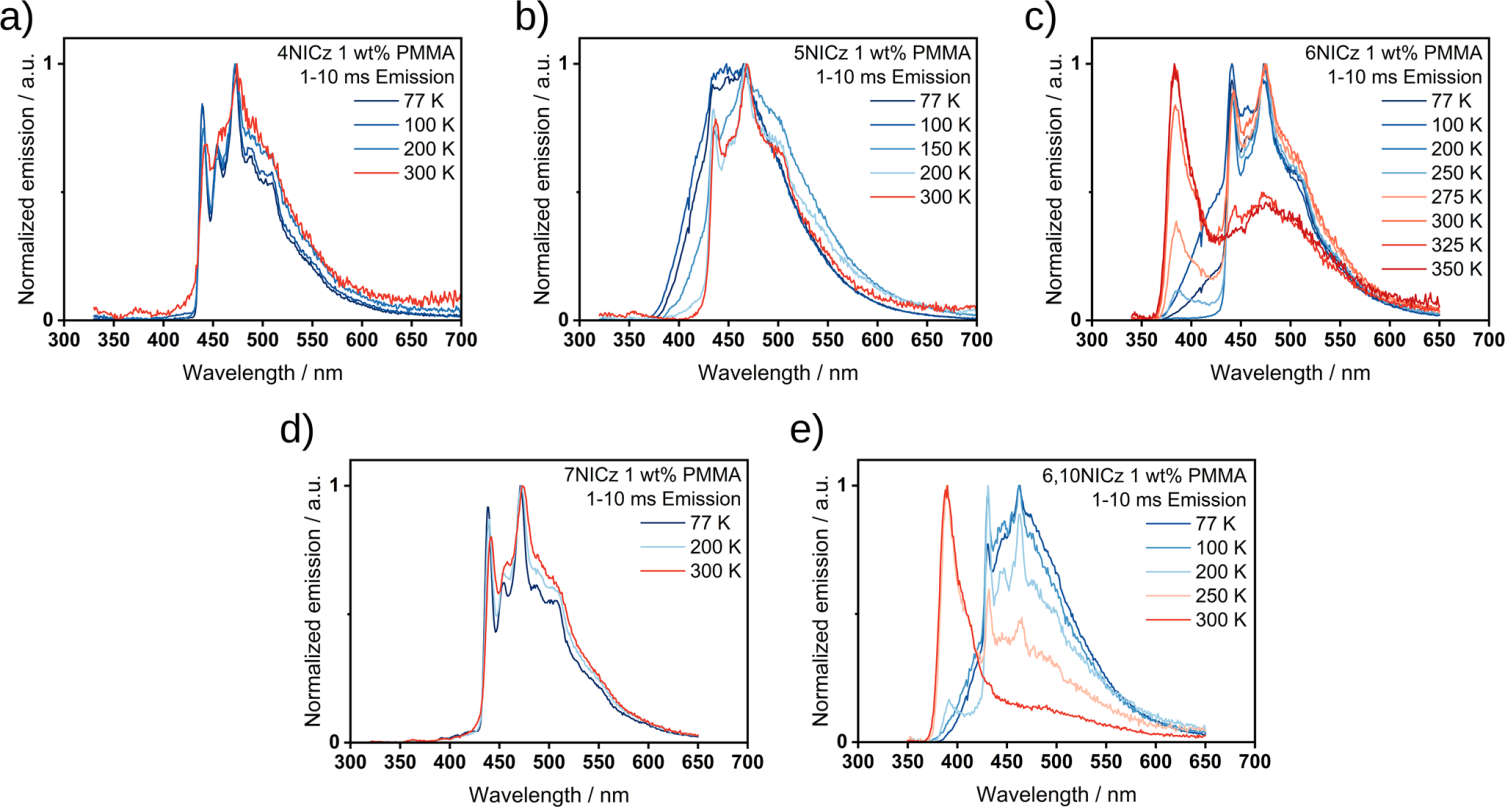
The change in the time‐gated PL spectra (1–10 ms) of a) **4NICz**, b) **5NICz**, c) **6NICz**, d) **7NICz**, and e) **6,10NICz** in 1 wt.% doped PMMA films as a function of temperature under vacuum. λ_exc_ = 295 nm (**4NICz**), 280 nm (**5NICz**), 283 nm (**6NICz**), 294 nm (**7NICz**) and 278 nm (**6,10NICz**).

Meanwhile, the intensity of the low energy phosphorescence in all five emitters shows a characteristic inverse temperature dependence (Figure 10c–g). To confirm that the room‐temperature phosphorescence originates from the T_1_ state, we additionally recorded the change in the time‐gated PL spectra with temperature (**Figure** **11**). For **4NICz** and **7NICz**, the phosphorescence spectra do not change between 77 and 300 K, while for **5NICz** the λ_Ph_ remains the same, but the PL spectra are broadened at lower temperatures. In **6NICz** and **6,10NICz**, the shape of the phosphorescence at 300 K is partially masked by the contribution from the higher energy TADF, but the bump originating from RTP clearly matches the peak position of the phosphorescence at 77 K. The time‐gated PL spectra of **6NICz** in particular showcase the competition between TADF and phosphorescence, with the intensity of the TADF decreasing and the intensity of the phosphorescence increasing with decreasing temperature (Figure 11c). In all cases, the phosphorescence at 77 K matches that observed at room temperature, and so the phosphorescence can be assigned to the radiative decay from the T_1_ state.

**Figure 12:**
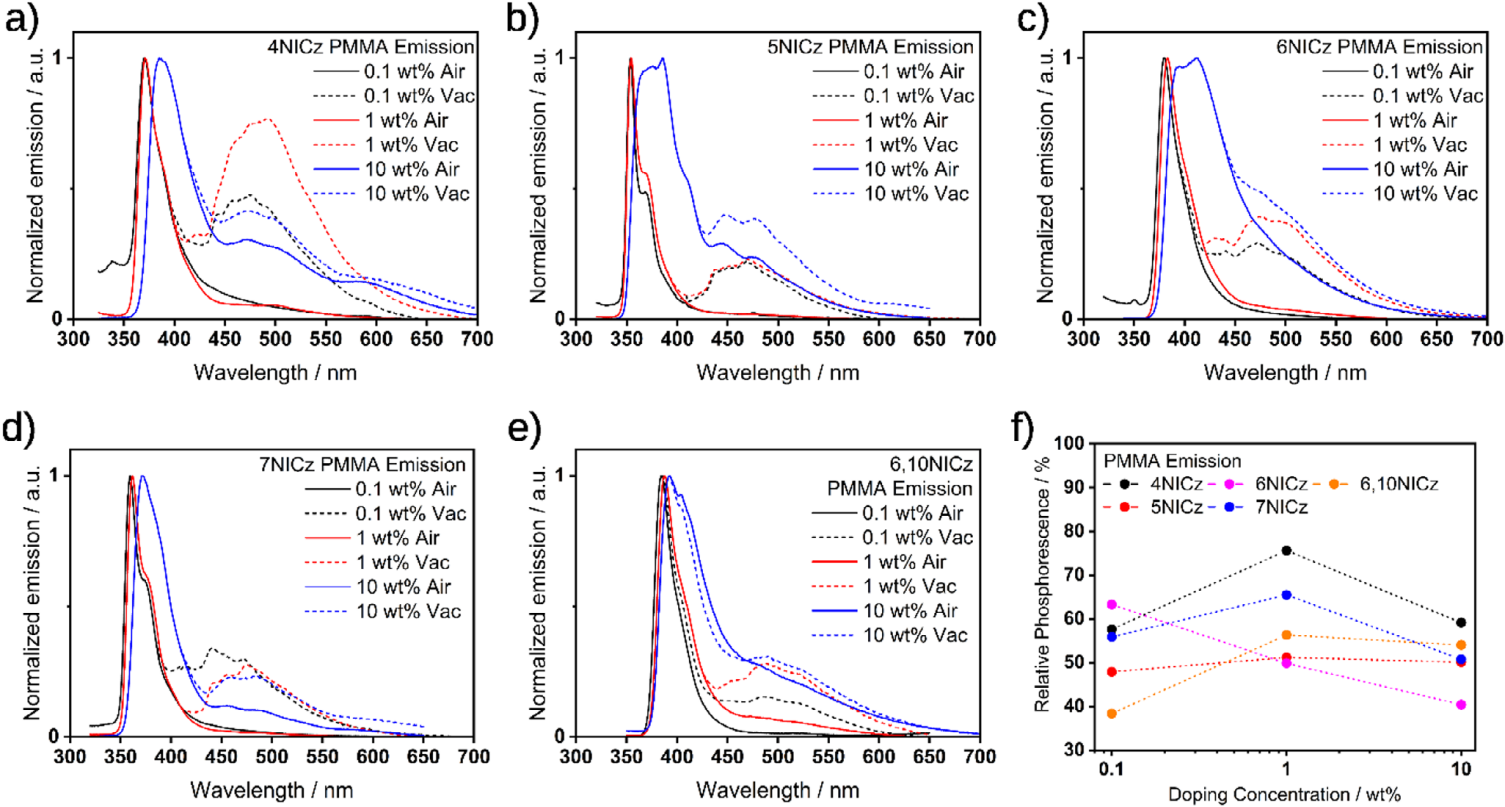
The change in the PL spectra of a) 4NICz, b) 5NICz, c) 6NICz, d) 7NICz, and e) 6,10NICz in doped PMMA films for different emitter concentrations. λ_exc_ = 295 nm (**4NICz**), 280 nm (**5NICz**), 283 nm (**6NICz**), 294 nm (**7NICz**) and 278 nm (**6,10NICz**). The change in gated PL is shown in Figure S42 (Supporting Information). Figure f) shows the relative proportion of phosphorescence to the total PL for each spectrum as determined by the Gaussian fitting shown in Figure S46 (Supporting Information).

To ensure that there is no possibility that emission from aggregates contributes to the observed photophysics, we next prepared 0.1 wt.% doped films in PMMA. The PL spectra both in air and under vacuum are comparable to the 1 wt.% doped film spectra (**Figure** **12**a–e, black and red lines), with the primary difference being the ratio of the fluorescence to phosphorescence under vacuum. In **4NICz**, **6NICz**, and **6,10NICz**, there is a decrease in the proportion of phosphorescence to fluorescence in the 0.1 wt.% doped films compared to the 1 wt.% doped films (Figure 12f), but in **7NICz** this is reversed, with a noticeable increase in the phosphorescence relative to fluorescence in the 0.1 wt.% doped films. There is negligible change in the ratio of fluorescence to phosphorescence across any of the doping concentrations tested for **5NICz**. The time‐gated PL also corresponds well to the 1 wt.% doped films in PMMA (Figure S42, Supporting Information), and so the same biluminescence behavior is preserved at 0.1 wt.% emitter concentration. Exceptionally, **5NICz** uniquely has residual fluorescence in the time‐gated PL only at 0.1 wt.%. However, the brightness of this film was very low and there is significant background scattering from the emission source present in the spectrum, so it is difficult to ascertain the true intensity and nature of this ‘delayed’ fluorescence. The strong sensitivity of the phosphorescence to the presence of oxygen is conserved in the 0.1 wt.% doped films; for example, **6,10NICz** shows no phosphorescence in air (Figure 12e). Upon increasing the doping concentration to 10 wt.%, there is a red‐shift in the PL (vide supra), and the relative intensity of the fluorescence and phosphorescence continues to change (Figure 12a–e, blue line). Due in part to the spectral broadening of the fluorescence, the PL spectra of the 10 wt.% doped films of **4NICz**, **6NICz**, and **7NICz** each exhibit a decrease in the relative proportion of phosphorescence compared to their respective 1 wt.% films, while the PL spectra of **5NICz** and **6,10NICz** show no change, highlighting the non‐linear dependence of the RTP on doping concentration. Notably, all the emitters exhibit the greatest proportion of phosphorescence in either the 1 wt.% (**4NICz**, **5NICz**, **6NICz**, and **6,10NICz**) or 0.1 wt.% films (**7NICz**). For completeness, we have also recorded the steady‐state and time‐gated PL of the neat films (Figure S43, Supporting Information), which show distinct emission behavior from the doped films in PMMA.

We summarize the photophysics of the doped films in PMMA as follows: At 10 wt.%, the PL is broadened, red‐shifted, and less well resolved compared to lower doping concentrations or solution measurements in toluene due to the influence of intermolecular emission from aggregates. All five of the emitters are biluminescent, with simultaneous emission from the S_1_ and T_1_ excited states, in the form of fluorescence/phosphorescence for the singly substituted emitters (**4NICz**, **5NICz**, **6NICz**, and **7NICz**) and MR‐TADF/phosphorescence for **6,10NICz**. The PL spectra at 1 and 0.1 wt.% are equivalent and result from emission from monomolecular species, resulting in narrower, blue‐shifted, and better resolved spectra. The biluminescence behavior is preserved in the 1 wt.% doped films except that MR‐TADF has been switched on in **6NICz** in addition to **6,10NICz**. Finally, the proportion of RTP (relative to fluorescence) shows non‐linear dependence on the doping concentration, while the oxygen sensitivity of the RTP is greatest at 1 wt.%, of the concentrations tested here.

## 6 Mechanistic Overview and Kinetics Insights

**Figure 13:**
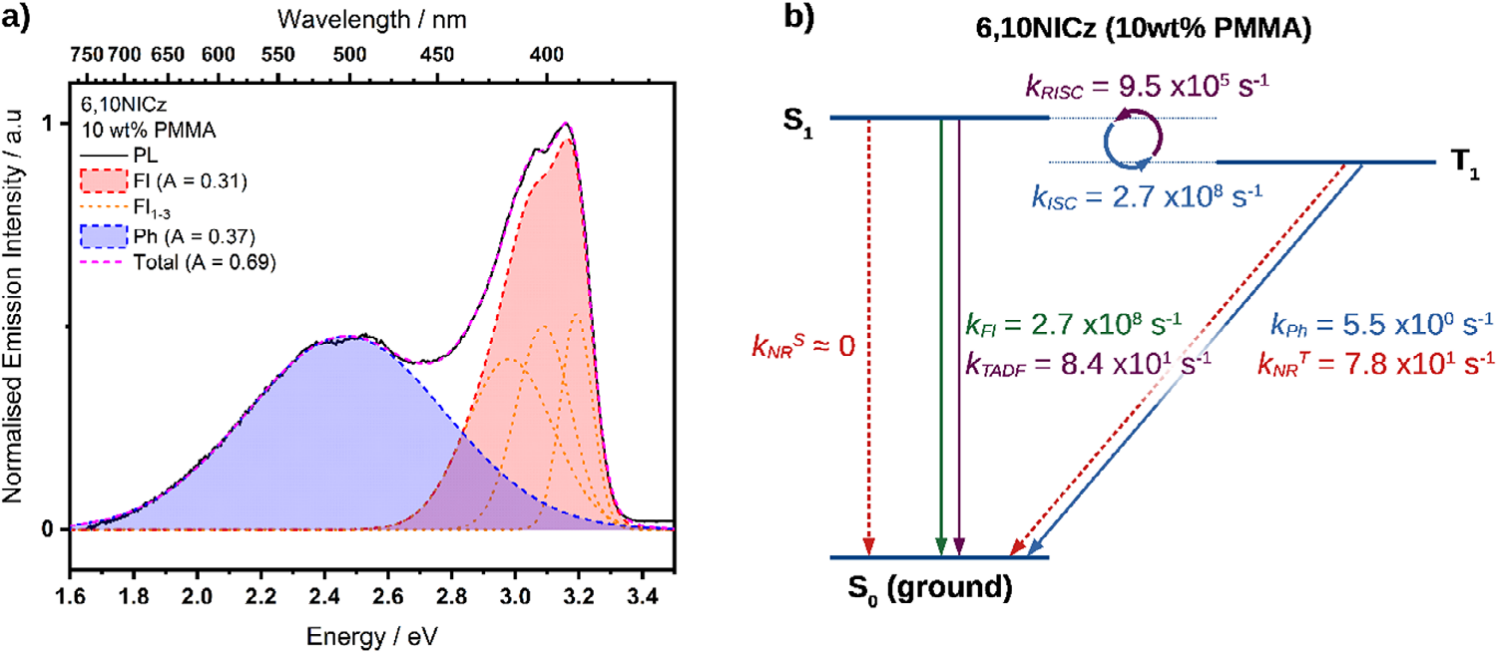
a) Gaussians fitted to the PL spectrum of the 10 wt.% doped film of **6,10NICz** in PMMA. The fluorescence component was fitted with 3 Gaussians (Fl_1_, Fl_2_, and Fl_3_), and the phosphorescence component was fitted to only one. A: area in arbitrary units. b) derived kinetics parameters of the emission of **6,10NICz**. See the Supporting Information for the fitting and kinetics methodology.

In this section, we have chosen to focus on **6,10NICz** as a model system to provide greater insight into the competing emission mechanisms of these biluminescent emitters. By fitting the spectral profile of the PL to a number of Gaussian functions, it is possible to elucidate the relative contributions of the Ph and Fl. As a 10 wt.% dopant in PMMA (under vacuum), we find an almost equal proportion of Fl (46%) to Ph (54%) (**Figure** **13**a). By applying the kinetics model proposed by Tsuchiya *et al.* to this ratio, along with the observed τ_p_, τ_d_, and Φ_PL_, we are then able to estimate the relevant kinetics parameters of the emission processes (Figure 13b).

Following photoexcitation to S_1_, the excitons radiatively decay to S_0_ (*k_Fl_*) and non‐radiatively to T_1_ (*k_ISC_*) at an equivalent rate of 2.7 ×10^8^ s^−1^. This arrangement is typical for TADF emitters due to the competition between *k_Fl_* and *k_ISC_*, and the requirement to populate T_1_ in order to later observe TADF (under photoexcitation, the general assumption is that only the singlet state is directly populated). From the T_1_ state, the excitons can decay via 3 pathways, either by thermal upconversion back to S_1_ (*k_RISC_*), radiative decay to S_0_ (*k_Ph_*), or nonradiative decay to S_0_ (*k_NR_^T^*). Of these, RISC to S_1_ is by far the fastest with a *k_RISC_* of 9.5 ×10^5^ s^−1^, providing a pathway for TADF. However, much of the upconverted exciton population rapidly transits back to T_1_ (via ISC) in a cycle, as *k_ISC_* is competitive with *k_Fl_* and is three orders of magnitude faster than *k_RISC_*. The net effect of this interaction is a long‐lived population of triplet excitons, which slowly decays via delayed fluorescence. Although this is typical for TADF, the magnitude of the discrepancy between *k_ISC_* and *k_RISC_* is responsible for the relatively long delayed fluorescence lifetime of 11.9 ms. The next fastest decay process from T_1_ is non‐radiative loss to the ground state with a *k_NR_^T^* of 7.8 ×10^1^ s^−1^, which is an order of magnitude faster than the phosphorescence rate constant (*k_Ph_*) of 5.5 s^−1^. Although the *k_NR_* is very slow, this discrepancy between *k_NR_* and *k_Ph_*, as well as the much faster *k_ISC_* compared to *k_RISC_* is ultimately responsible for the low Φ_PL_ of 12%, as this non‐radiative channel (*k_NR_* = 7.8 ×10^1^ s^−1^) competes with both phosphorescence (*k_Ph_* = 5.5 s^−1^) and TADF (*k_TADF_* = 8.4 ×10^1^ s^−1^). Notably, because *k_Fl_* and *k_ISC_* are so fast, the population of S_1_ at any given moment is extremely small, and so there is negligible non‐radiative decay from S_1_ (*k_NR_^S^* ≈ 0 s^−1^).

The kinetics of **6,10NICz** highlight many of the important design criteria for achieving TADF‐biluminescent materials:

1. *k_ISC_* and *k_Fl_* must have comparable magnitudes. If *k_ISC_* is significantly faster than *k_Fl_*, then fluorescence will be deactivated and only phosphorescence will be observed, as in RTP‐only materials (or else no emission at all if *k_Ph_* ≈ 0). If *k_Fl_* dominates, then communication with the triplet manifold is lost, and the material becomes fluorescent only.
2. The relative magnitudes of *k_ISC_* and *k_RISC_* must be carefully balanced to ensure that both TADF and phosphorescence are observed. If *k_RISC_* is too fast (and/or *k_ISC_* is too slow) then TADF will dominate and too little triplet population will remain for appreciable phosphorescence. If *k_RISC_* is too slow, then the rate of upconversion will be too slow for TADF. This criterion will generally result in relatively long delayed fluorescence lifetimes, as observed in **6,10NICz** (11.9 ms), and *intermediate* values of Δ*E*_ST_ that are larger than generally expected for TADF materials (0.36 eV for **6,10NICz**).
3. *k_NR_^T^* should be minimized as much as possible and certainly should be slower than *k_Ph_*. Otherwise, Φ_PL_ will trend toward zero, and the long‐lived triplet processes in particular (TADF, phosphorescence) are liable to deactivation. As in RTP‐only materials, the most practical method to achieve this remains immobilization in an inert matrix such as PMMA, and it is likely for this reason that neither TADF nor phosphorescence was observed in toluene for any of the emitters.

## 7 Structure‐Property Relationship

**Figure 14:**
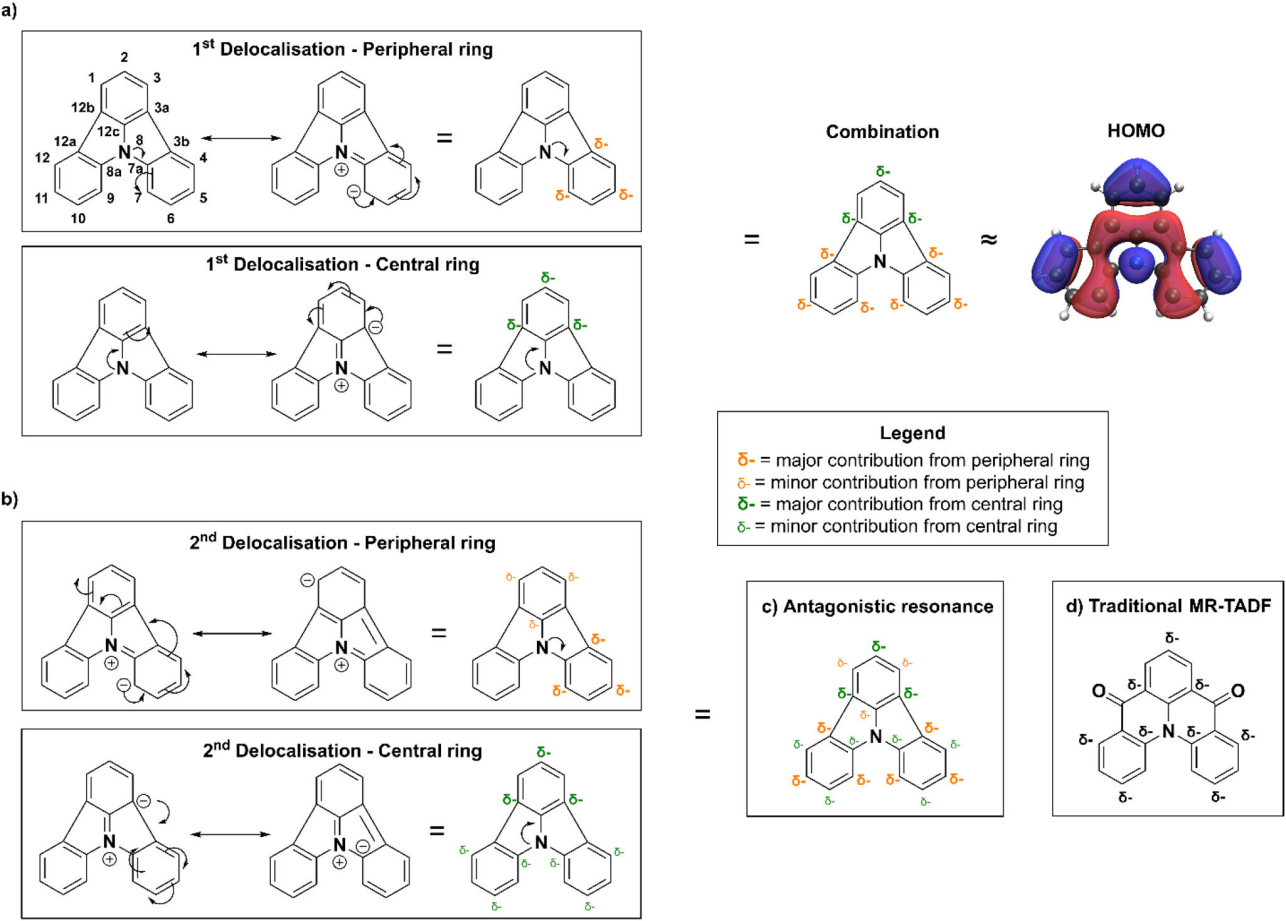
Comparison of different resonance patters of indolocarbazoles with electron donation into either the central or peripheral ring. The emitter shown in d) is **DiKTa**.^[^ ^48^ ^]^

The optoelectronic properties of the NICz family are evidently driven by the position of the N‐pyridine substitution, but the influence of this substitution is best understood in terms of the electronics of the central indole N. Considering first indolocarbzole (which lacks any embedded pyridine groups), we can examine two types of resonance structures (**Figure** **14**a). The central N can either: 1) donate electron density into the peripheral ring, placing partial negative charges on C_3b_, C_5_, and C_7_, or 2) donate electron density into the central ring, in which case the charges are placed on C_2_, C_3a_, and C_12b_. To a first approximation, the HOMO of indolocarbzole (as well as the rest of the NICz family) can be considered as a sum of these resonance forms, with adjacent partial negative charges on atoms C_3a_ and C_3b_. However, there is also additional electron density on C_12c_, C_7a_, and especially C_5_. This contribution arises from further delocalization of the negative charge onto the adjacent ring system in each resonance structure (Figure 14b). From the peripheral ring, the negative charge can be delocalized onto the central ring at positions C_1_, C_2_, and C_12c_, while from the central ring, the charge can be delocalized onto either of the two peripheral rings at the C_4_, C_6_, and C_7a_ positions. Because these second delocalization resonance structures disrupt the aromaticity of two rings, they have less contribution to the overall HOMO distribution.

Importantly, these different resonance patterns are not complimentary. Instead, the resonance contributions from the central and peripheral ring systems place partial negative charges on adjacent atoms throughout the aromatic backbone (Figure 14c). This is unlike traditional MR‐TADF designs, in which the different resonance patterns combine constructively to provide the same pattern of partial charges (Figure 14d). When the pyridine N is introduced into the indolocarbazole structure, there is no position at which the negative charge from the central N can be delocalized onto the pyridine N in both resonance structures. Positions 4 and 6 will stabilize the charge from the central ring resonance form, while positions 5 and 7 will favor the peripheral ring form. Because these effects are in competition, it becomes difficult to predict a priori which position of pyridine N will result in a more localized HOMO, and hence a smaller Δ*E*_ST_. From the results presented here, it is clear that positions 4 and 6 result in a favorable confinement, while positions 5 and 7 do not. This concurs with the prior observation by Kader *et al.* ^[^ ^28^ ^]^ that substitution of the central ring has a greater influence on the optoelectronic properties than the substitution of the peripheral rings, suggesting that the central ring resonance form is dominant. Finally, this can be simply rationalized in terms of the number of adjacent rings, as electron density donated into the central ring can be further delocalized to either of the two peripheral rings, while each peripheral ring can only donate density into the one central ring.

This unusual property is critical for the success of the NICz scaffold as a biluminescent emitter in **6NICz** and **6,10NICz**, as the pyridine substitution does not result in complete separation of the frontier molecular orbitals. Instead, an intermediate Δ*E*_ST_ value is obtained, resulting in a relatively slow *k_RISC_* and hence competitive phosphorescence. Conversely, the substitution pattern in **5NICz** and **7NICz** results in a larger Δ*E*_ST_ and thus negligible *k_RISC_*, permitting enhanced *k_Ph_*.

## 8 Conclusion

We have carried out an extensive photophysical survey of five nitrogen‐containing indolocarbazoles, supported by a theoretical study using the post‐HF RI‐SCS‐ADC(2) method. In dilute toluene solution, the five compounds are UV fluorescent emitters with very narrowband emission. The differences in the energy of emission between the five compounds are driven by changes in LUMO level as the position and number of pyridine N substitution changes, while the HOMO level remains largely insensitive to these structural changes. The greatest stabilization of the LUMO, and therefore the lowest energy of emission, is seen in **6,10NICz**, followed by **6NICz**, **4NICz**, **5NICz,** and finally **7NICz**. PMMA films doped with 0.1, 1, and 10 wt.% of the different emitters show biluminescence at room temperature, with distinct fluorescence and phosphorescence bands. Additionally, the two *meta*‐substituted emitters **6NICz** and **6,10NICz** show multi‐resonant thermally activated delayed fluorescence at 1 wt.% doping, with **6,10NICz** also exhibiting MR‐TADF at 10 wt.%. This is, to the best of our knowledge, the first unequivocal demonstration of MR‐TADF/RTP type biluminescence in an organic emitter. The RTP persists even at very low‐doping concentrations and so is monomolecular in nature. We have additionally elucidated the rate constants of all the photophysical processes of **6,10NICz** as a model emitter, and based on these we have suggested several important design criteria that we hope will guide future research into these enigmatic materials. We note that the discovery of MR‐TADF/RTP biluminescence in this family of emitters suggests a number of exciting applications for future research, including the sensing of oxygen and/or temperature, and broad‐spectrum emission.

## Conflict of Interest

The authors declare no conflict of interest.

## Supporting information



Supporting Information

## Data Availability

The research data supporting this publication can be accessed at https://doi.org/10.17630/ea27adbc‐4b32‐4563‐9ece‐7920da6b9359.
