# Supplementary material for: Simultaneous Multi‐Resonant Thermally Activated Delayed Fluorescence and Room Temperature Phosphorescence from Biluminescent Nitrogen‐Containing Indolocarbazoles
Source: Adv Sci (Weinh). 2025 May 14;12(29):e03175. doi: 10.1002/advs.202503175 (PMC12362827; doi:10.1002/advs.202503175)
Supplement: Supplementary file 1 — Supporting Information [file ADVS-12-e03175-s001.pdf]

ADVANCED SCIENCE
Open Access

Supporting Information

for *Adv. Sci.*, DOI 10.1002/advs.202503175

Simultaneous Multi-Resonant Thermally Activated Delayed Fluorescence and Room Temperature Phosphorescence from Biluminescent Nitrogen-Containing Indolocarbazoles

Oliver S. Lee, Aidan P. McKay, David B. Cordes, Stuart L. Warriner, Malte C. Gather and Eli Zysman-Colman**

Simultaneous multi-resonant thermally activated delayed fluorescence and room-temperature phosphorescence from biluminescent nitrogen containing indolocarbazoles

Supporting Information

Oliver S. Lee,[a,b] Aidan P. McKay,[a] David B. Cordes,[a] Stuart L. Warriner,[c] Malte C. Gather[b,d*] and Eli Zysman-Colman[a*]

[a]Organic Semiconductor Centre, EaStCHEM School of Chemistry, University of St Andrews, St Andrews, UK, KY16 9ST

[b]Organic Semiconductor Centre, SUPA School of Physics and Astronomy, University of St Andrews, St Andrews, UK, KY16 9SS.

[c]School of Chemistry, University of Leeds, Leeds, UK, LS2 9JT

[d]Humboldt Centre for Nano- and Biophotonics, Department of Chemistry and Biochemistry, University of Cologne, Greinstr. 4-6, 50939 Köln, Germany.

Contents

Computations **3**

Methodology **3**

General methods 3

Synthetic Overview 8

4-(2-nitrophenyl)pyridine 9

Norharmane (β-carboline) 12

9-(2-chloropyridin-3-yl)-carbazole (Chloro-4PCz) 14

Pyrido[2’,3’:4,5]pyrrolo[3,2,1-jk]carbazole (4NICz) 16

9-(3-bromopyridin-4-yl)-carbazole (Bromo-5PCz) 19

Pyrido[3’,4’:4,5]pyrrolo[3,2,1-jk]carbazole (5NICz)..22
9-(4-chloropyridin-3-yl)carbazole (Chloro-6PCz) 26
Pyrido[4’,3’:4,5]pyrrolo[3,2,1-jk]carbazole (6NICz)...28
9-(3-chloropyridin-2-yl)carbazole (Chloro-7PCz) 32
Pyrido[3’,2’:4,5]pyrrolo[3,2,1-jk]carbazole (7NICz)...34
9-(4-Chloropyridin-3-yl)-9H-pyrido[3,4-b]indole (6,10PyCb).......................................38
Pyrido[3,4- b]pyrido[4',3':4,5]pyrrolo[3,2,1-hi]indole (6,10NICz)................................40
Crystal Structures **43**
Additional Computational and Photophysical Data **46**
References **57**

Computations

All computations (except vibronic coupling and spin-orbit-coupling, see below) were performed using the spin-component scaled (SCS) version of the second-order algebraic diagrammatic construction method (ADC(2))[1–5] with the cc-pVDZ basis set,[6–8] in the gas phase. The calculations were accelerated using the resolution of the identity approximation (RI) and the cc-pVDZ auxiliary basis set.[9] All computations were conducted in the Turbomole 7.5 program.[10] The geometry of each molecule was first optimized (from structures drawn *in silico*) before the first two singlet and triplet excited states were calculated. Difference density plots were obtained by taking the difference of the total electron density in the excited state (S_1 or T_1) from the ground state. Vibronic coupling was calculated using the PBE0[11–13] functional with GD3BJ[14,15] empirical dispersion and the 6-31G** basis set.[16–18] The ground-state geometry was reoptimized at this level before vibrational frequencies were calculated. The S_1 geometry and vibrational frequencies were calculated using time-dependent DFT (TD-DFT)[19,20] using the Tamm-Dancoff approximation (TDA-DFT),[21] solving for the 5 lowest energy singlet states. The vibrationally resolved emission spectrum ($S_1 \rightarrow S_0$) was then calculated using the full Franck-Condon-Herzberg-Teller method (FCHT)[22] at 298.15 K. The final simulated spectrum was constructed using a sum of Gaussian functions with full-width at half-maxima of 400 cm^{-1}. SOC was calculated at the DFT ground state geometry using PySOC.[23] All computations were managed by the Digichem software suite V6.1[24] which incorporates a number of open-source libraries, including cclib[25] for file parsing and Openbabel/pybel[26,27] for file conversion. 3D density plots were rendered using VMD 1.9.3[28] and tachyon.[29] 2D graphs were plotted with matplotlib.[30]

Methodology

General methods

General Synthetic Procedures. All reagents and solvents were obtained from commercial sources. Carbazole and N- bromosuccinimide (NBS) were purified before use by recrystallization from boiling DCM and water respectively, all other reagents were used as received. Air-sensitive reactions were performed under a nitrogen atmosphere using Schlenk techniques. Flash column chromatography was carried out using silica gel (Silica-P from Silicycle, 60 Å, 40-63 µm). Analytical thin-layer-chromatography (TLC) was performed with silica plates with aluminum backings (250 µm with F-254 indicator). TLC visualization was accomplished by 254/365 nm UV lamp. NMR spectra were recorded on a Bruker Advance spectrometer (400 MHz for ^{1}H and 101 MHz for ^{13}C). The following abbreviations have been used for multiplicity assignments: “s” for singlet, “d” for doublet, “dd” for

doublet of doublets, "dt" for doublet of triplets, "ddd" for doublet of doublets of doublets, "t" for triplet, "td" for triplet of doublets, and "m" for multiplet. ^{1}H and ^{13}C NMR spectra were referenced to residual solvent peaks ($CDCl_3$ or d_6-DMSO) with respect to TMS (δ = 0 ppm). High performance liquid chromatography (HPLC) was conducted on a Shimadzu LC-40 HPLC system. HPLC traces were performed using a Shim-pack GIST 3 μm C18 reverse phase analytical column using acetonitrile:water as the mobile phase. Melting points were measured using open-ended capillaries on an Electrothermal 1101D Mel-Temp apparatus and are uncorrected. Electrospray ionization technique (ESI) High-resolution mass spectrometry (HRMS) was performed at the School of chemistry at the University of Leeds.

Photophysical measurements. Optically dilute solutions of concentrations on the order of 10^{-5} to 10^{-6} M were prepared in spectroscopic or HPLC grade solvents for absorption and emission analyses. Absorption spectra were recorded at room temperature on a Shimadzu UV-2600 double beam spectrophotometer with a 1 cm quartz cuvette. Molar absorptivity determination was verified by linear regression analysis of values obtained from at least five independent solutions at varying concentrations ranging from 10^{-5} M to 10^{-6} M. Steady-state emission spectra and time-resolved emission decays were recorded at 298 K using an Edinburgh Instruments FS5 spectrofluorometer, unless a temperature is otherwise indicated in the experiment. Time-resolved spectra were recorded by time-correlated single photon counting (TCSPC) using a 375 nm pulsed laser diode (Picoquant, model PLS 370), or by the multi-channel scaling (MCS) technique with a microsecond flash lamp. For photoluminescence quantum yield measurements, degassed solutions were prepared via three freeze-pump-thaw cycles and spectra were taken using home-made Schlenk quartz cuvette. Photoluminescence quantum yields for solutions were determined using the optically dilute method[31]. The Beer-Lambert law was found to remain linear at all concentrations. For each sample, linearity between absorption and emission intensity was verified through linear regression analysis with the Pearson regression factor (R^2) for the linear fit of the data set surpassing 0.9. Individual relative quantum yield values were calculated for each solution and the values reported represent the slope obtained from the linear fit of these results. The quantum yield of the sample, Φ_{PL}, can be determined by the equation $\Phi_{PL} = (\Phi_r * \frac{A_r}{A_s} * \frac{I_s}{I_r} * \frac{n_s^2}{n_r^2})^2$, where A stands for the absorbance at the excitation wavelength, I is the integrated area under the corrected emission curve and n is the refractive index of the solvent with the subscripts "s" and "r" representing sample and reference respectively. Φ_r is the absolute quantum yield of the external reference quinine sulfate (Φ_r = 54.6% in 1 M H_2SO_4),[32] The experimental uncertainty in the emission quantum yields is conservatively estimated to be 10%, though we have found that statistically we can reproduce Φ_{PL} values to 3%

relative error. Evaporated thin films were used to measure thin film photophysical properties in the solid state. An integrating sphere was employed for photoluminescence quantum yield measurements for thin film samples. Time-resolved PL measurements of the thin films were carried out using the multi-channel scaling (MCS) technique and time correlated single photon counting system (TCSPC) and were kept in a vacuum of < 8 × 10^{-4} mbar. Prompt fluorescence and phosphorescence spectra were obtained from evaporated films at 77 K and the singlet-triplet splitting energy values, ΔE_{ST}, were estimated from the difference in the onsets of the corresponding spectra. For ΔE_{ST} measurements film samples were loaded inside a cold finger cryostat (Oxford Instruments) and placed under vacuum, with temperature control from 300 K – 77 K. Phosphorescence spectra were measured 1 ms after the excitation by the flashlamp with an integration time of 9 ms.

Fitting of time-resolved luminescence measurements: Time-resolved PL measurements were fitted to a sum of exponentials decay model, with chi-squared (χ^2) values between 1 and 2, using the Fluoracle software program. Each component of the decay is assigned a weight, (w_i), which is the contribution of the emission from each component to the total emission.

The average lifetime was then calculated using the following:

Two exponential decay model:

$$\tau_{AVG} = \tau_1 w_1 + \tau_2 w_2$$

with weights defined as $w_1 = \frac{A1\tau_1}{A1\tau_1 + A2\tau_2}$ and $w_2 = \frac{A2\tau_2}{A1\tau_1 + A2\tau_2}$ where A1 and A2 are the preexponential-factors of each component.

Three exponential decay model:

$$\tau_{AVG} = \tau_1 w_1 + \tau_2 w_2 + \tau_3 w_3$$

with weights defined as $w_1 = \frac{A1\tau_1}{A1\tau_1 + A2\tau_2 + A3\tau_3}$, $w_2 = \frac{A2\tau_2}{A1\tau_1 + A2\tau_2 + A3\tau_3}$ and $w_3 = \frac{A3\tau_3}{A1\tau_1 + A2\tau_2 + A3\tau_3}$ where A1, A2 and A3 are the preexponential-factors of each component.

Fitting of the spectral profiles and kinetic evaluation: The experimental PL of **6,10NICz** as a 10wt% dopant in PMMA was fitted using a total of four Gaussian functions. Each Gaussian was first roughly fitted to the PL 'by hand'. These parameters were then used as an initial guess to optimise the fitting using a non-linear least squares function provided by the SciPy library[33] (the `curve_fit()` function) using the following script:

```
from scipy.optimize import curve_fit
import numpy as np
import math
```

```python
# Data.csv is a comma-separated value file of the PL (x in first column, y in second).
x, y = np.loadtxt('data.csv', delimiter=',', unpack = True)

def gaussian(x, y0, xc, A, w):
    constant = 4 * math.log(2)
    t1 = (-constant * (x - xc)**2)/(w**2)
    t2 = w * (math.pi / constant)** 0.5
    return y0 + ( (A * math.exp(t1)) / t2 )

def func(x, *params):
    y = np.zeros_like(x)
    for i in range(0, len(params), 4):
        y0 = params[i]
        xc = params[i+1]
        A = params[i+2]
        w = params[i+3]
        y = [y[i] + gaussian(x_val, y0, xc, A, w) for i, x_val in enumerate(x)]
    return y

# Initial guess.
# Parameters are:
# 1) y-intercept (y0)
# 2) x mid-point (xc)
# 3) area (A)
# 4) full-width at half maximum (w)
guess = [
    0,     3.21, 0.068, 0.09,
    0,     3.12, 0.035, 0.10,
    0,     3.02, 0.036, 0.12,
    0,     2.55, 0.051, 0.62
]

popt, pcov, infodict, mesg, ier = curve_fit(func, x, y, p0=guess, maxfev = 100000, full_output = True, bounds
= (0, math.inf))
print(popt)
```

For ease of use, the Gaussian function was implemented with area as an input parameter directly, rather than the more accustomed peak height, according to the following equation:

$$y = y_0 + \frac{A \cdot exp(\frac{-4\ln{(2)}(x - x_c)^2}{w^2})}{w\sqrt{\frac{\pi}{4\ln{(2)}}}}$$

The optimization was performed within the bounds 0 → +inf for each parameter. The optimised parameters were as follows:

No.	y_0	x_c	A	w
Fl_1	1.26530817e-11	3.19107074e+00	6.58382110e-02	1.16638626e-01

Fl_2	1.26530817e-11	3.08742525e+00	1.07272816e-01	2.01249571e-01
Fl_3	1.26530817e-11	2.98137579e+00	1.42717219e-01	3.19587713e-01
Ph	1.26530817e-11	2.46773815e+00	3.71117052e-01	7.33046487e-01

The kinetic parameters were then estimated according to the numerical methodology proposed by Tsuchiya *et al.*,[34] using a modified version of the calculator provided in the supporting information of that manuscript.

Synthetic Overview

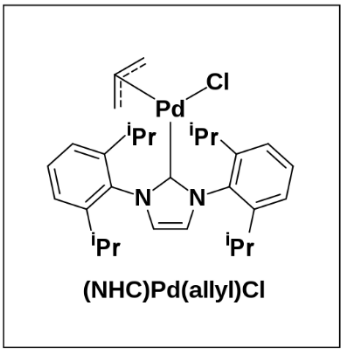

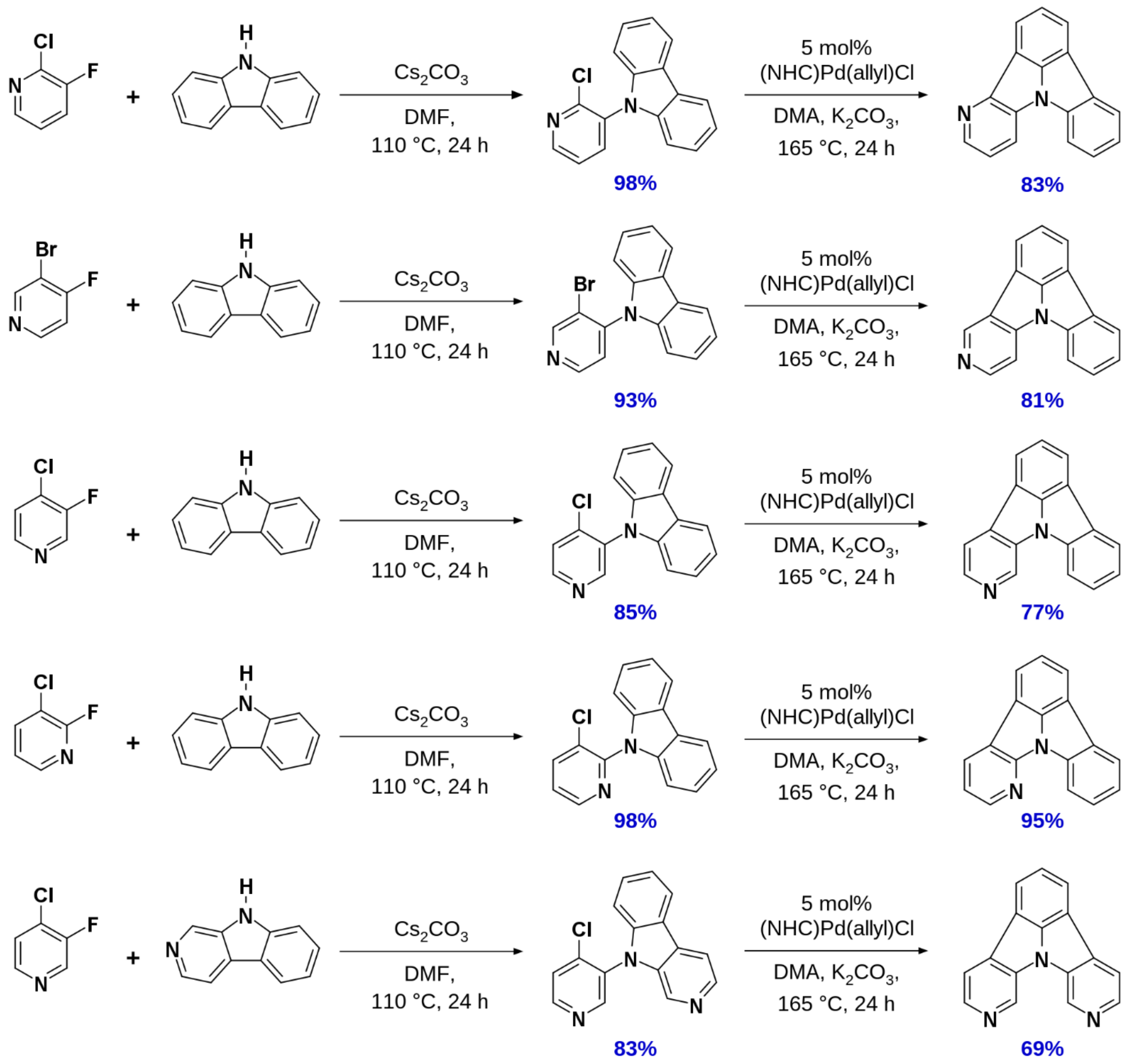

Figure **S1**. Synthetic scheme for **4NICz**, **5NICz**, **6NICz**, **7NICz** and **6,10NICz**. DMF: dimethylformamide, DMA: dimethylacetamide. Percentages are isolated yields for each step.

Each emitter was synthesized in a two-step scheme following the protocol of Kader *et al.*[35] (Figure **S1**) Firstly, a nucleophilic aromatic substitution reaction (S_NAr) between an appropriately dihalogenated pyridine and either carbazole (**4NICz**, **5NICz**, **6NICz**, **7NICz**) or norharmane (**6,10NICz**) was performed, using Cs_2CO_3 as the catalyst. Here, we found that higher temperatures lead to a decrease in yield. This we attribute to activation of substitution at the chlorine over the fluorine, evidenced by the appearance of a side-product with a molecular mass corresponding to the chlorine-substituted structure in the gas-chromatography mass-spectrometry (GCMS) of the crude reaction mixture. We found 110 °C to be a good compromise between reaction speed and yield, leading to good to excellent yields of 83 to 99%. This step was followed by a Pd-catalysed cyclisation reaction. In the case of **6,10NICz** the yield was somewhat depressed to 69%, but this is likely due to imperfect selectivity towards the desired **6,10NICz** isomer.[35] The yields of the remaining emitters range from good (77%) to excellent (95%). We also note that the high catalyst loading of 5 mol% was likely excessive, and in the case of **5NICz** and **6NICz** at least, a loading of 2.5 mol% produced similar yields (67 and 77% respectively). Finally, the norharmane required for **6,10NICz** was synthesized from a two-step scheme (Figure **S2**). This consisted of a Suzuki-Miyaura coupling between 2-chloronitrobenzene and 4-pyridinylboronic acid, followed by a Cadogan reductive cyclization reaction of the nitro intermediate. The reactions proceeded with excellent and good yields respectively, with a combined yield for the two steps of 60%.

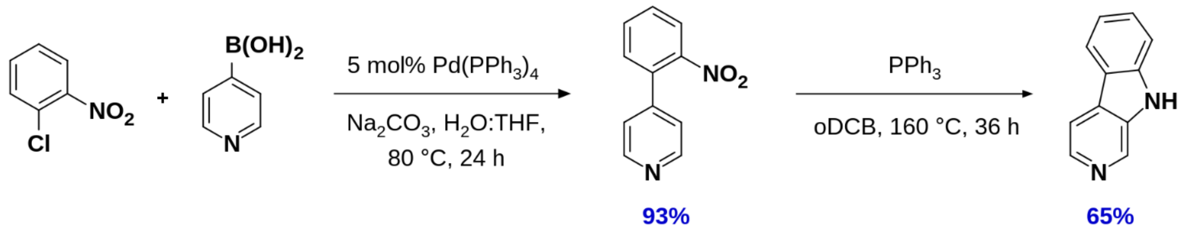

Figure **S2**. Synthetic scheme for β-carboline (norharmane). THF: tetrahydrofuran. oDCB: ortho-dichlorobenzene. Percentages are isolated yields for each step.

4-(2-nitrophenyl)pyridine

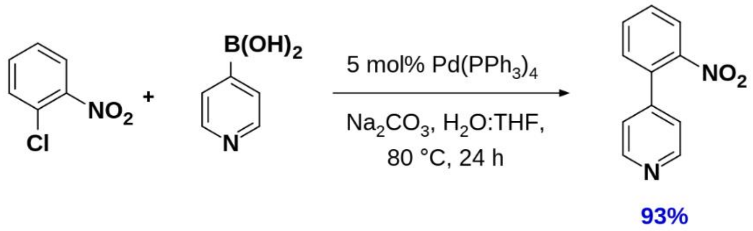

Adapted from reference [36]. 1-chloro-2-nitrobenzene (1.00 g, 6.35 mmol, 1.0 equiv.), 4-pyridinylboronic acid (1.95 g, 15.9 mmol, 2.5 equiv.) and Na_2CO_3 (4.04 g, 38.08 mmol, 6.0 equiv.) were combined and degassed by three successive vacuum/nitrogen cycles. H_2O (20.0 mL) and THF (20.0 mL) were then added successively by syringe, before the solvents were degassed by nitrogen bubbling for 10 min. Under a flow of N_2, tetrakis(triphenylphosphine)palladium(0) (183 mg, 0.16 mmol, 2.5 mol%) was added to form a bright yellow solution. The reaction mixture was then heated to 80 °C for *ca.* 48 h, after which partial conversion was observed *via* GCMS. A further portion of tetrakis(triphenylphosphine)palladium(0) (152 mg, 0.13 mmol, 2.5 mol%) was added and the mixture was heated at 80 °C overnight, after which it had turned blood red. The reaction as allowed to cool to room temperature before being poured into brine. A liquid-liquid extraction was then performed with EtOAc/brine and the organic fractions were combined and dried over $MgSO_4$, forming an orange-brown solution from which a black precipitate slowly formed. The crude was filtered through celite with EtOAc and evaporated directly onto silica. The crude was purified by flash column chromatography in 50% EtOAc/Hexane. The fractions were combined and the solvent was removed to **yield** the product as a pale yellow oil (1.19 g, 93%). **^{1}H NMR (400 MHz; $(CD_3)_2SO$) δ_H (ppm)**: 8.66 (dd, *J* = 4.5, 1.5 Hz, 2H), 8.11 (dd, *J* = 8.1, 1.3 Hz, 1H), 7.84 (ddd, *J* = 7.6, 1.3, 0.7 Hz, 1H), 7.73 (ddd, *J* = 7.6, 1.5, 0.8 Hz, 1H), 7.60 (dd, *J* = 7.8, 1.5 Hz, 1H), and 7.40 (dd, *J* = 4.2, 1.6 Hz, 2H). **^{13}C{^{1}H} NMR (101 MHz; $(CD_3)_2SO$) δ_C (ppm)**: 150.3 (CH), 148.6 (C_{quat}), 145.6 (C_{quat}), 134.0 (CH), 133.5 (C_{quat}), 132.1 (CH), 130.5 (CH), 125.1 (CH), and 123.3 (CH). Characterization data matches those in the literature.[37]

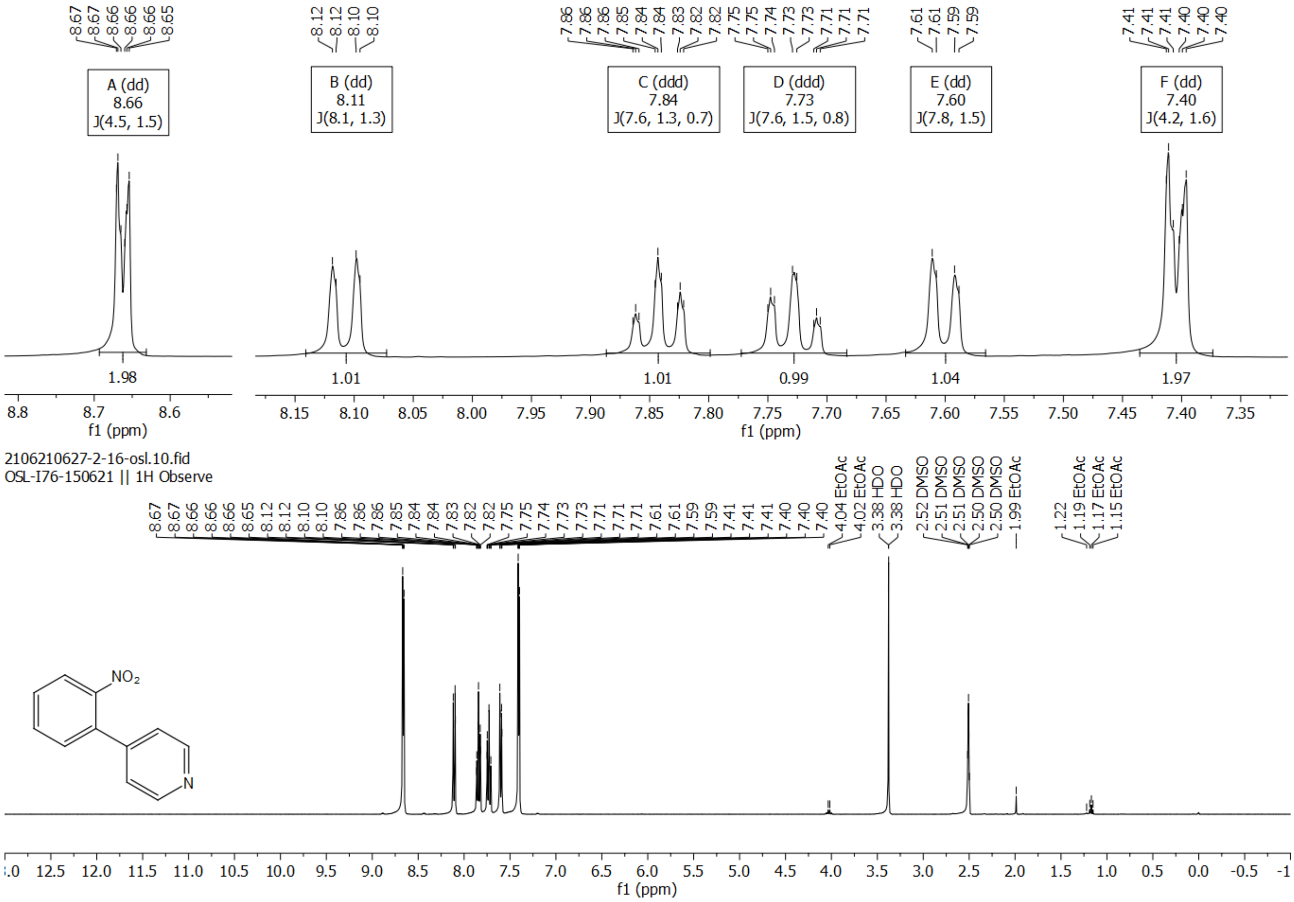

Figure **S3**. ^{1}H NMR of **4-(2-nitrophenyl)pyridine** in d_6-DMSO.

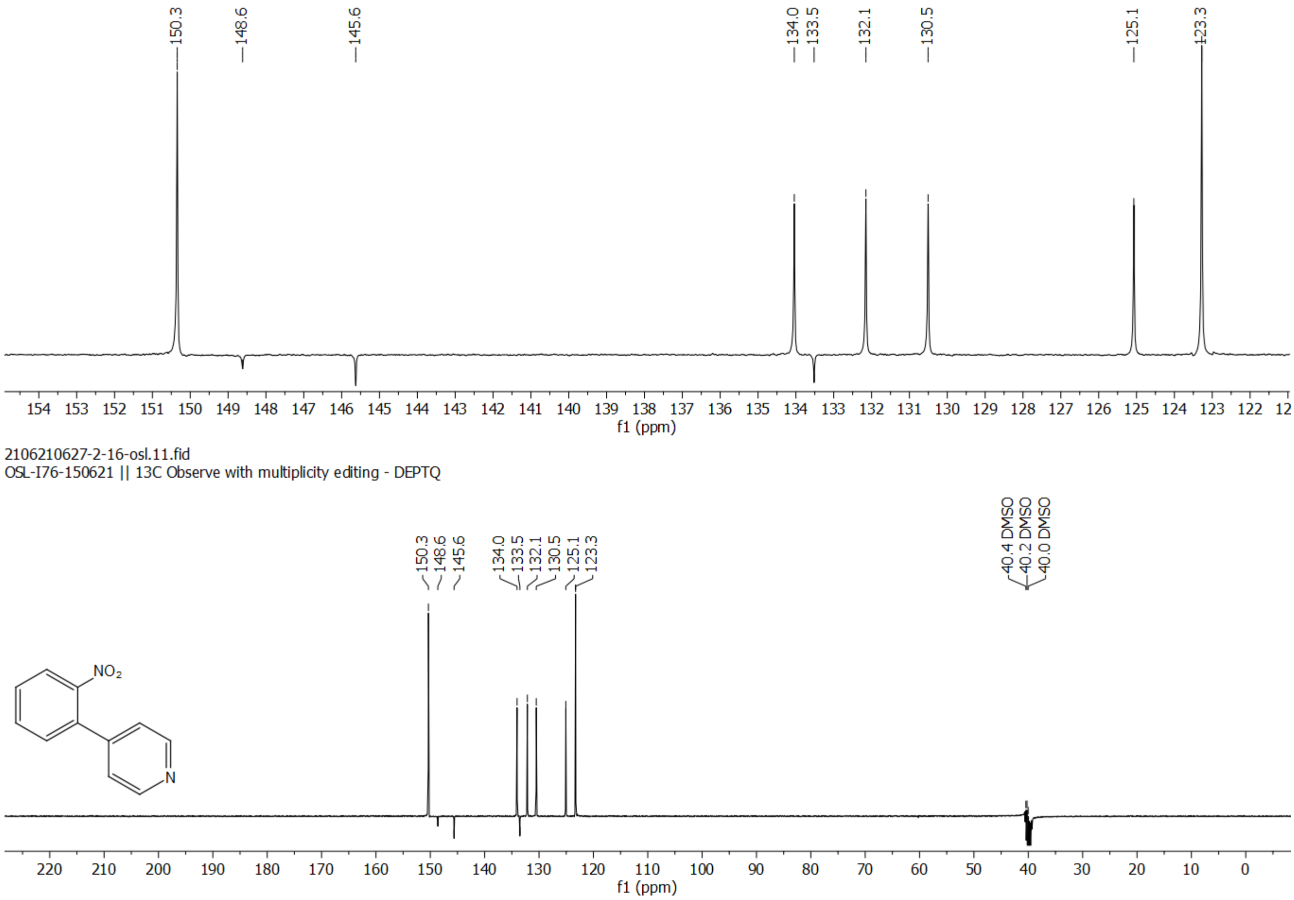

Figure **S4**. ^{13}C NMR of **4-(2-nitrophenyl)pyridine** in d_6-DMSO.

Norharmane (β-carboline)

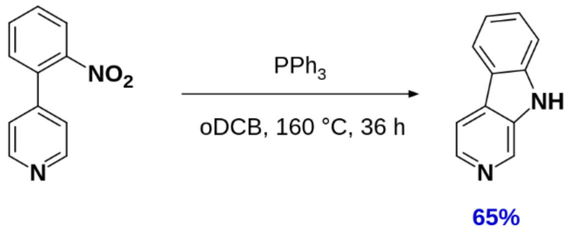

Adapted from reference [36]. 4-(2-nitrophenyl)pyridine (1.19 g, 5.93 mmol, 1.0 equiv.) was dissolved in EtOAc, washed into a reaction vessel, and the solvent was evaporated. PPh_3 (4.67 g, 17.80 mmol, 3.0 equiv.) was added and the rection was degassed by three successive vacuum/nitrogen cycles. Under a flow of N_2, dry 1,2-dichlorobenzene (oDCB, 60.0 mL) was added *via* syringe to form a clear, orange solution. The reaction mixture was heated to 160 °C until conversion of the starting material reached *ca.* 95% by GCMS (*ca.* 36 h). The reaction was allowed to cool to room temperature before the solvent was evaporated. A liquid-liquid extraction was then performed with EtOAc/brine and the organic fractions were combined and dried over Na_2SO_4. The crude was then dissolved in EtOAc, wet-loaded onto a silica column, and purified by flash column chromatography starting at 40% EtOAc/Hexane. After oDCB was removed, the polarity was increased to 45% EtOAc. Once the first spot had been removed, the polarity was increased further to 50%. Once the product began to be collected, the polarity was increased to 55%, and later to 60% once the majority of the product has been obtained. The fractions were combined and evaporated to yield a white powder (0.93 g). The crude was then recrystallised from *ca*. 30% EtOAc/hexane (*ca.* 500 mL) to yield small white crystals (0.44 g.) The filtrate was collected, evaporated, and recrystallized from the same solvent system for a second (0.20 g) and third (0.02 g) crop, for a combined **yield** of 0.65 g (65 %). **R_f**: 0.20 (90 : 10 EtOAc : hexane). **Mp**: 199 – 200 °C (lit:[38] 200 – 201°C). **^{1}H NMR (400 MHz; $(CD_3)_2SO$) δ_H (ppm)**: 11.62 (s, 1H), 8.90 (d, *J* = 1.1 Hz, 1H), 8.34 (d, *J* = 5.2 Hz, 1H), 8.25 (dt, *J* = 7.9, 1.0 Hz, 1H), 8.11 (dd, *J* = 5.2, 1.1 Hz, 1H), 7.60 (dt, *J* = 8.2, 1.0 Hz, 1H), 7.55 (ddd, *J* = 8.2, 6.9, 1.2 Hz, 1H), and 7.25 (ddd, *J* = 8.0, 6.9, 1.2 Hz, 1H). **^{13}C{^{1}H} NMR (101 MHz; $(CD_3)_2SO$) δ_C (ppm)**: 141.0 (C_{quat}), 138.6 (CH), 136.4 (C_{quat}), 134.5 (CH), 128.6 (CH), 127.9 (C_{quat}), 122.3 (CH), 121.1 (C_{quat}), 119.7 (CH), 115.1 (CH), and 112.4 (CH). Characterization data matches those in the literature.[39,40]

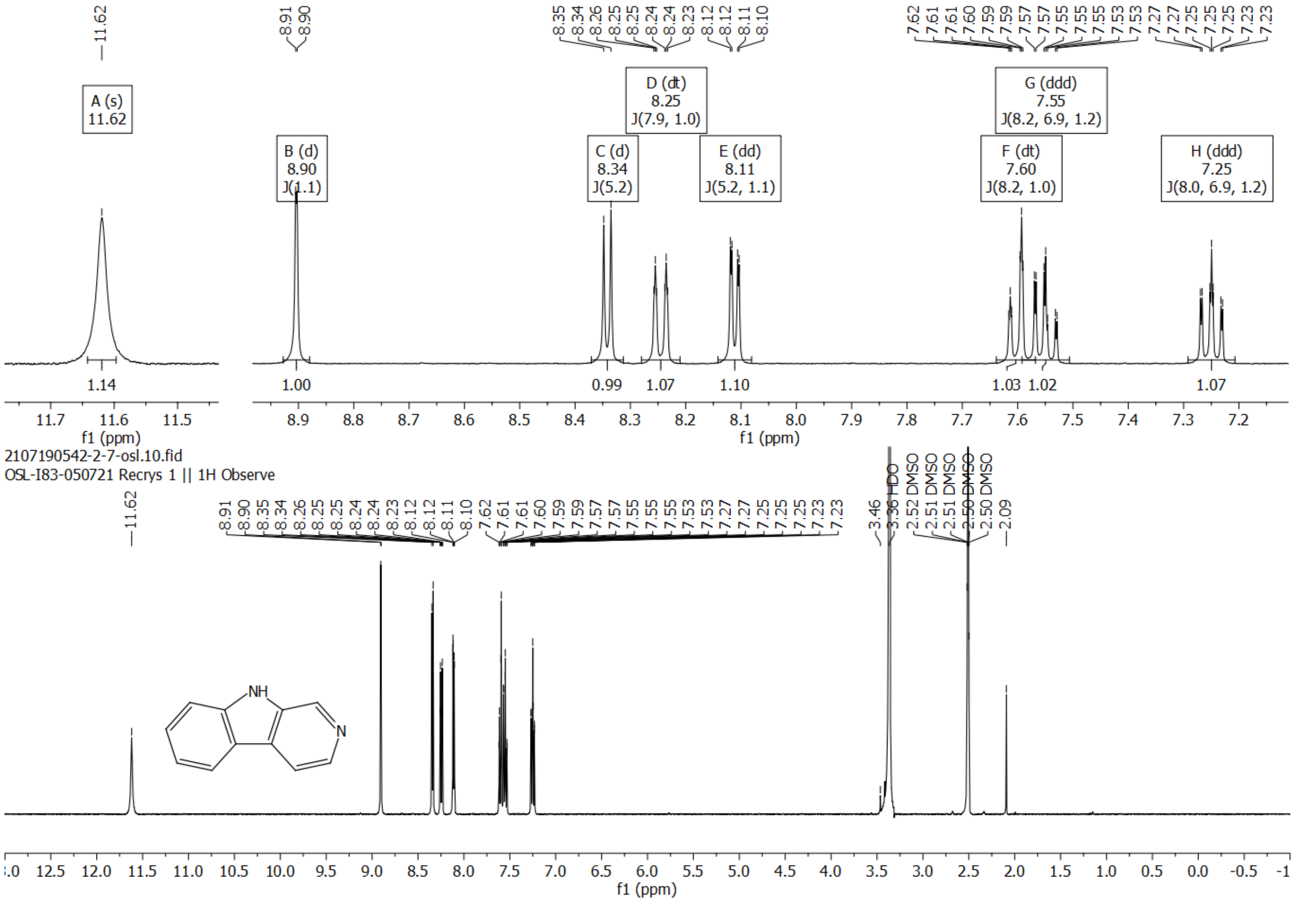

Figure **S5**. ^{1}H NMR of **norharmane** in d$_6$-DMSO.

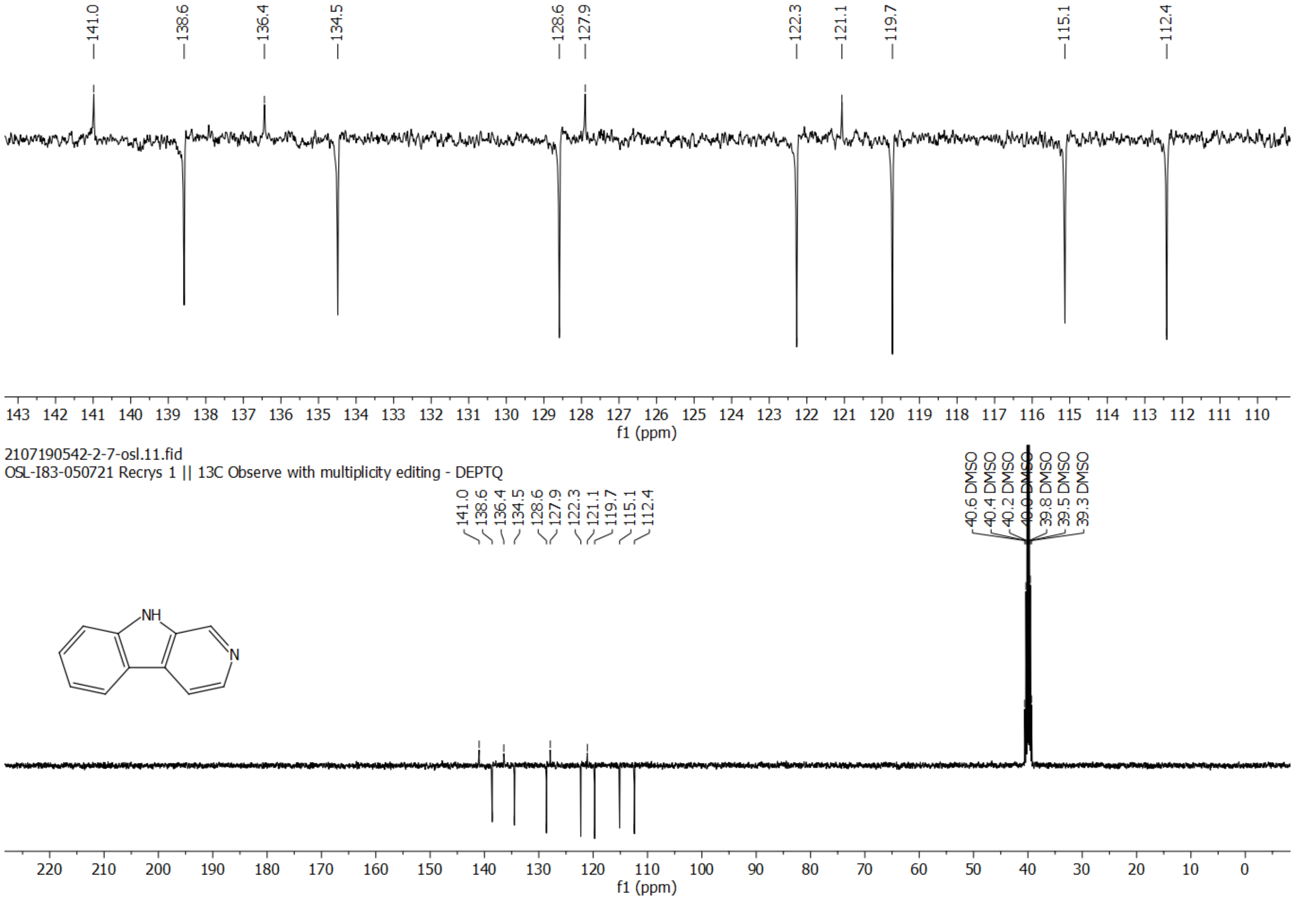

Figure **S6**. ^{13}C NMR of **norharmane** in d$_6$-DMSO.

9-(2-chloropyridin-3-yl)-carbazole (Chloro-4PCz)

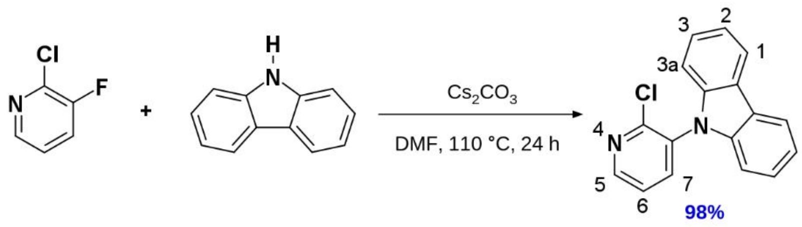

Adapted from reference [35]. Carbazole (0.50 g, 3.00 mmol, 1.0 equiv.) and Cs_2CO_3 (1.95 g, 6.00 mmol, 2.0 equiv.) were combined and degassed by three successive vacuum/nitrogen cycles. Under a flow of N_2, 2-chloro-3-fluoropyridine (0.60 mL, 6.0 mmol, 2.0 equiv.) and then DMF (6.0 mL) were added *via* syringe, forming a pale-yellow solution. The reaction mixture was then heated to 110 °C for approximately 24 h, after which a white precipitate had formed. After cooling to room-temperature, the reaction crude was poured into water, a liquid-liquid extraction was performed with EtOAc/brine, and the organic fractions were combined and dried over $MgSO_4$. The solvent was next evaporated to yield a yellow oil, before being purified by flash column chromatography in 2% EtOAc/Hexane. The oil was washed onto the column with a small amount of EtOAc. After the first and second spots were removed from the column, the polarity was increased to 5 and 8% EtOAc respectively. The product was obtained as the third spot. The solvent was removed to **yield** a yellow/white powder (0.83 g, 98+%), which was used without further purification. **^{1}H NMR (400 MHz; $CDCl_3$) δ_H (ppm)**: 8.64 (dd, *J* = 4.7, 1.8 Hz, 1H, H_5), 8.18 (ddd, *J* = 7.8, 1.4, 0.7 Hz, 2H, H_1), 7.92 (dd, *J* = 7.7, 1.8 Hz, 1H, H_7), 7.54 (dd, *J* = 7.7, 4.7 Hz, 1H, H_6), 7.45 (ddd, *J* = 8.2, 7.2, 1.3 Hz, 2H, H_3), 7.40 – 7.31 (ddd, *J* = 7.8, 7.2, 0.9 Hz, 2H, H_2), and 7.10 (ddd, *J* = 8.2, 0.9, 0.7 Hz, 2H, H_{3a}). **^{13}C{^{1}H} NMR (101 MHz; $CDCl_3$) δ_C (ppm)**: 149.5 (CH), 140.5 (C_{quat}), 139.6 (CH) 126.2 (CH), 123.7 (C_{quat}), 123.5 (CH), 120.6 (CH), 120.6 (CH), 109.8 (CH). Characterization data matches those in the literature,[35] except for two quaternary carbons expected at *ca*. 151.1 and 132.4 ppm, which could not be distinguished from the baseline.

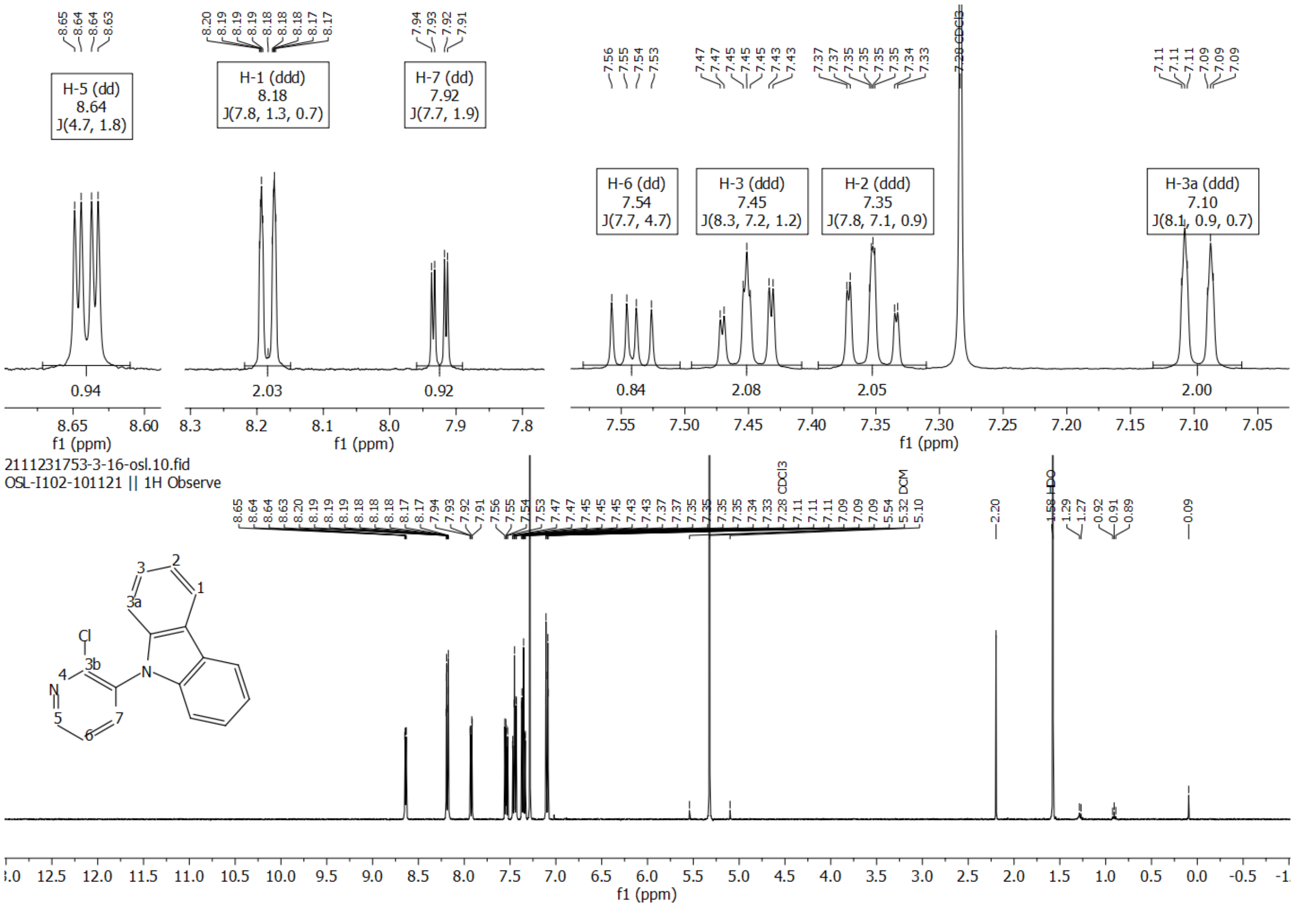

Figure **S7**. ^{1}H NMR of **Chloro-4PCz** in $CDCl_3$.

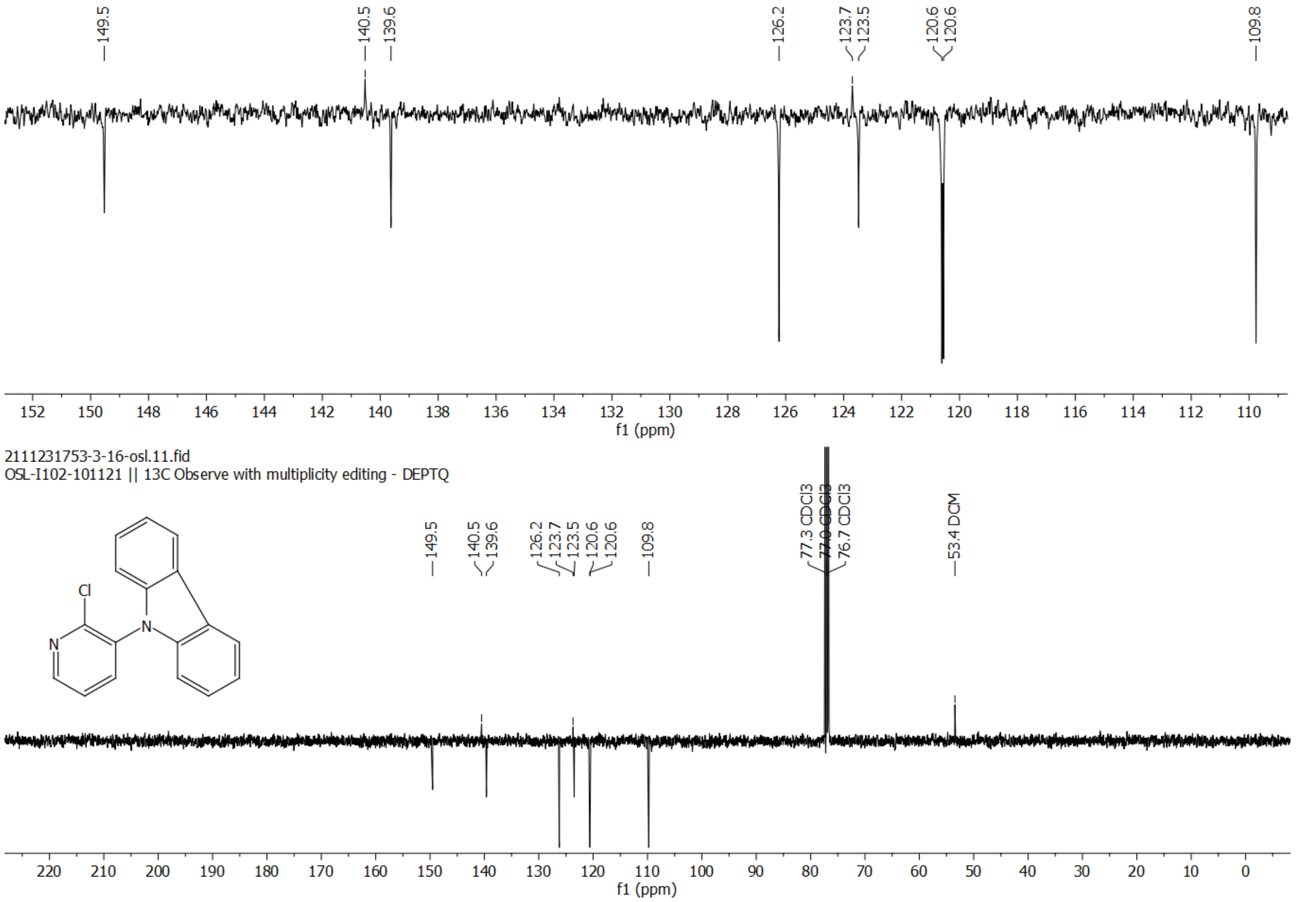

Figure **S8**. ^{13}C NMR of **Chloro-4PCz** in $CDCl_3$.

Pyrido[2',3':4,5]pyrrolo[3,2,1-jk]carbazole (4NICz)

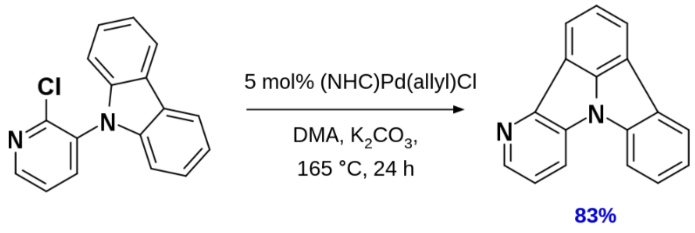

Adapted from reference [35]. 9-(2-chloropyridin-3-yl)-carbazole (0.81 g, 2.92 mmol, 1.0 equiv.), allyl[1,3-bis(2,6-diisopropyl-phenyl)imidazol-2-ylidene]chloropalladium(II) (83 mg, 0.15 mmol, 5 mol%), and K_2CO_3 (0.81 g, 5.84 mmol, 2.0 equiv.) were combined and degassed by three successive vacuum/nitrogen cycles. Under a flow of N_2, dimethylacetamide (DMA, 30.0 mL) was added *via* syringe to form a pale-yellow solution with a white suspension. The reaction mixture was then heated to 165 °C for approximately 24 h. After cooling to room-temperature, the reaction crude was filtered through celite with EtOAc to remove excess Pd, and the solvent was removed to form a brown solid. A liquid-liquid extraction was then performed with EtOAc/H_2O and the organic fractions were combined and dried over Na_2SO_4. The crude was next evaporated onto silica and purified by flash column chromatography in 70% EtOAc/Hexane to yield the crude product as a yellow-white solid after removing the solvent (0.65 g). The crude product was then recrystallised from the minimum amount of boiling 40% EtOAc/Hexane to yield small, white-brown crystals (0.48 g). The filtrate was evaporated and recrystallised in 30% EtOAc/Hexane to yield a second crop of crystals (0.11 g), for a total **yield** of 0.59 g (83%). **R_f**: 0.26 (30 : 70 EtOAc : hexane). **Mp**: 115 – 116 °C. Purity by **HPLC**: > 99.9%. **^{1}H NMR (400 MHz; $(CD_3)_2SO$) δ_H (ppm)**: 8.73 (dd, *J* = 8.3, 1.4 Hz, 1H), 8.62 (dd, *J* = 4.8, 1.4, 1H), 8.37 – 8.30 (m, 3H), 8.26 (dd, *J* = 7.5, 0.5 Hz, 1H), 7.73 (t, *J* = 7.4 Hz, 1H), 7.66 (ddd, *J* = 8.5, 7.3, 1.3 Hz, 1H), 7.62 (dd, *J* = 8.2, 4.8 Hz, 1H), and 7.46 (td, *J* = 7.6, 1.0 Hz, 1H). **^{13}C{^{1}H} NMR (101 MHz; $(CD_3)_2SO$) δ_C (ppm)**: 147.5 (C_{quat}), 143.7 (CH), 143.4 (C_{quat}), 138.7 (C_{quat}), 133.0 (C_{quat}), 129.1 (C_{quat}), 128.1 (CH), 124.4, (CH), 124.0 (CH), 123.2 (CH), 122.2 (CH), 121.6 (CH), 120.5 (CH), 120.3 (CH), 119.2 (C_{quat}), 117.5 (C_{quat}), and 113.6 (CH). **HR-MS[M+H]$^+$**: Calculated: ($C_{17}H_{11}N_2$): 243.0917; Found: 243.0919. Characterization data matches those in the literature.[35]

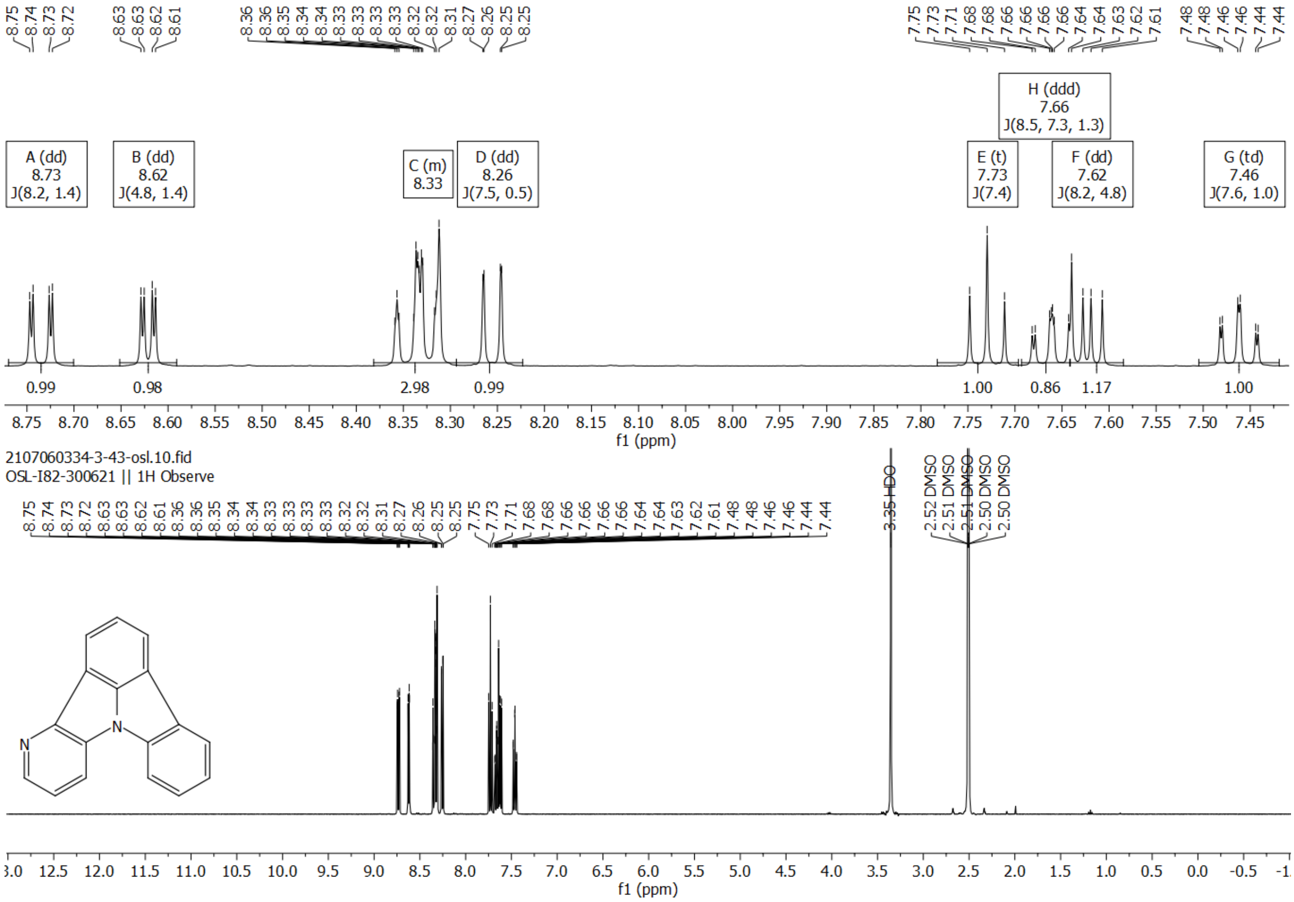

Figure **S9**. ^{1}H NMR of **4NICz** in d$_6$-DMSO.

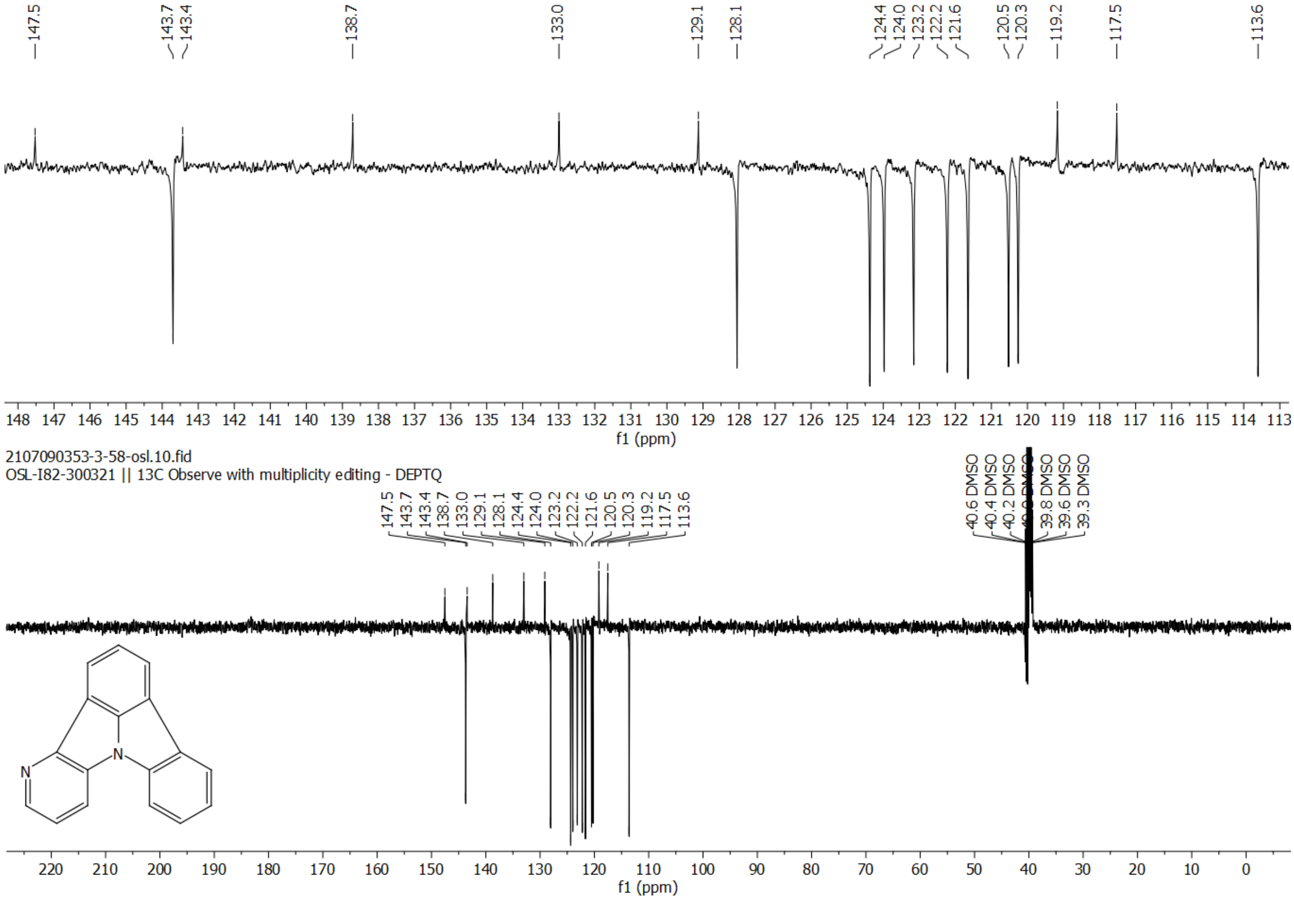

Figure **S10**. ^{13}C NMR of **4NICz** in d$_6$-DMSO.

<Sample Information>

Sample Name	: I82		
Sample ID	:		
Method Filename	: 50% Acetonitrile 50 Water 20 mins.lcm		
Batch Filename	: Re run 3 on 01092021.lcb		
Vial #	: 2-8	Sample Type	: Unknown
Injection Volume	: 5 uL		
Date Acquired	: 01/09/2021 16:44:50	Acquired by	: System Administrator
Date Processed	: 01/09/2021 17:04:52	Processed by	: System Administrator

<Chromatogram>

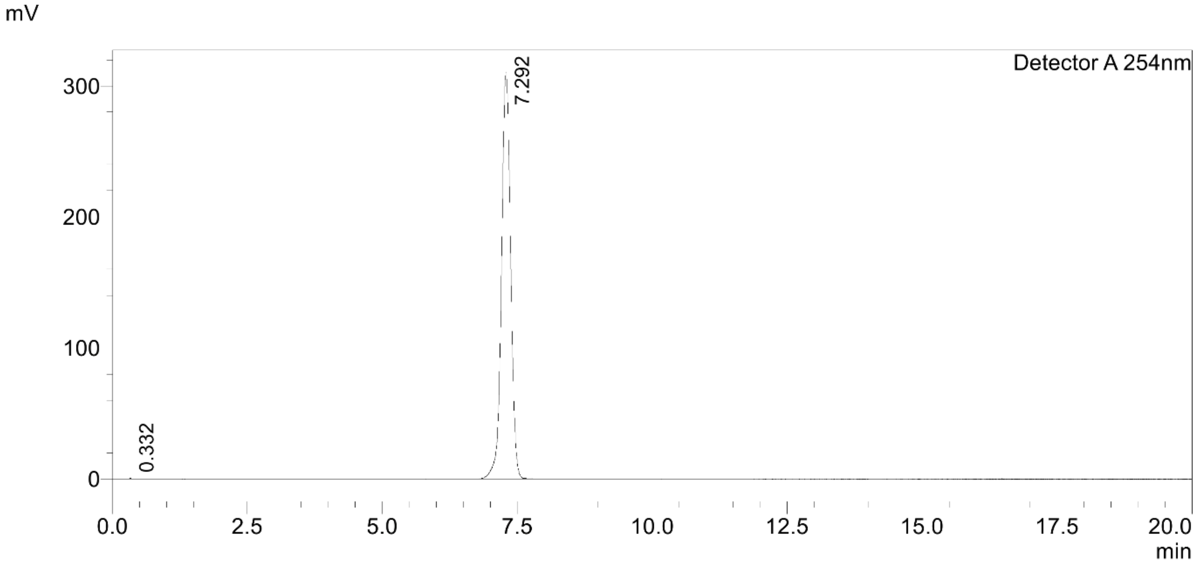

<Peak Table>

Detector A 254nm

Peak#	Ret. Time	Area	Height	Area%	Area/Height	Width at 5% Height
1	0.332	1460	724	0.040	2.016	0.068
2	7.292	3675417	309401	99.960	11.879	0.409
Total		3676877	310125	100.000		

Figure **S11**. HPLC trace of **4NICz**.

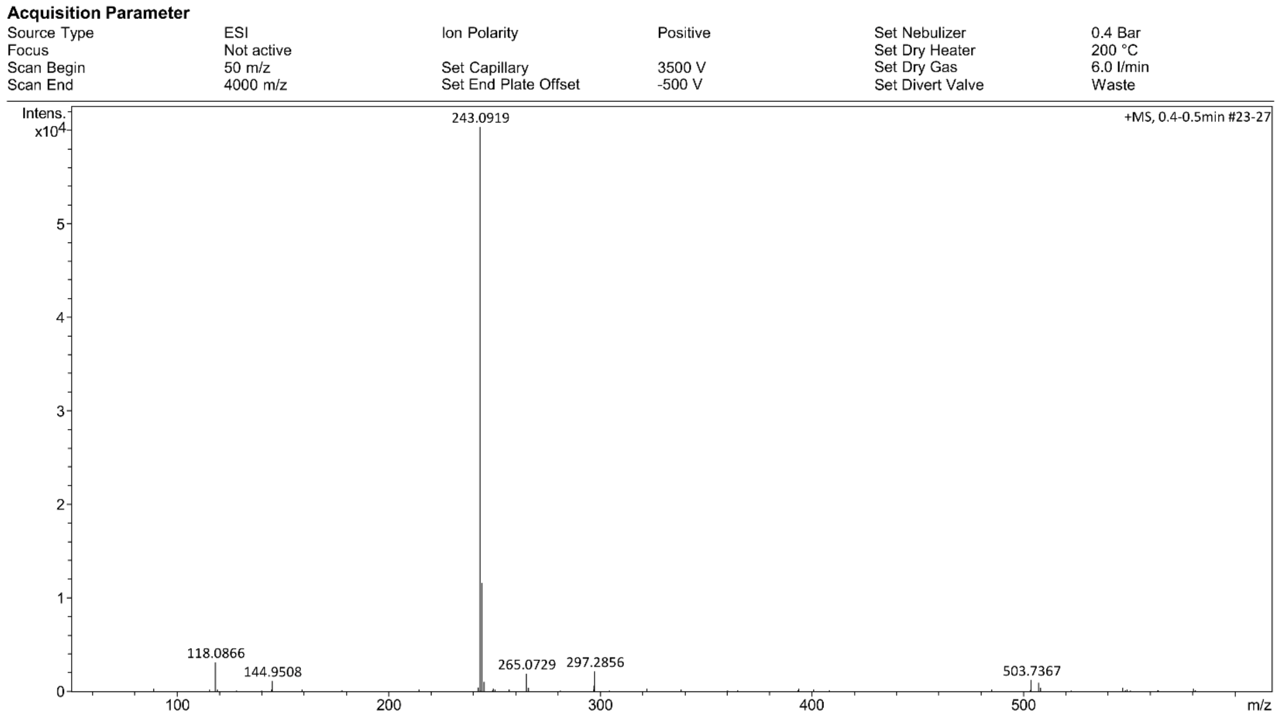

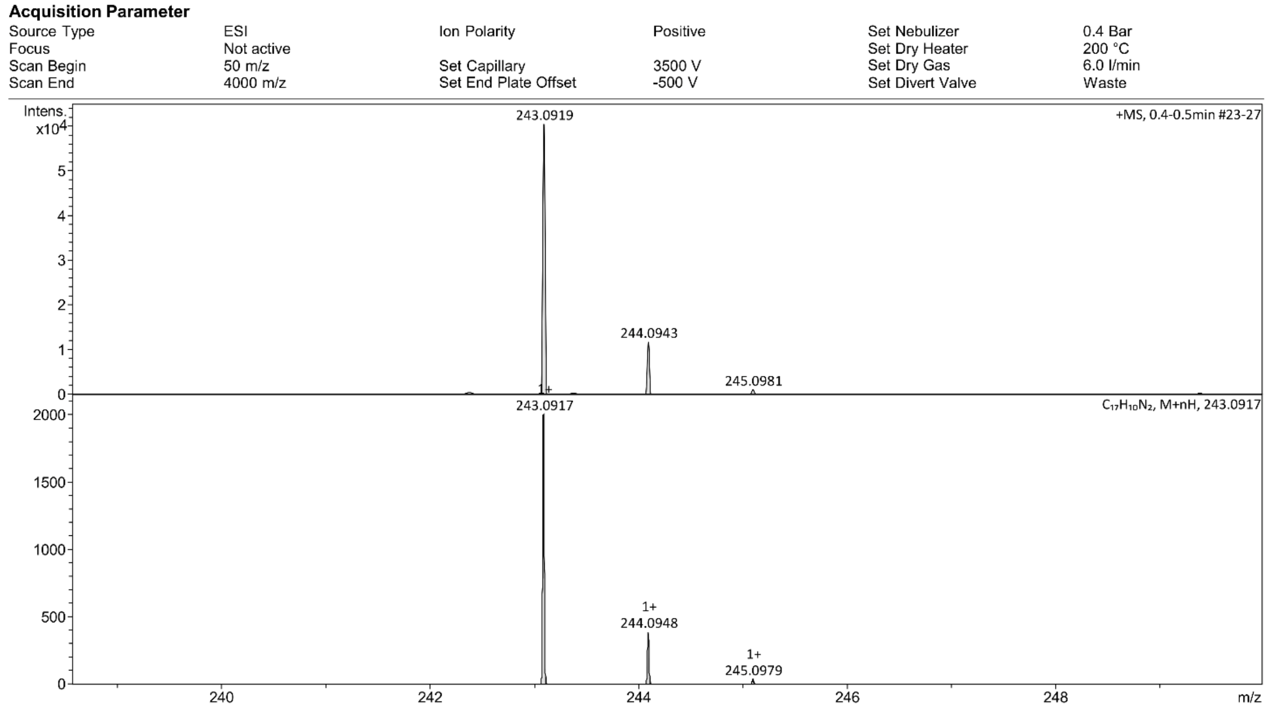

Figure **S12**. HRMS (ESI) trace of **4NICz**.

9-(3-bromopyridin-4-yl)-carbazole (Bromo-5PCz)

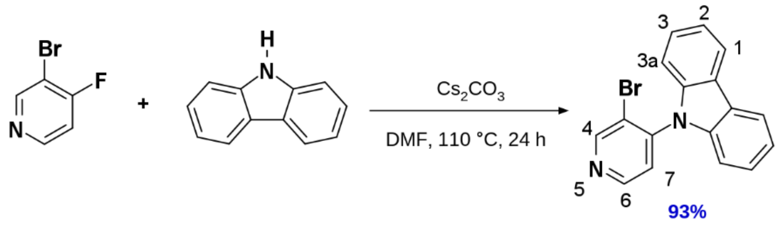

Adapted from reference [35]. Carbazole (0.25 g, 1.50 mmol, 1.0 equiv.) and Cs_2CO_3 (0.97 g, 3.00 mmol, 2.0 equiv.) were combined and degassed by three successive vacuum/nitrogen cycles. Under a flow of N_2, 3-bromo-4-fluoropyridine (0.23 mL, 2.24 mmol, 1.5 equiv.) and then DMF (3.0 mL) were added *via* syringe, forming an orange-yellow solution. The reaction mixture was then heated to 110 °C for approximately 24 h, after which a white precipitate had formed. After cooling to room-temperature, the reaction crude was poured into water, a liquid-liquid extraction was performed with EtOAc/brine, and the organic fractions were combined and dried over Na_2SO_4. The solvent was next evaporated to yield a yellow oil, which slowly solidified to form a yellow-brown solid. The crude was evaporated onto silica, before being purified by flash column chromatography in 15% EtOAc/Hexane. The fractions containing the product were combined and slowly evaporated until crystallization began. The evaporation was then stopped, and the product was allowed to crystallize overnight. The remainder of the solution was then removed, to **yield** the product as small white crystals (0.45 g, 93%). **^{1}H NMR (400 MHz; $(CD_3)_2SO$) δ_H (ppm)**: 9.14 (d, J = 0.5 Hz, 1H, H_4), 8.84 (d, J = 5.1 Hz, 1H, H_6), 8.28 (ddd, J = 7.9, 1.3, 0.7 Hz, 2H, H_1), 7.80 (dd, J = 5.1, 0.5 Hz, 1H, H_7), 7.45 (ddd, J = 8.2, 7.2, 1.3 Hz, 2H, H_3), 7.34 (ddd, J = 7.9, 7.2, 1.0 Hz, 2H, H_2), and 7.15 (ddd, J = 8.2, 1.0, 0.7 Hz, 2H, H_{3a}). **^{13}C{^{1}H} NMR (101 MHz; $(CD_3)_2SO$) δ_C (ppm)**: 154.4 (CH), 151.2 (CH), 144.0 (C_{quat}), 139.8 (C_{quat}), 126.9 (CH), 126.2 (CH), 123.5 (C_{quat}), 121.2 (CH), 121.2 (CH), 120.9 (C_{quat}), 110.6 (CH). Characterization data matches those in the literature.[35]

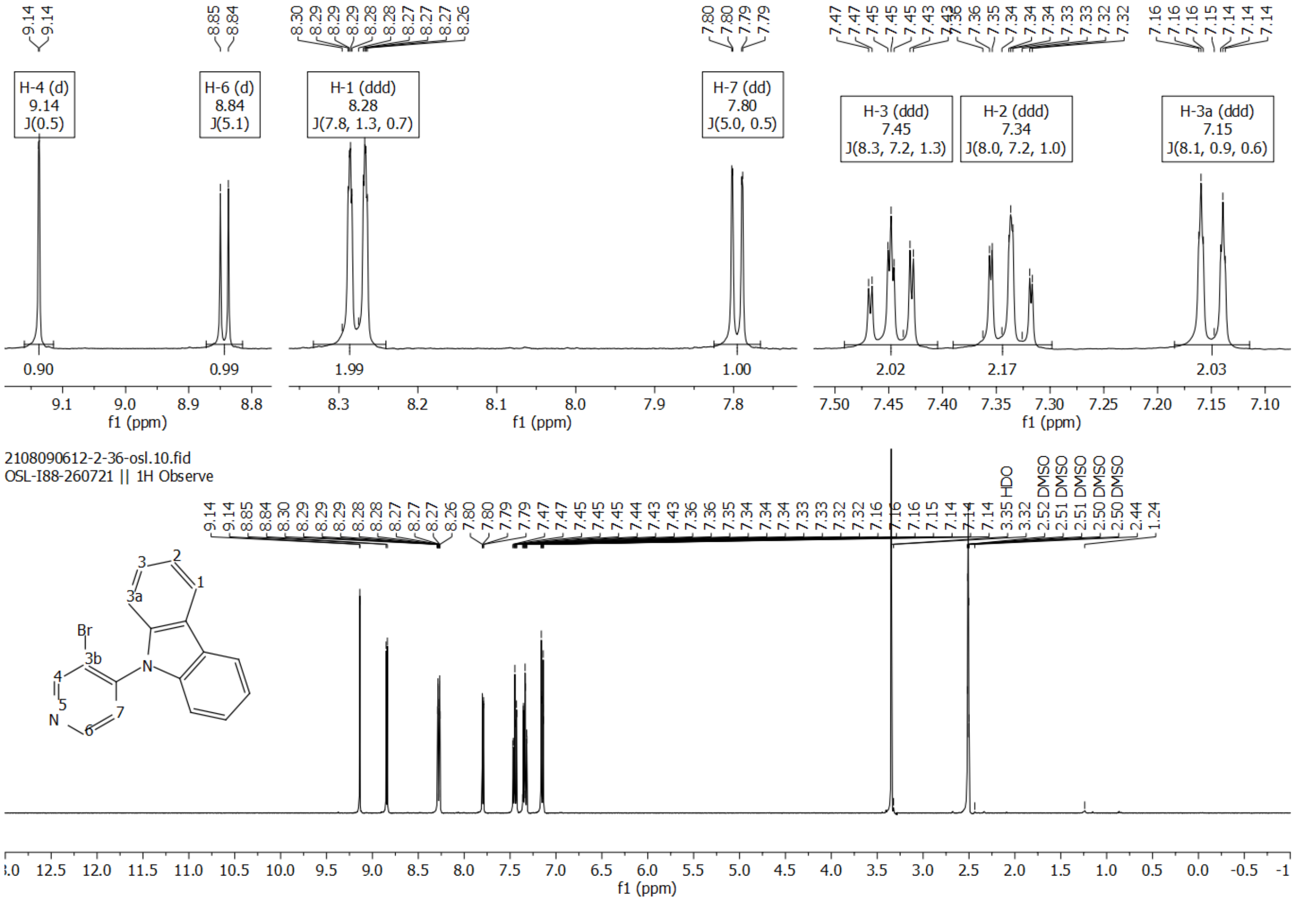

Figure **S13**. ^{1}H NMR of **Bromo-5PCz** in d_6-DMSO.

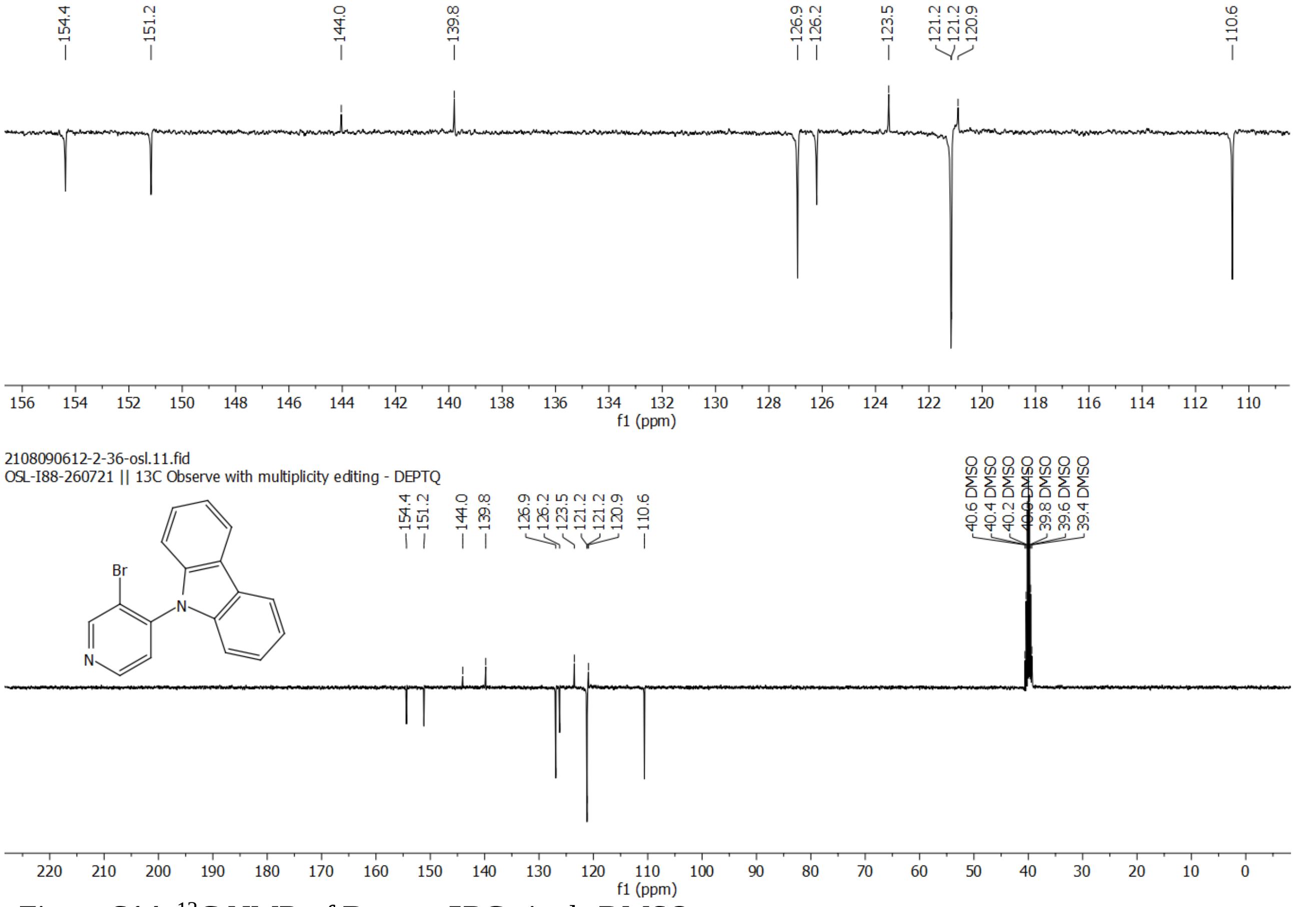

Figure **S14**. ^{13}C NMR of **Bromo-5PCz** in d_6-DMSO.

Pyrido[3’,4’:4,5]pyrrolo[3,2,1-jk]carbazole (5NICz)

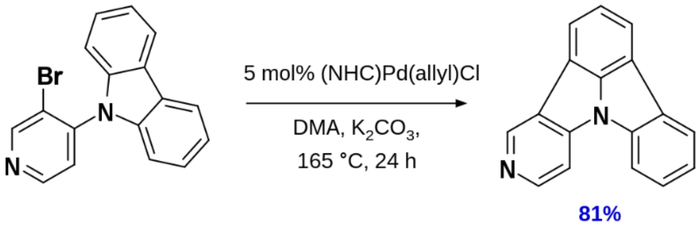

Adapted from reference [35]. 9-(3-bromopyridin-4-yl)-carbazole (0.43 g, 1.33 mmol, 1.0 equiv.), allyl[1,3-bis(2,6-diisopropyl-phenyl)imidazol-2-ylidene]chloropalladium(II) (0.04 g, 0.07 mmol, 5 mol%), and K_2CO_3 (0.37 g, 2.66 mmol, 2.0 equiv.) were combined and degassed by three successive vacuum/nitrogen cycles. Under a flow of N_2, DMA (13.0 mL) was added *via* syringe to form a pale-yellow solution with a white suspension. The reaction mixture was then heated to 165 °C for approximately 24 h, after which it had turned black. After cooling to room-temperature, the reaction crude was filtered through celite with EtOAc to remove excess Pd, revealing a yellow-brown solution, and the solvent was removed to form a white-brown solid. A liquid-liquid extraction was then performed with EtOAc/Brine and the organic fractions were combined and dried over Na_2SO_4. The crude was next evaporated onto silica and purified by flash column chromatography in 70% EtOAc/hexane to yield the crude product as a white powder after removing the solvent (0.31 g). The crude product was then recrystallised from the minimum amount of boiling 40% EtOAc/Hexane to yield long, white crystals (0.22 g). The filtrate was evaporated and recrystallised in 40% EtOAc/Hexane to yield a second crop of crystals (0.04 g), for a total **yield** of 0.26 g (81%). **R*f***: 0.29 (80 : 20 EtOAc : hexane). **Mp:** 179 – 181 °C. Purity by **HPLC**: 99.7%. **^{1}H NMR (400 MHz; $(CD_3)_2SO$) δ_H (ppm)**: 9.48 (d, *J* = 1.0 Hz, 1H), 8.73, (d, *J* = 5.6 Hz, 1H), 8.39 (dt, *J* = 8.1, 0.9 Hz, 1H), 8.37 (dd, *J* = 5.6, 1.0 Hz, 1H), 8.32 (dt, *J* = 7.8, 1.0 Hz, 1H), 8.28 (d, *J* = 7.5 Hz, 1H), 8.26 (d, *J* = 7.3 Hz, 1H), 7.72 (t, *J* = 7.4 Hz, 1H), 7.68 (ddd, *J* = 8.3, 7.4, 1.2 Hz, 1H), and 7.50 (td, *J* = 7.6, 1.0 Hz, 1H). **$^{13}C\{^1H\}$ NMR (101 MHz; $(CD_3)_2SO$) δ_C (ppm)**: 174.4 (CH), 145.3 (CH). 143.3 (C_{quat}), 142.3 (C_{quat}), 138.1 (C_{quat}), 130.3 (C_{quat}), 128.0 (CH), 125.7 (C_{quat}), 124.7 (CH), 124.0 (CH), 123.7 (CH), 121.3 (CH), 121.2 (CH), 118.7 (C_{quat}), 116.2 (C_{quat}), 114.1 (CH), 180.7 (CH). **HR-MS[M+H]$^+$**: Calculated: ($C_{17}H_{11}N_2$): 243.0917; Found: 243.0905. Characterization data matches those in the literature.[35]

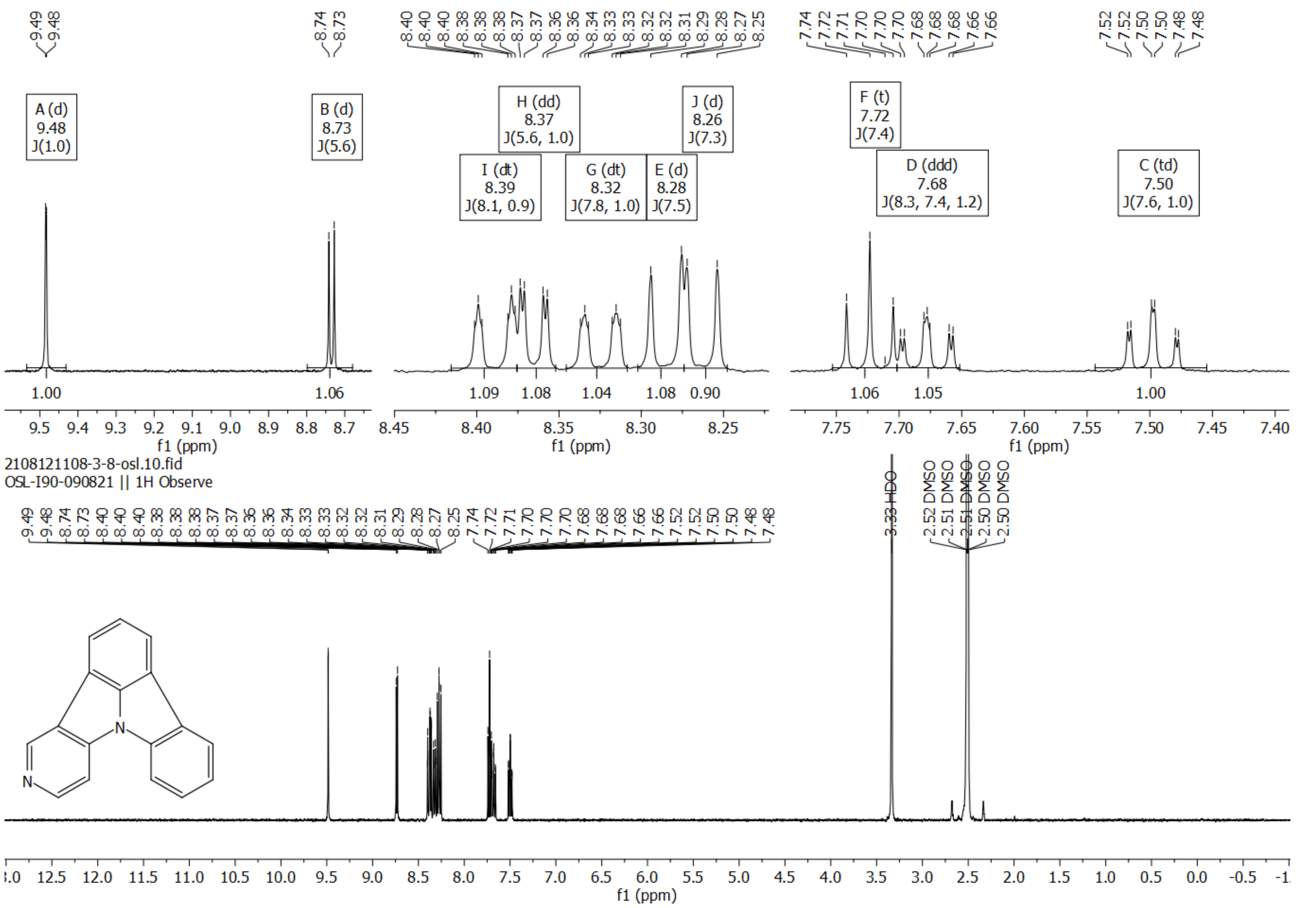

Figure **S15**. ^{1}H NMR of **5NICz** in d$_6$-DMSO.

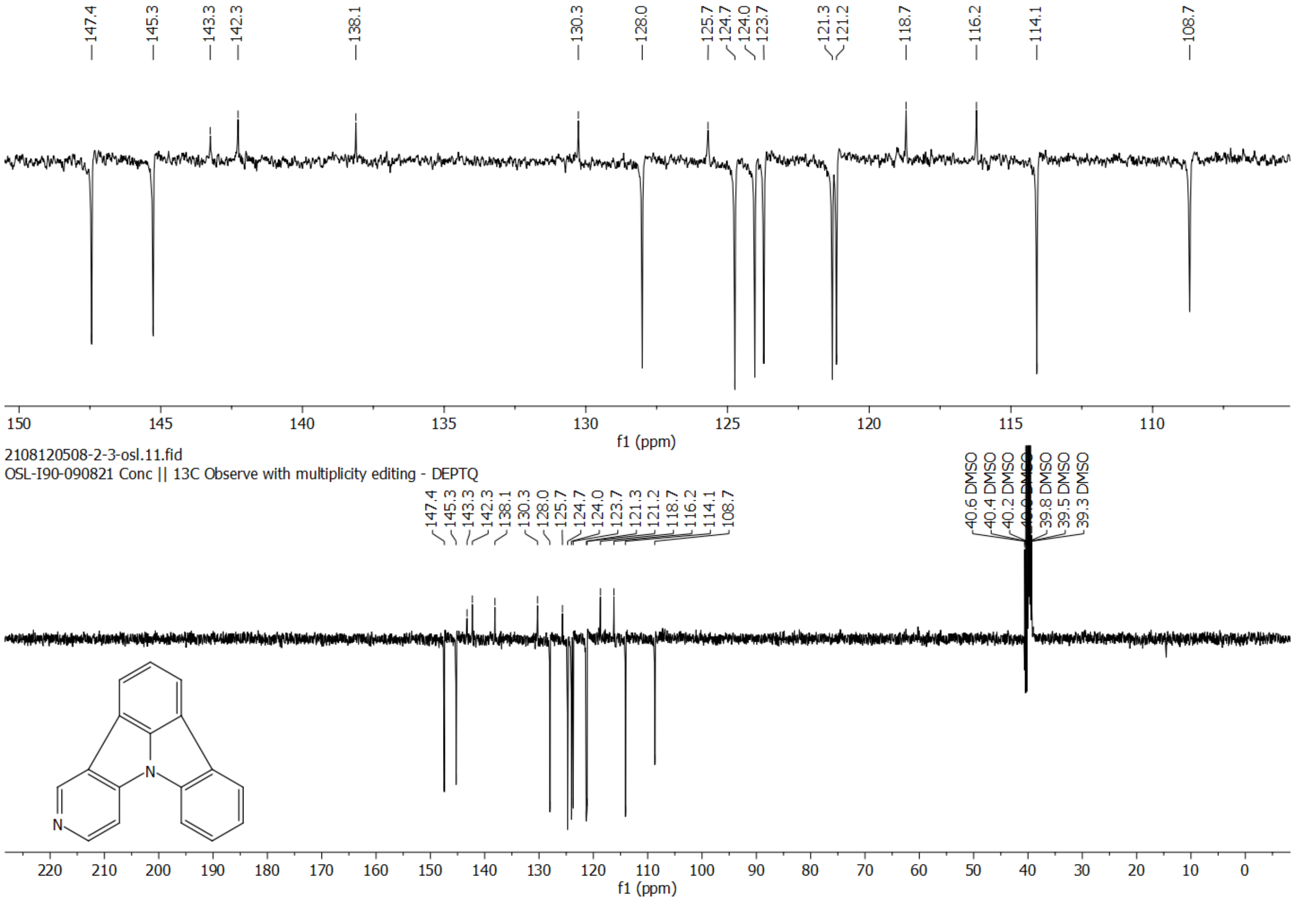

Figure **S16**. ^{13}C NMR of **5NICz** in d$_6$-DMSO.

<Sample Information>

Sample Name	: I90		
Sample ID	:		
Method Filename	: 50% Acetonitrile 50 Water 20 mins.lcm		
Batch Filename	: Re run 3 on 01092021.lcb		
Vial #	: 2-10	Sample Type	: Unknown
Injection Volume	: 5 uL		
Date Acquired	: 01/09/2021 17:46:24	Acquired by	: System Administrator
Date Processed	: 12/12/2023 15:08:00	Processed by	: System Administrator

<Chromatogram>

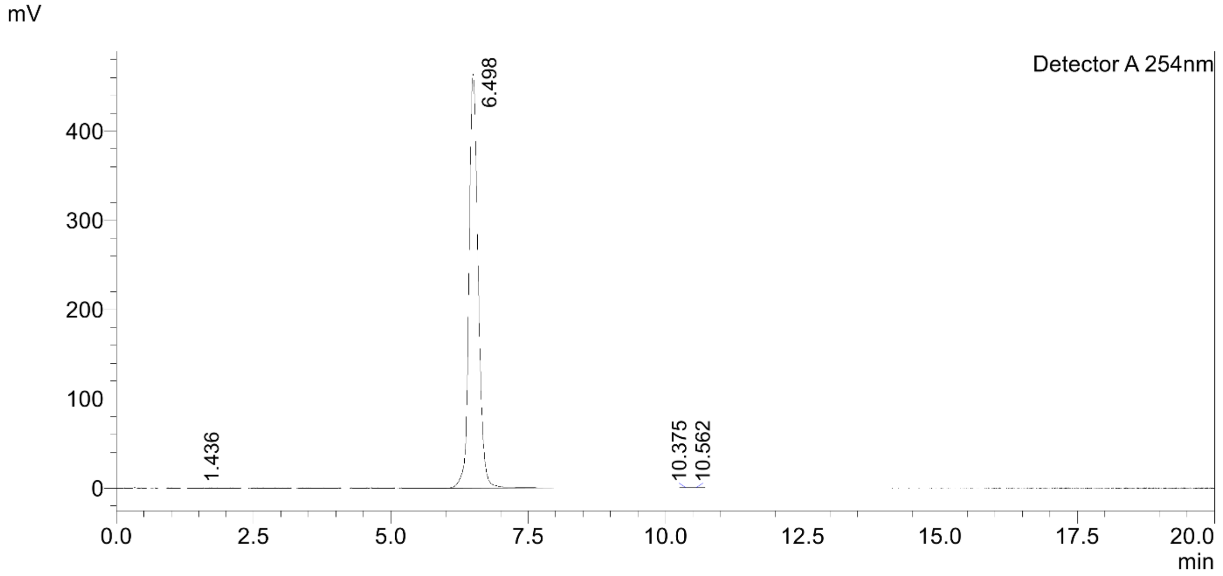

<Peak Table>

Detector A 254nm

Peak#	Ret. Time	Area	Height	Area%	Area/Height	Width at 5% Height
1	1.436	1219	205	0.022	5.946	0.217
2	6.498	5407770	463148	99.725	11.676	0.405
3	10.375	4985	388	0.092	12.835	--
4	10.562	8709	463	0.161	18.809	--
Total		5422683	464204	100.000		

Figure **S17**. HPLC trace of **5NICz**.

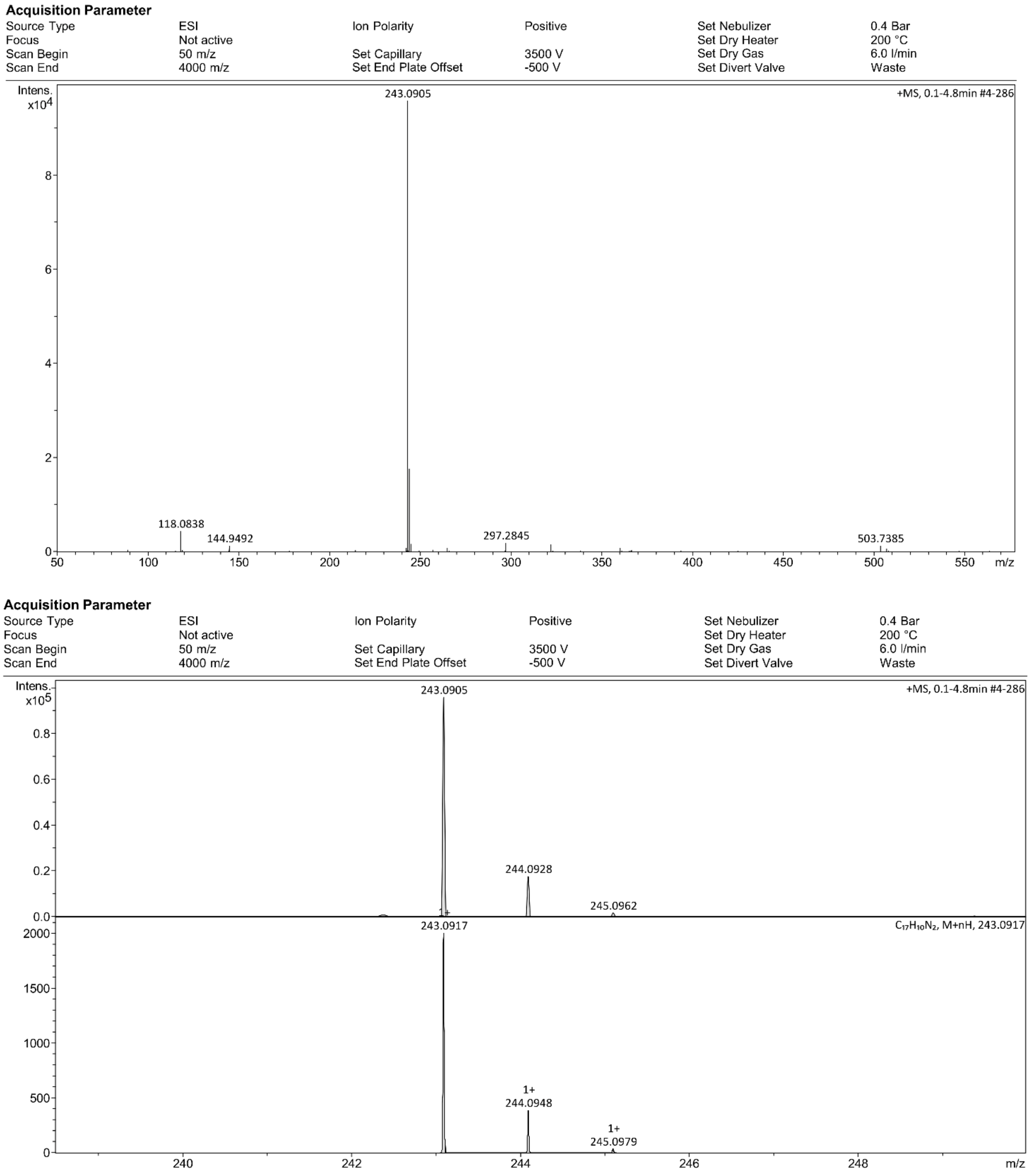

Figure **S18**. HRMS (ESI) trace of **5NICz**.

9-(4-chloropyridin-3-yl)carbazole (Chloro-6PCz)

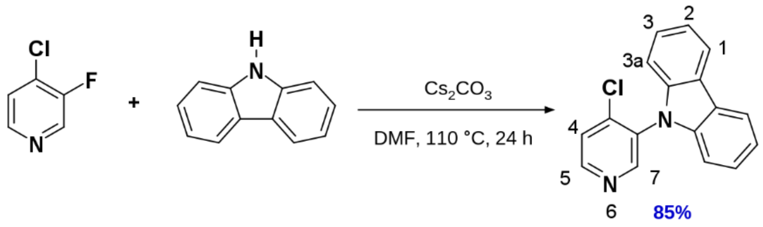

Protocol adapted from the literature.[35] Carbazole (0.50 g, 2.99 mmol, 1.0 equiv.) and Cs_2CO_3 (1.95 g, 5.98 mmol, 2.0 equiv.) were combined and degassed by three successive vacuum/nitrogen cycles. Under a flow of N_2, 4-chloro-3-fluoropyridine (0.65 mL, 6.59 mmol, 2.2 equiv.) and then DMF (6.0 mL) were added *via* syringe, forming a deep-red solution. The reaction mixture was then heated to 110 °C for approximately 24 h, after which a brown suspension had formed. After cooling to room-temperature, the reaction crude was poured into water to form a milky white suspension. The crude was then extracted from DCM/H_2O before the organic fractions were combined and dried over $MgSO_4$. The solvent was next evaporated to yield a brown-red solid which was evaporated onto silica. The crude was then purified by flash chromatography in 10% EtOAc/Hexane before finally being evaporated to **yield** a yellow oil (0.71 g, 85%), which was used without further purification. **^{1}H NMR (400 MHz; $(CD_3)_2SO$) δ_H (ppm)**: 8.89 (s, 1H, H_7), 8.81 (d, *J* = 5.4 Hz, H1, H_5), 8.28 (d, *J* = 7.7 Hz, 2H, H_1), 7.98 (dd, *J* = 5.4, 1.1 Hz, 1H, H_4), 7.44 (ddd, *J* = 8.2, 7.4, 1.2 Hz, 2H, H_3), 7.33 (dd, *J* = 7.7, 7.4 Hz, 2H, H_2), and 7.10 (d, *J* = 8.2 Hz, 2H, H_{3a}). **^{13}C{^{1}H} NMR (101 MHz; $(CD_3)_2SO$) δ_C (ppm)**: 152.2 (CH), 151.7 (CH), 142.8 (C_{quat}), 140.8 (C_{quat}), 131.7 (C_{quat}), 127.0 (CH), 126.3 (CH), 123.3 (C_{quat}), 121.1 (CH), 121.0 (CH), and 110.2 (CH). Characterization data matches those in the literature.[35]

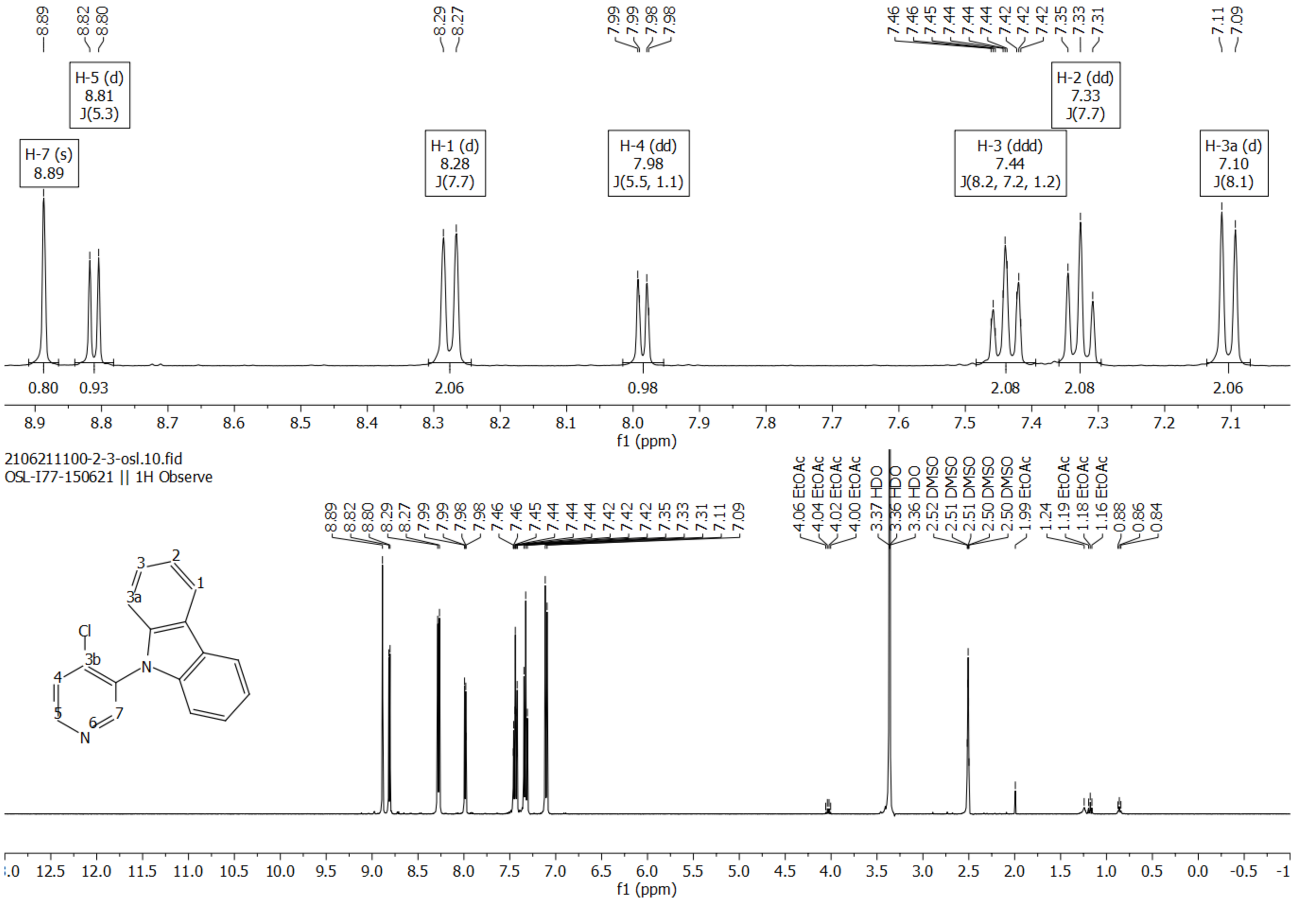

Figure **S19**. ^{1}H NMR of **Chloro-6PCz** in d_6-DMSO.

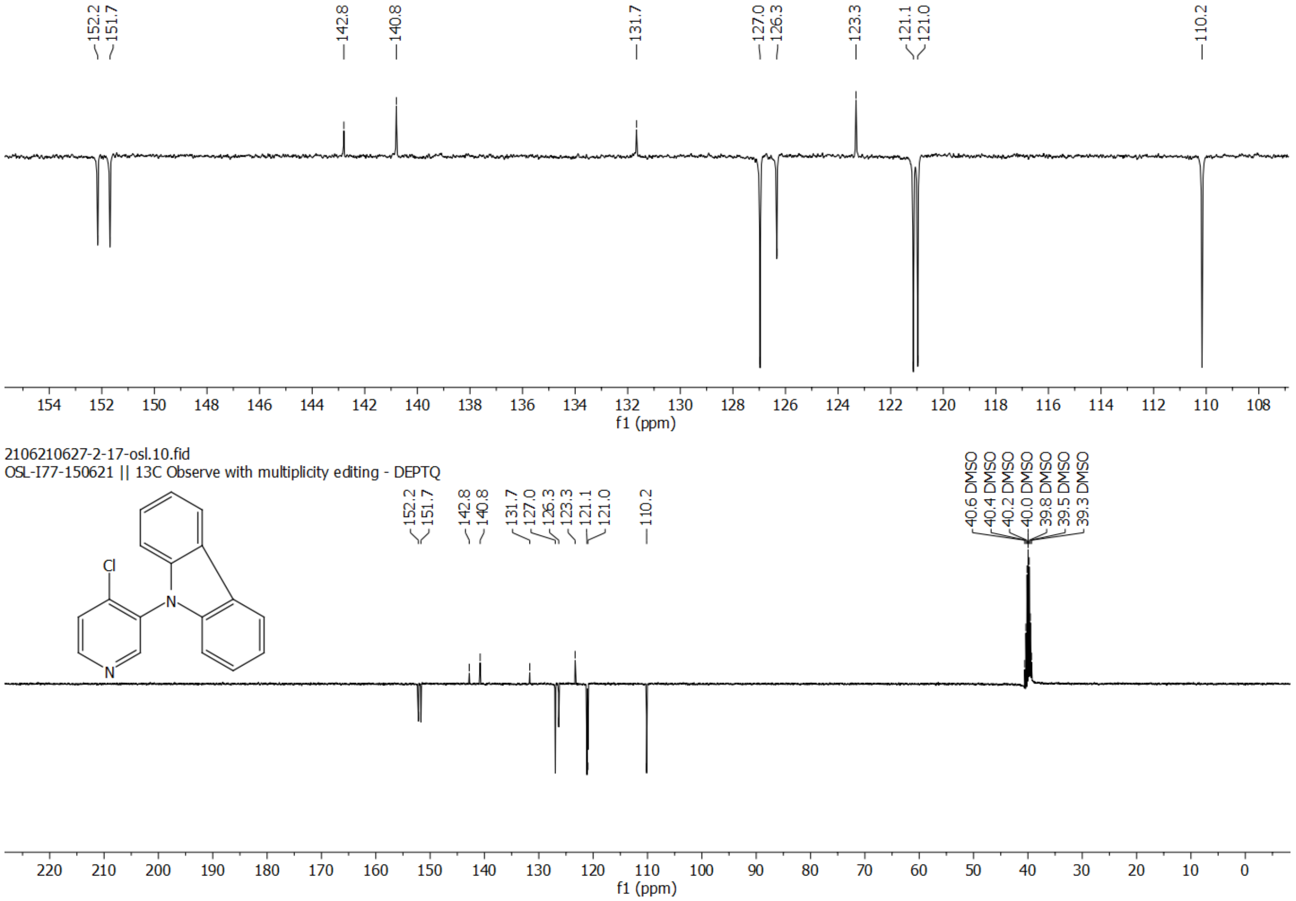

Figure **S20**. ^{13}C NMR of **Chloro-6PCz** in d_6-DMSO.

Pyrido[4',3':4,5]pyrrolo[3,2,1-jk]carbazole (6NICz)

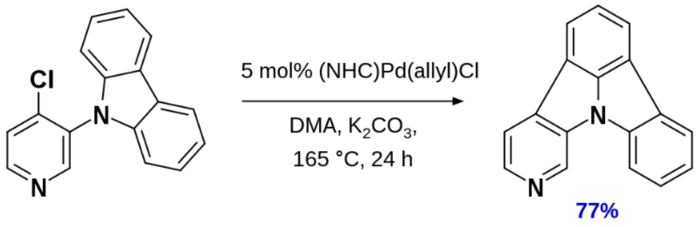

Protocol adapted from the literature.[35] 9-(4-chloropyridin-3-yl)carbazole (0.71 g, 2.55 mmol, 1.0 equiv.) was dissolved in EtOAc and washed into a reaction vessel before the solvent was removed. allyl[1,3-bis(2,6-diisopropyl-phenyl)imidazol-2-ylidene]chloropalladium(II) (73 mg, 0.13 mmol, 5 mol%), and K_2CO_3 (0.70 g, 5.10 mmol, 2.0 equiv.) were combined and degassed by three successive vacuum/nitrogen cycles. Under a flow of N_2, DMA (25.0 mL) was added *via* syringe to form a clear suspension. The reaction mixture was then heated to 165 °C for approximately 24 h, after which it had turned black. After cooling to room-temperature, the reaction crude was filtered through celite with EtOAc to remove excess Pd and the solvent was evaporated to reveal a grey solid. A liquid-liquid extraction was then performed with EtOAc/Brine and the organic fractions were combined and dried over $MgSO_4$. The crude was next evaporated onto silica and purified by flash column chromatography in 80% EtOAc/hexane to yield the crude product as a white powder after removing the solvent (0.56 g). The crude product was then recrystallised from the minimum amount of boiling 50% EtOAc/Hexane to yield needle-like, yellow crystals (0.39 g). The filtrate was evaporated and recrystallised in 50% EtOAc/Hexane to yield a second crop of crystals (0.08 g), for a total **yield** of 0.47 g 77%). **R_f**: 0.55 (EtOAc). **Mp**: 164 – 165 °C. Purity by **HPLC**: 99.9%. **^{1}H NMR (400 MHz; $(CD_3)_2SO$) δ_H (ppm)**: 9.69 (d, J = 1.1 Hz, 1H), 8.64 (d, J = 5.1 Hz, 1H), 8.40 (ddd, J = 8.0, 0.9, 0.8, Hz, 1H), 8.36 (d, J = 7.3 Hz, 1H), 8.35 – 8.30 (m, 3H), 7.73 (t, J = 7.5 Hz, 1H), 7.68 (ddd, J = 8.3, 7.4, 1.3 Hz, 1H), and 7.48 (ddd, J = 8.5, 7.6, 0.9 Hz, 1H). **^{13}C{^{1}H} NMR (101 MHz; $(CD_3)_2SO$) δ_C (ppm)**: 143.8 (C_{quat}), 142.8 (CH), 138.4 (C_{quat}), 135.4 (CH), 135.3 (C_{quat}), 134.6 (C_{quat}), 129.6 (C_{quat}), 128.1 (CH), 124.2 (CH), 124.0 (CH), 123.1 (CH), 122.8 (CH), 122.1 (CH), 119.2 (C_{quat}), 118.4 (CH), 116.4 (C_{quat}), and 113.8 (CH). **HR-MS[M+H]$^+$**: Calculated: ($C_{17}H_{11}N_2$): 243.0917; Found: 243.0915. Characterization data matches those in the literature.[35]

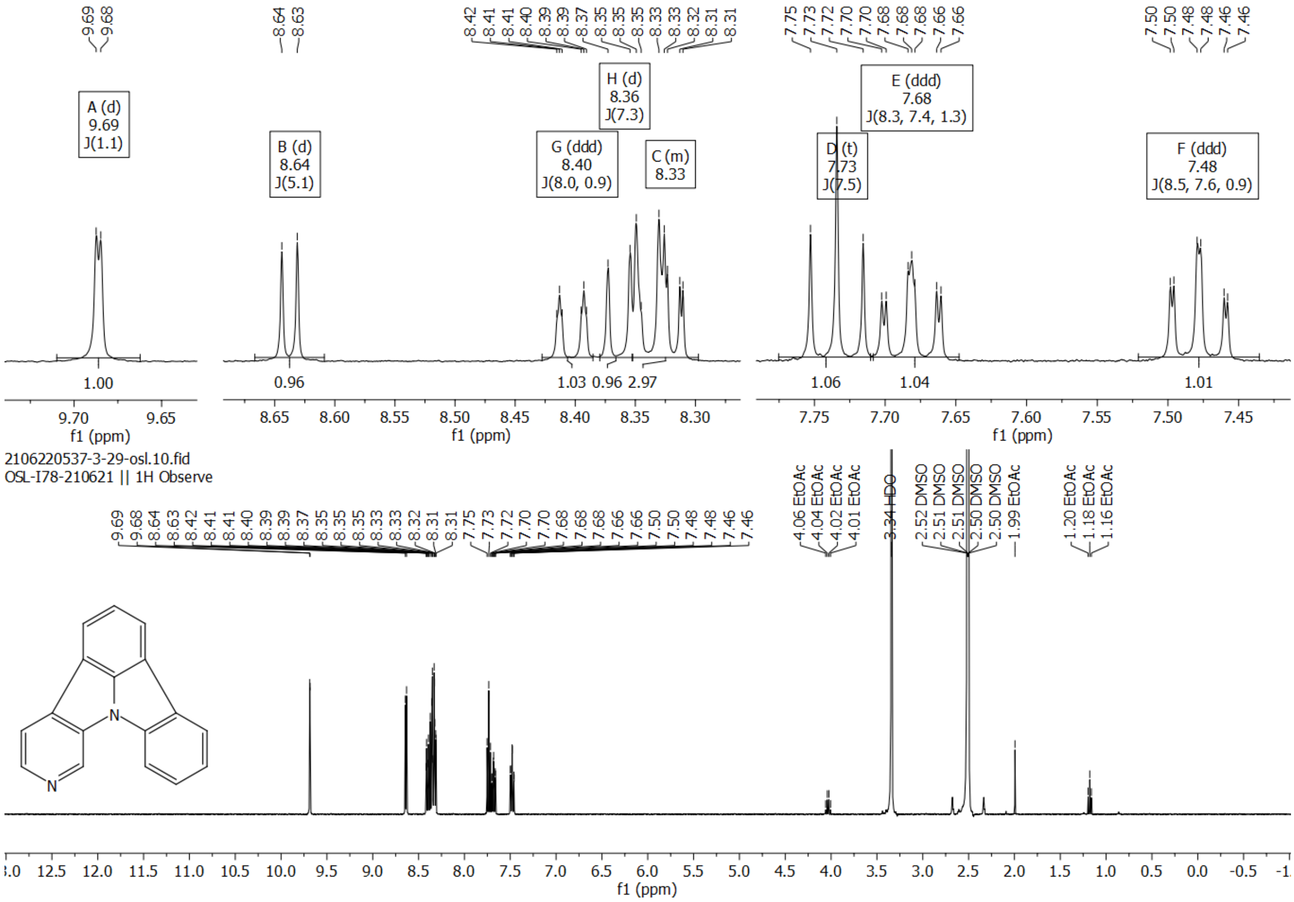

Figure **S21**. ^{1}H NMR of **6NICz** in d_6-DMSO.

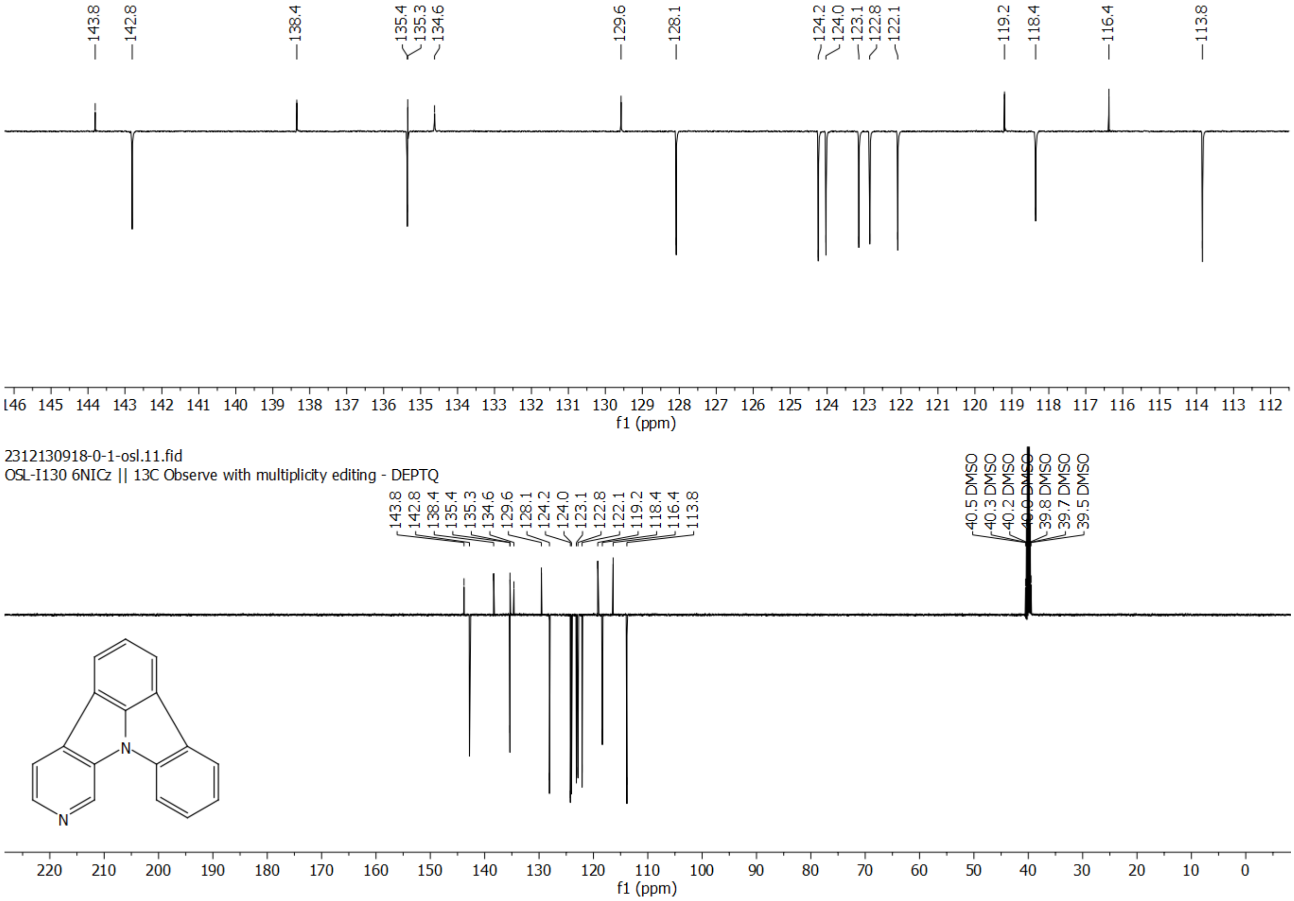

Figure **S22**. ^{13}C NMR of **6NICz** in d_6-DMSO.

<Sample Information>

Sample Name	: I78		
Sample ID	:		
Method Filename	: 50% Acetonitrile 50 Water 20 mins.lcm		
Batch Filename	: Re run 3 on 01092021.lcb		
Vial #	: 2-7	Sample Type	: Unknown
Injection Volume	: 5 uL		
Date Acquired	: 01/09/2021 16:14:03	Acquired by	: System Administrator
Date Processed	: 12/12/2023 15:03:42	Processed by	: System Administrator

<Chromatogram>

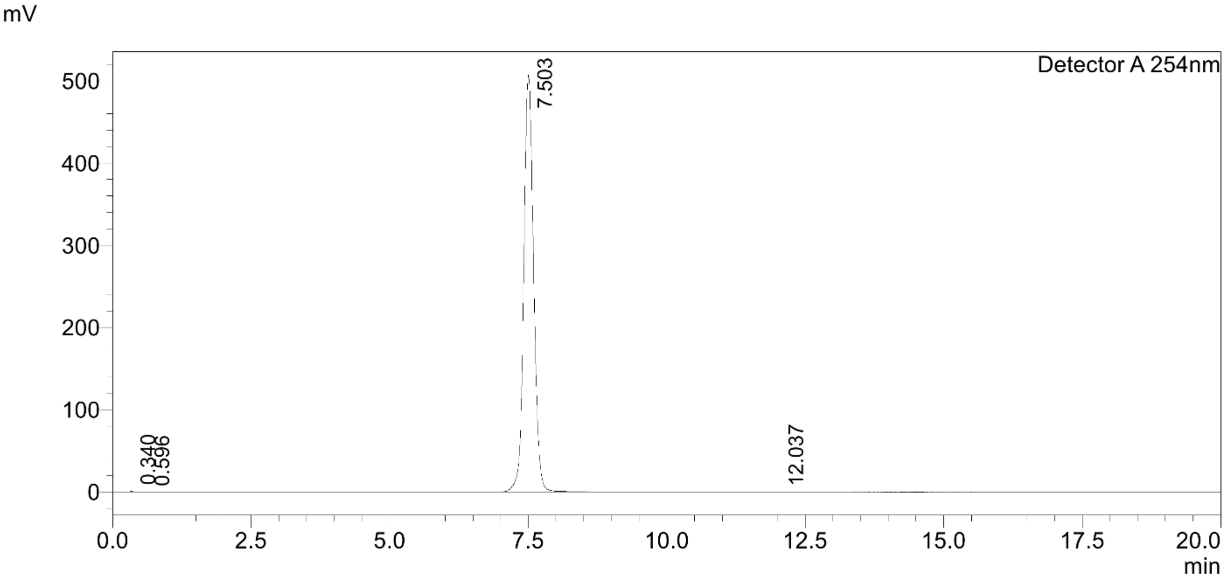

<Peak Table>

Detector A 254nm

Peak#	Ret. Time	Area	Height	Area%	Area/Height	Width at 5% Height
1	0.340	3540	1522	0.056	2.326	0.086
2	0.596	1017	117	0.016	8.669	--
3	7.503	6301285	507036	99.851	12.428	0.419
4	12.037	4845	204	0.077	23.778	0.689
Total		6310687	508879	100.000		

Figure **S23**. HPLC trace of **6NICz**.

Acquisition Parameter

Source Type	ESI	Ion Polarity	Positive	Set Nebulizer	0.4 Bar
Focus	Not active			Set Dry Heater	200 °C
Scan Begin	50 m/z	Set Capillary	3000 V	Set Dry Gas	6.0 l/min
Scan End	4000 m/z	Set End Plate Offset	-500 V	Set Divert Valve	Waste

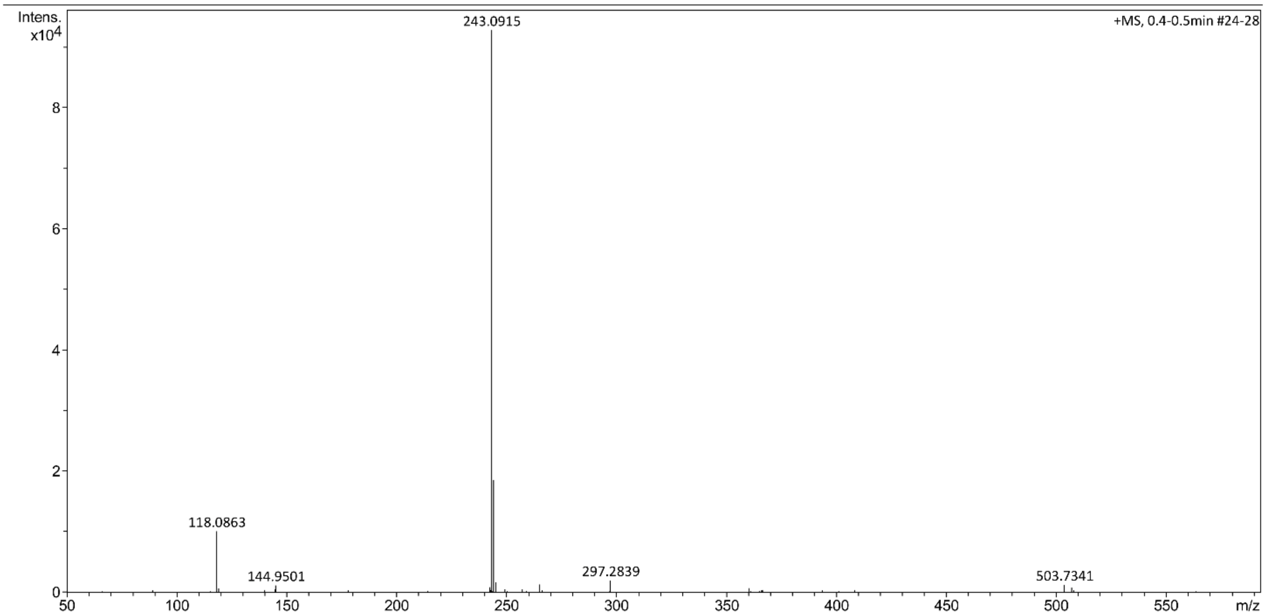

Acquisition Parameter

Source Type	ESI	Ion Polarity	Positive	Set Nebulizer	0.4 Bar
Focus	Not active			Set Dry Heater	200 °C
Scan Begin	50 m/z	Set Capillary	3000 V	Set Dry Gas	6.0 l/min
Scan End	4000 m/z	Set End Plate Offset	-500 V	Set Divert Valve	Waste

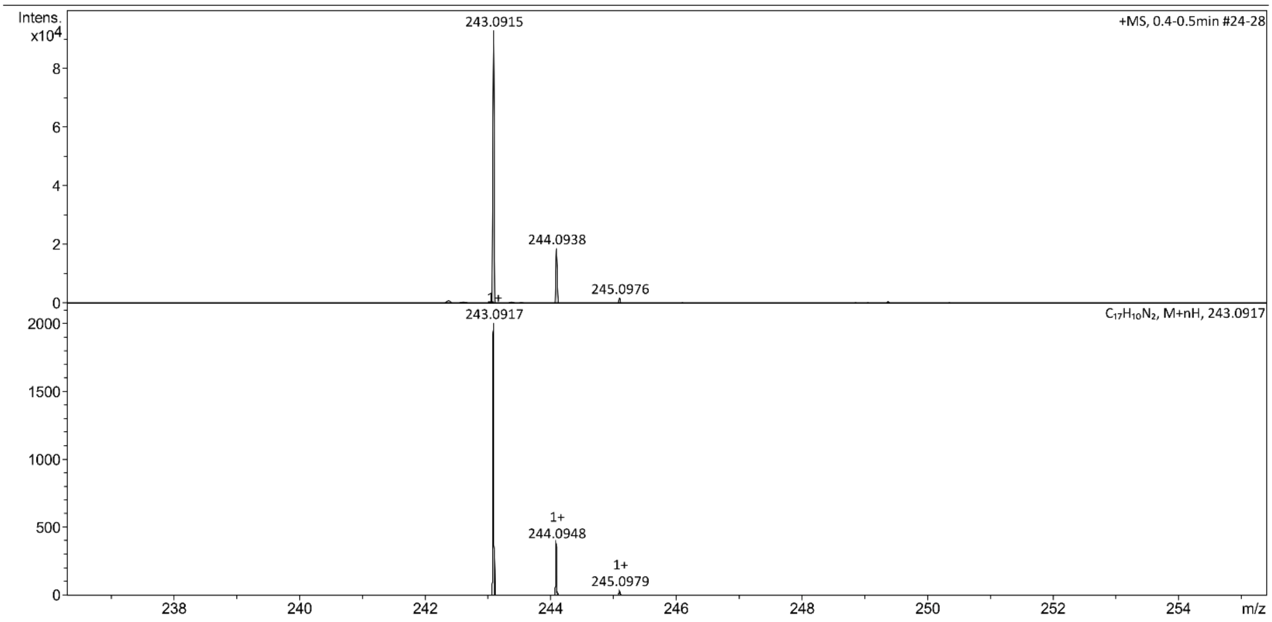

Figure **S24**. HRMS (ESI) trace of **6NICz**.

9-(3-chloropyridin-2-yl)carbazole (Chloro-7PCz)

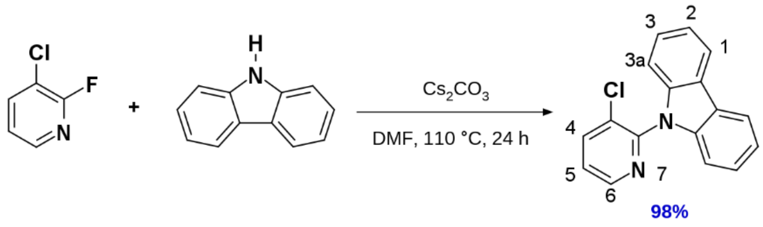

Protocol adapted from the literature.[35] Carbazole (0.50 g, 2.99 mmol, 1.0 equiv.) and Cs_2CO_3 (1.95 g, 5.98 mmol, 2.0 equiv.) were combined and degassed by three successive vacuum/nitrogen cycles. Under a flow of N_2, 3-chloro-2-fluoropyridine (0.59 mL, 5.98 mmol, 2.0 equiv.) and then DMF (6.0 mL) were added *via* syringe, forming a clear solution with white solid. The reaction mixture was then heated to 110 °C for approximately 24 h, after which a pale-yellow solution with a creamy white precipitate had formed. After cooling to room-temperature, the reaction crude was poured into brine and then extracted from EtOAc/brine before the organic fractions were combined and dried over Na_2SO_4. The EtOAc solution was then directly evaporated onto silica and the crude was purified by flash chromatography in 12.5% EtOAc/petroleum ether (40:60). The fractions were combined and evaporated to yield a clear oil (0.85 g). The oil was dissolved in hot MeOH/EtOAc and allowed to cool to room temperature. The solvent was then evaporated and the resulting oil was washed with petroleum ether to remove residual EtOAc. The petroleum ether was evaporated and the oil was dried under high vacuum overnight to **yield** a clear oil (0.82 g, 98%), which was used without further purification. **^{1}H NMR (400 MHz; $(CD_3)_2SO$) δ_H (ppm)**: 8.73 (dd, J = 4.7, 1.6 Hz, 1H, H_6), 8.39 (dd, J = 8.1, 1.6 Hz, 1H, H_4), 8.26 (ddd, J = 8.0, 1.3, 0.8 Hz, 2H, H_1), 7.73 (dd, J = 8.1, 4.7 Hz, 1H, H_5), 7.44 (ddd, J = 8.2, 7.2, 1.3 Hz, 2H, H_3), 7.32 (ddd, J = 8.0, 7.2, 1.0 Hz, 2H, H_2), and 7.18 (ddd, J = 8.2, 1.0, 0.8 Hz, 2H, H_{3a}). **^{13}C{^{1}H} NMR (101 MHz; $(CD_3)_2SO$) δ_C (ppm)**: 149.1 (CH), 147.3 (C_{quat}), 141.1 (CH), 139.9 (C_{quat}), 129.1 (C_{quat}), 126.7 (CH), 126.2 (CH), 123.4 (C_{quat}), 121.1 (CH), 121.0 (CH), 111.1 (CH). Characterization data matches those in the literature.[35]

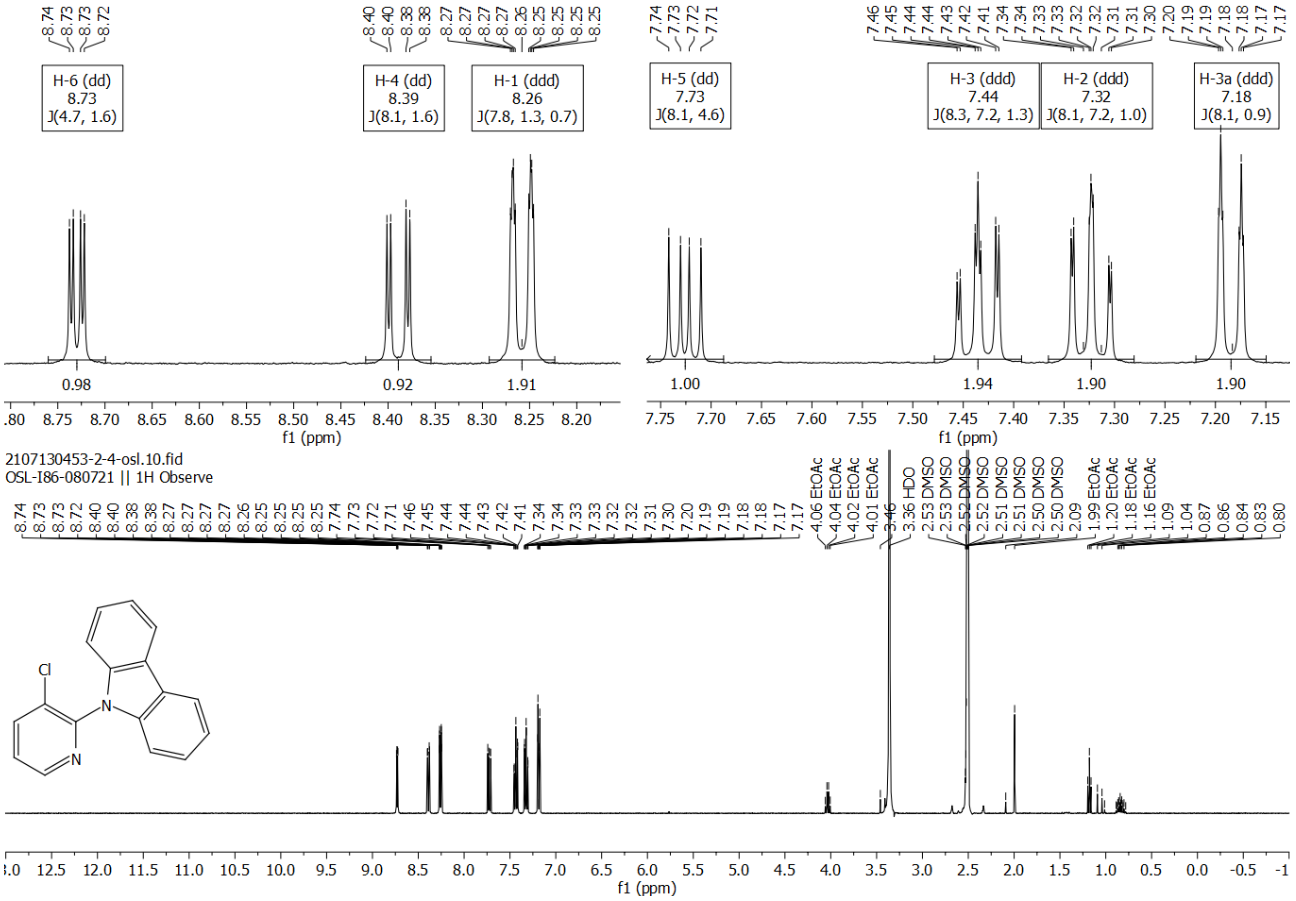

Figure **S25**. ^{1}H NMR of **Chloro-7PCz** in d_6-DMSO.

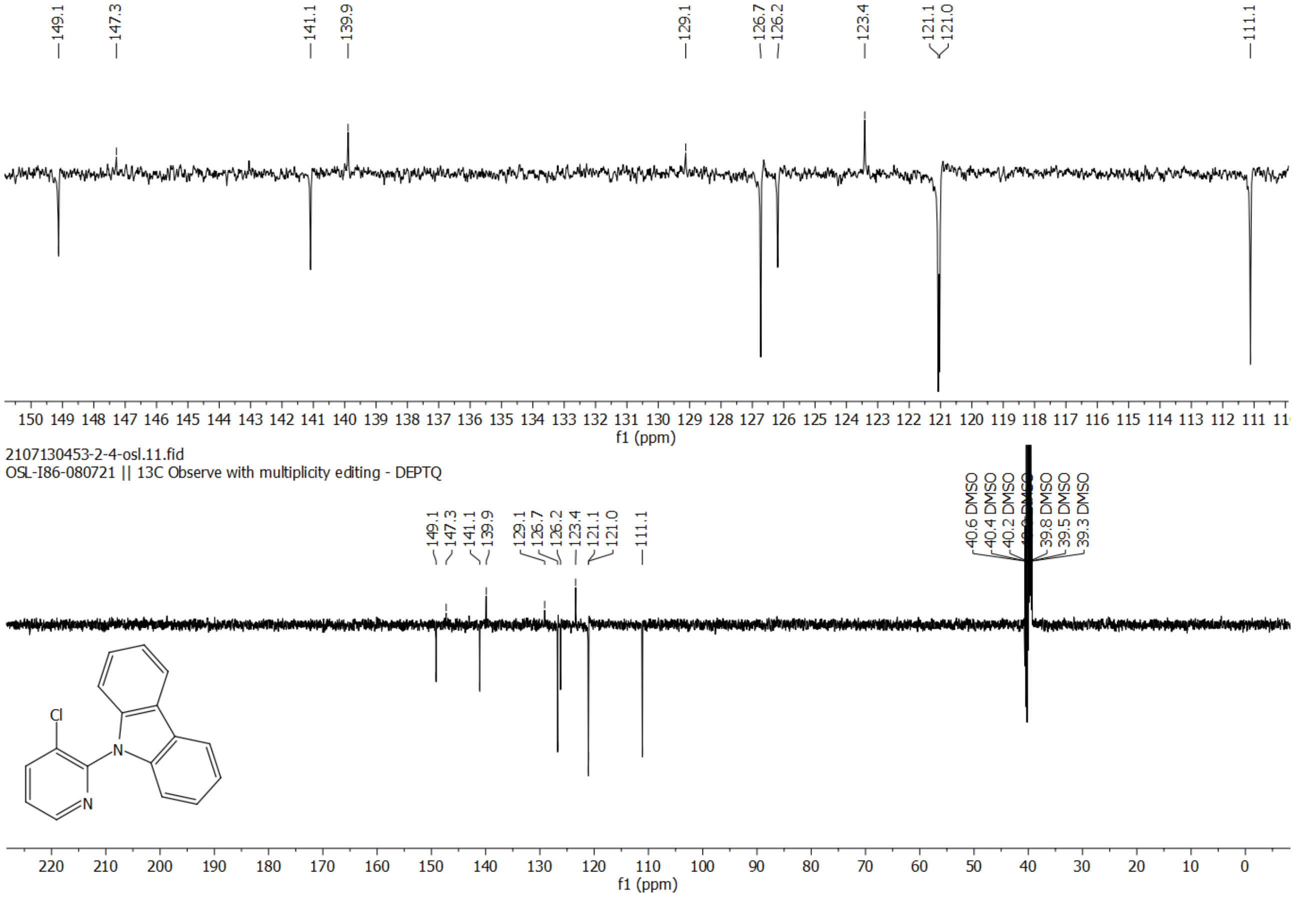

Figure **S26**. ^{13}C NMR of **Chloro-7PCz** in d_6-DMSO.

Pyrido[3',2':4,5]pyrrolo[3,2,1-jk]carbazole (7NICz)

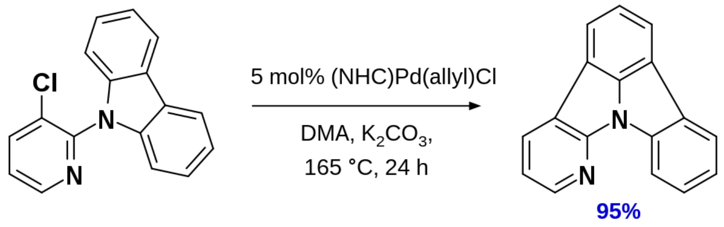

Protocol adapted from the literature.[35] 9-(3-chloropyridin-2-yl)carbazole (0.82 g, 2.94 mmol, 1.0 equiv.) was dissolved in EtOAc and washed into a reaction vessel before the solvent was removed. allyl[1,3-bis(2,6-diisopropyl-phenyl)imidazol-2-ylidene]chloropalladium(II) (84 mg, 0.15 mmol, 5 mol%), and K_2CO_3 (0.81 g, 5.89 mmol, 2.0 equiv.) were combined and degassed by three successive vacuum/nitrogen cycles. Under a flow of N_2, DMA (30.0 mL) was added *via* syringe to form a milky white suspension. The reaction mixture was then heated to 165 °C for approximately 24 h, after which it had turned dark red. After cooling to room-temperature, the reaction crude was filtered through celite with EtOAc to remove excess Pd and the solvent was evaporated to reveal a pale purple solid. A liquid-liquid extraction was then performed with EtOAc/Brine and the organic fractions were combined and dried over Na_2SO_4. The crude was next evaporated onto silica and purified by flash column chromatography in 20% EtOAc/hexane. The organic fractions were combined and slowly evaporated under reduced pressure until crystallisation began, at which point evaporation was ceased and the product was allowed to crystallize overnight to yield small white crystals (0.68 g, 95%). **R_f**: 0.50 (20 : 80 EtOAc : hexane). **Mp**: 158 – 160 °C. Purity by **HPLC**: 99.1%. **^{1}H NMR (400 MHz; $(CD_3)_2SO$) δ_H (ppm)**: 8.69 (dd, J = 7.8, 1.6 Hz, 1H), 8.60 (dd, J = 5.0, 1.6 Hz, 1H), 8.32 (ddd, J = 7.8, 1.2, 0.7 Hz, 1H), 8.27 (d, J = 7.5 Hz, 1H), 8.27 – 8.20 (m, 2H), 7.70 (t, J = 7.5 Hz, 1H), 7.67 (ddd, J = 8.1, 6.9, 1.3 Hz, 1H), and 7.51 – 7.44 (m, 2H). **^{13}C{^{1}H} NMR (101 MHz; $(CD_3)_2SO$) δ_C (ppm)**: 150.5 (C_{quat}), 146.7 (CH), 142.4 (C_{quat}), 137.7 (C_{quat}), 132.2 (CH), 130.0 (C_{quat}), 128.0 (CH), 124.2 (CH), 124.0 (CH), 123.4 (CH), 123.3 (C_{quat}), 121.4 (CH), 121.3 (CH), 118.8 (C_{quat}),118.3 (CH), 116.0 (C_{quat}), 113.9 (CH). **HR-MS[M+H]$^+$**: Calculated: ($C_{17}H_{11}N_2$): 243.0917; Found: 243.0924. Characterization data matches those in the literature.[35]

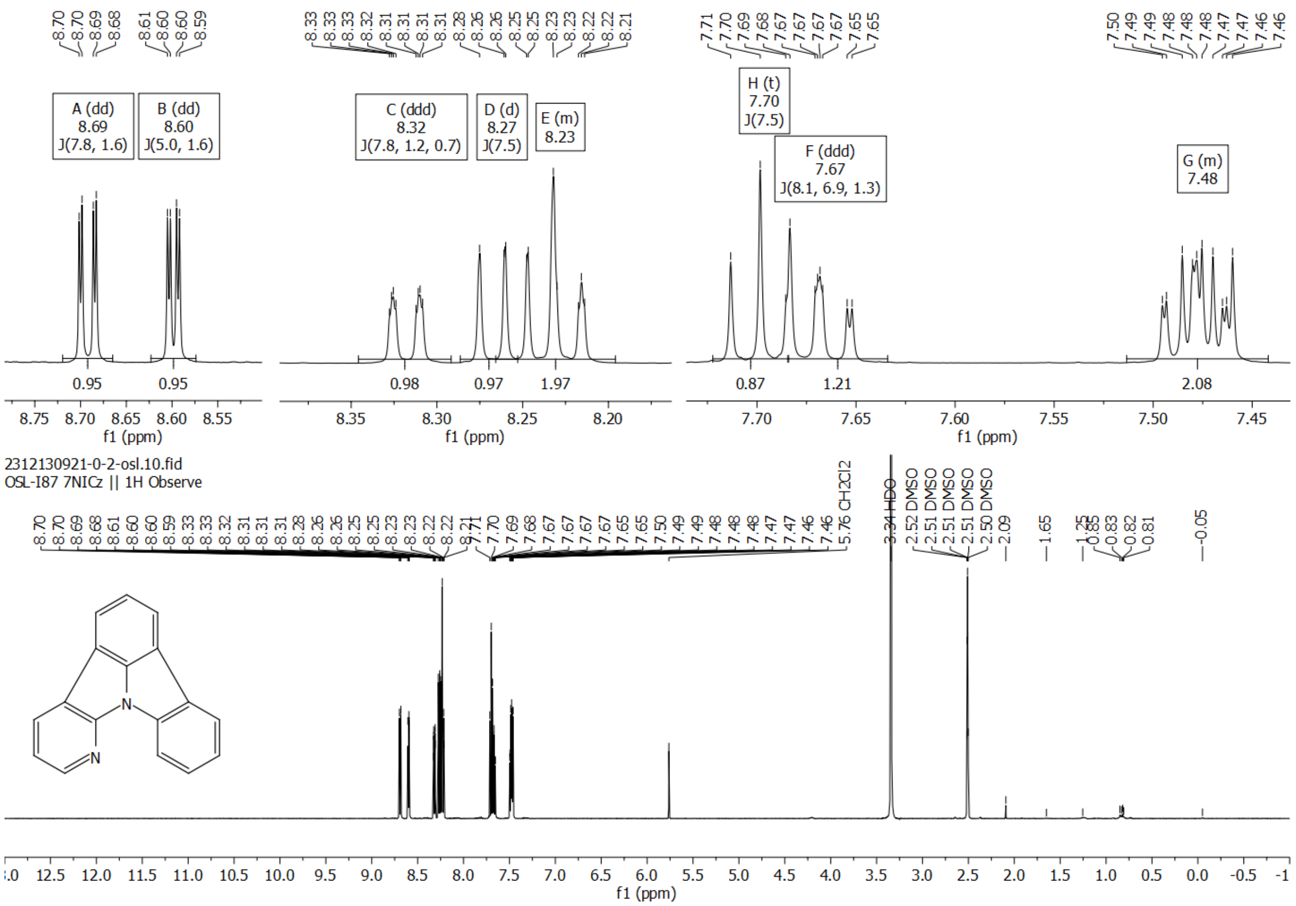

Figure **S27**. ^{1}H NMR of **7NICz** in d_6-DMSO.

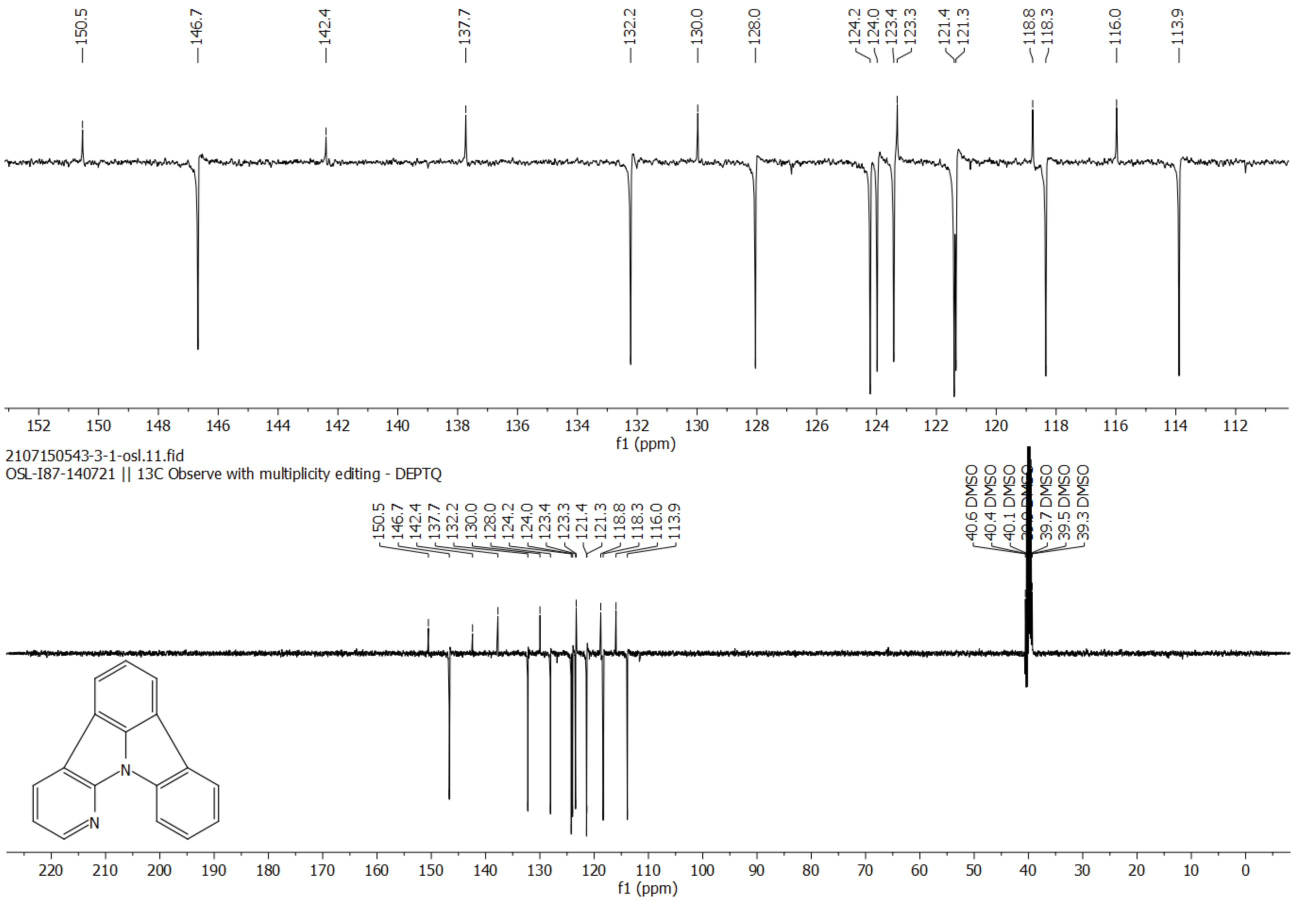

Figure **S28**. ^{13}C NMR of **7NICz** in d_6-DMSO.

<Sample Information>

Sample Name	: I87		
Sample ID	:		
Method Filename	: 65% Acetonitrile 35 Water 20 mins.lcm		
Batch Filename	: 01-09-2021.lcb		
Vial #	: 2-9	Sample Type	: Unknown
Injection Volume	: 5 uL		
Date Acquired	: 01/09/2021 13:54:46	Acquired by	: System Administrator
Date Processed	: 12/12/2023 15:06:38	Processed by	: System Administrator

<Chromatogram>

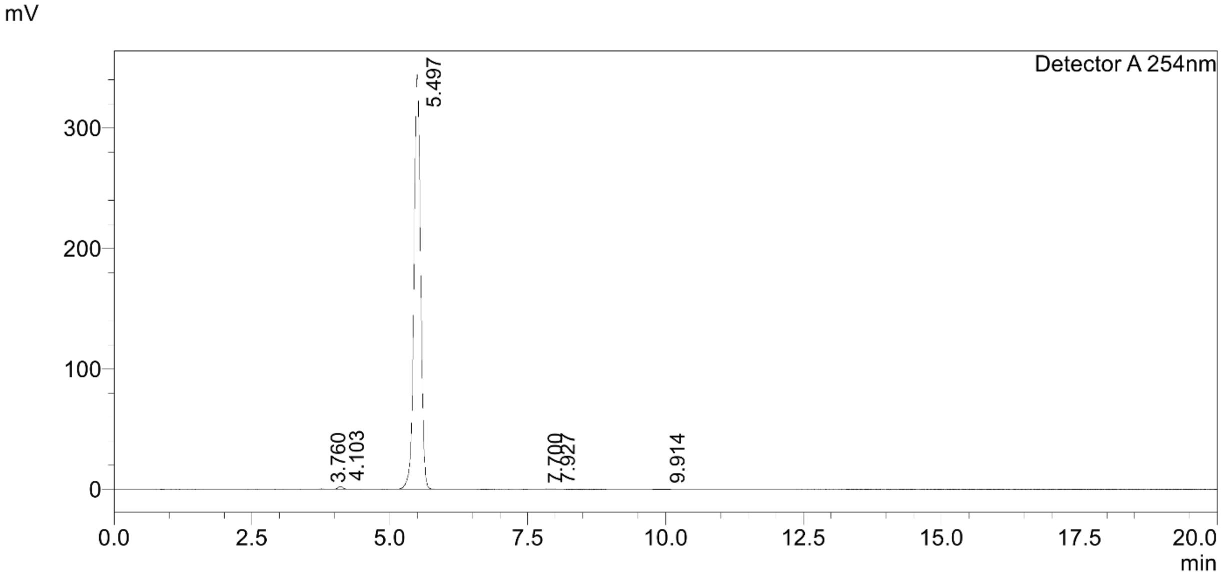

<Peak Table>

Detector A 254nm

Peak#	Ret. Time	Area	Height	Area%	Area/Height	Width at 5% Height
1	3.760	2942	479	0.101	6.137	0.203
2	4.103	16427	2464	0.563	6.667	0.226
3	5.497	2890348	344361	99.056	8.393	0.288
4	7.700	1648	187	0.056	8.814	--
5	7.927	5347	418	0.183	12.794	--
6	9.914	1191	94	0.041	12.641	0.350
Total		2917903	348003	100.000		

Figure **S29**. HPLC trace of **7NICz**.

Acquisition Parameter

Source Type	ESI	Ion Polarity	Positive	Set Nebulizer	0.4 Bar
Focus	Not active			Set Dry Heater	200 °C
Scan Begin	50 m/z	Set Capillary	3000 V	Set Dry Gas	6.0 l/min
Scan End	4000 m/z	Set End Plate Offset	-500 V	Set Divert Valve	Waste

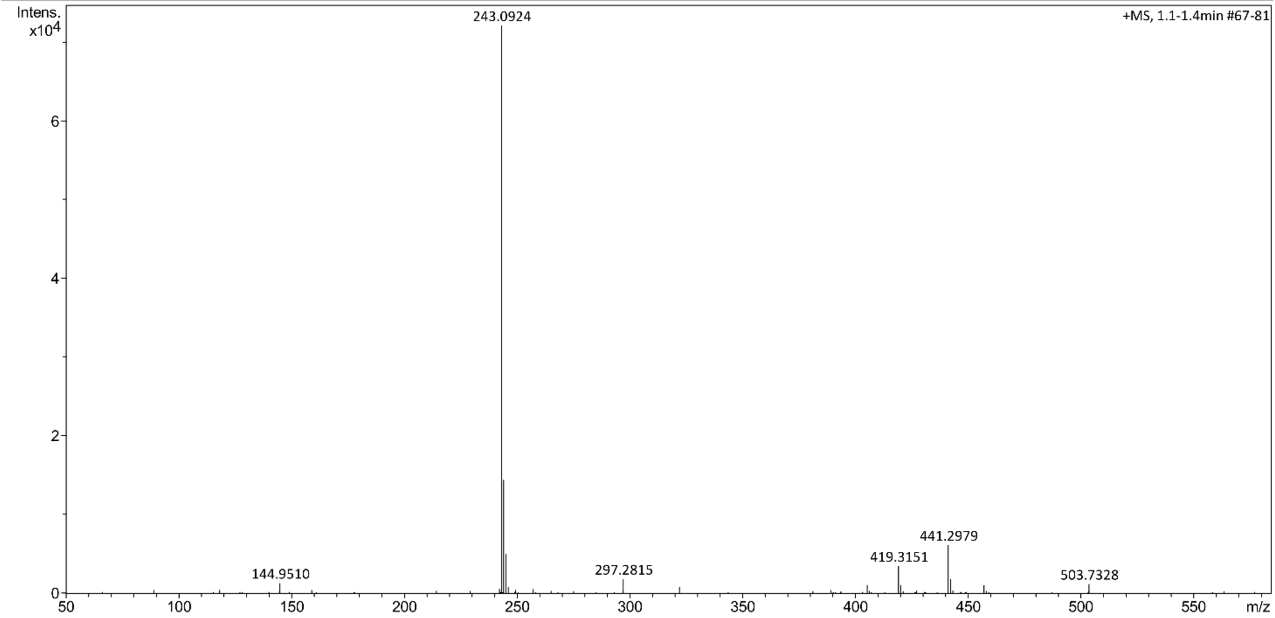

Acquisition Parameter

Source Type	ESI	Ion Polarity	Positive	Set Nebulizer	0.4 Bar
Focus	Not active			Set Dry Heater	200 °C
Scan Begin	50 m/z	Set Capillary	3000 V	Set Dry Gas	6.0 l/min
Scan End	4000 m/z	Set End Plate Offset	-500 V	Set Divert Valve	Waste

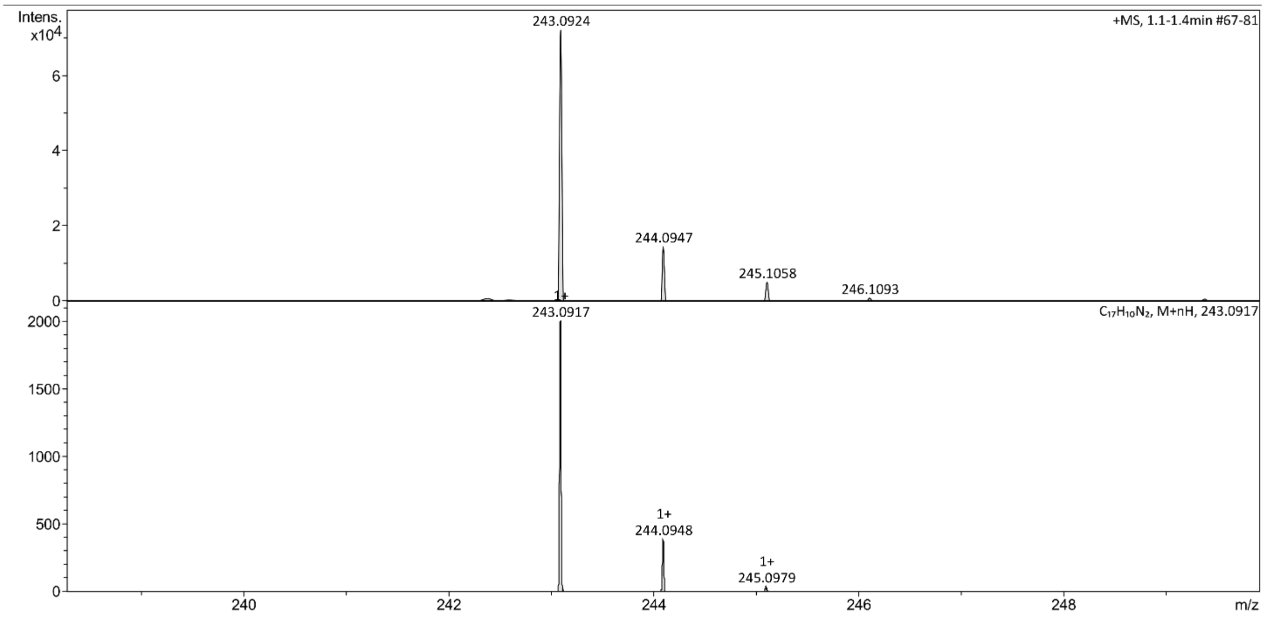

Figure **S30**. HRMS (ESI) trace of **7NICz**.

9-(4-Chloropyridin-3-yl)-9H-pyrido[3,4-b]indole (6,10PyCb)

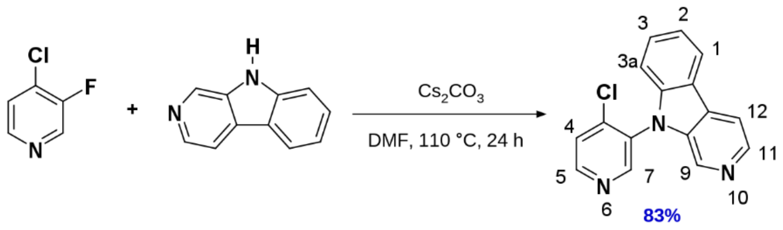

Protocol adapted from the literature.[35] Norharmane (0.25 g, 1.49 mmol, 1.0 equiv.) and Cs_2CO_3 (0.97 g, 2.97 mmol, 2.0 equiv.) were combined and degassed by three successive vacuum/nitrogen cycles. Under a flow of N_2, 4-chloro-3-fluoropyridine (0.29 mL, 2.97 mmol, 2.0 equiv.) and then DMF (3.0 mL) were added *via* syringe, forming a dark red solution. The reaction mixture was then heated to 110 °C for approximately 24 h, after which a cloudy brown suspension had formed. After cooling to room-temperature, the reaction crude was poured into brine and then extracted from EtOAc/brine before the organic fractions were combined and dried over Na_2SO_4. The EtOAc solution was then directly evaporated onto silica and the crude was purified by flash chromatography in EtOAc. The fractions were combined and evaporated to **yield** a yellow-orange oil (0.35, 83%), which was used without further purification. **^{1}H NMR (400 MHz; $(CD_3)_2SO$) δ_H (ppm)**: 8.98 (d, J = 0.5 Hz, 1H, H_7), 8.84 (d, J = 5.5 Hz, 1H, H_5), 8.56 (d, J = 1.1 Hz, 1H, H_9), 8.52 (d, J = 5.2 Hz, 1H, H_{12}), 8.41 (ddd, J = 7.9, 1.2, 0.8 Hz, 1H, H_1), 8.29 (dd, J = 5.2, 1.1 Hz, 1H, H_{11}), 8.00 (dd, J = 5.5, 0.3 Hz, 1H, H_4), 7.61 (ddd, J = 8.3, 7.2, 1.2 Hz, 1H, H_3), 7.42 (ddd, J = 7.9, 7.2, 0.9 Hz, 1H, H_2), and 7.23 (ddd, J = 8.3, 0.9, 0.8 Hz, 1H, H_{3a}). **^{13}C{^{1}H} NMR (101 MHz; $(CD_3)_2SO$) δ_C (ppm)**: 151.6 (CH), 151.6 (CH), 142.1 (C_{quat}), 141.1 (C_{quat}), 140.3 (CH), 136.5 (C_{quat}), 132.9 (CH), 130.5 (C_{quat}), 129.2 (CH), 128.5 (C_{quat}), 125.9 (CH), 122.4 (CH), 121.2 (CH), 121.2 (C_{quat}), 115.1 (CH), and 110.5 (CH). Characterization data matches those in the literature..[35]

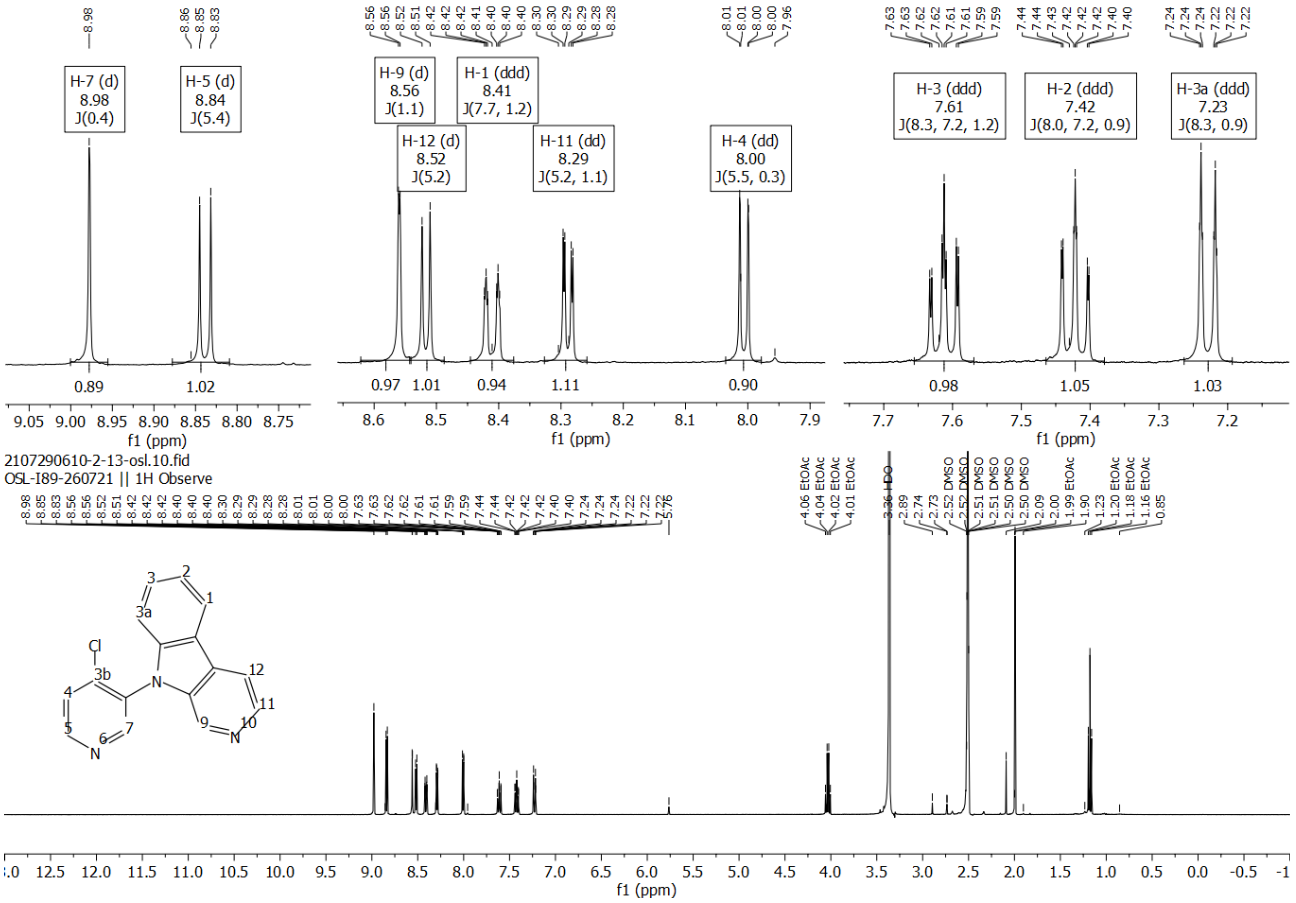

Figure **S31**. ^{1}H NMR of **Chloro-6,10PyCb** in d_6-DMSO.

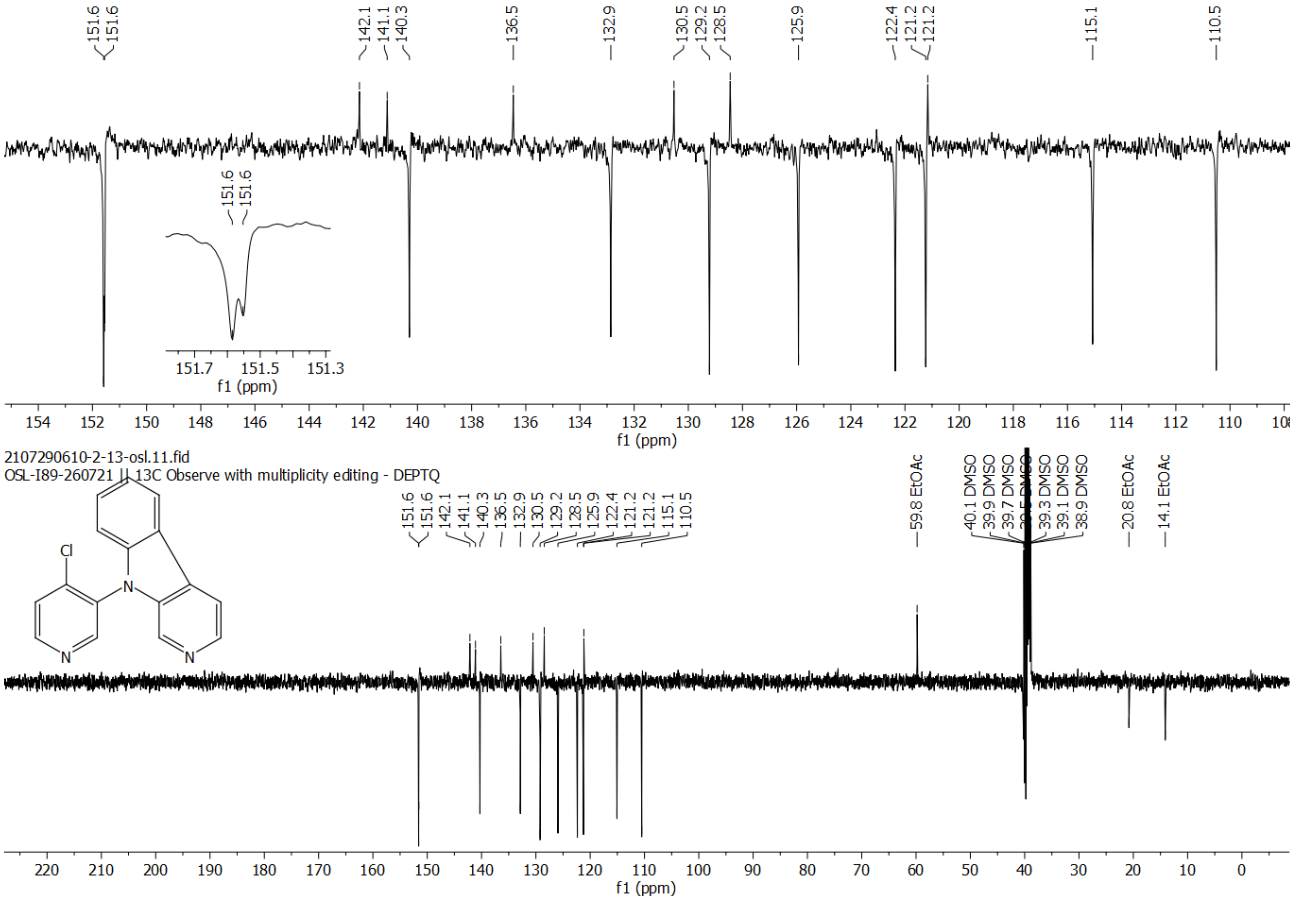

Figure **S32**. ^{13}C NMR of **Chloro-6,10PyCb** in d_6-DMSO.

Pyrido[3,4- b]pyrido[4',3':4,5]pyrrolo[3,2,1-hi]indole (6,10NICz)

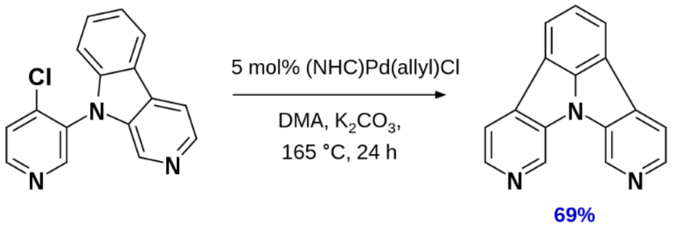

Protocol adapted from the literature.[35] 9-(4-Chloropyridin-3-yl)-9H-pyrido[3,4-b]indole (0.35 g, 1.23 mmol, 1.0 equiv.) was dissolved in EtOAc and washed into a reaction vessel before the solvent was removed. Allyl[1,3-bis(2,6-diisopropyl-phenyl)imidazol-2-ylidene]chloropalladium(II) (35 mg, 0.06 mmol, 5 mol%), and K_2CO_3 (0.34 g, 2.47 mmol, 2.0 equiv.) were combined and degassed by three successive vacuum/nitrogen cycles. Under a flow of N_2, DMA (13.0 mL) was added *via* syringe to form a yellow solution with a white suspension. The reaction mixture was then heated to 165 °C for approximately 24 h, after which it had turned dark red. After cooling to room-temperature, the reaction crude was filtered through celite with EtOAc to remove excess Pd before being poured into brine to form a milky white precipitate. A liquid-liquid extraction was then performed with EtOAc/Brine and the organic fractions were combined and dried over Na_2SO_4. The solvent was then evaporated to reveal the crude as white-brown crystals. The crude was next evaporated onto silica and purified by flash column chromatography in 10:35:55 MeOH:EtOAc:hexane. The product was obtained as the second spot and was evaporated to form yellow crystals (0.21 g). The crystals were partially dissolved in boiling 50% EtOAc:Hexane (*ca.* 12 mL) before being allowed to cool in a freezer overnight. The solution was filtered to **yield** the product as fluffy, pale-yellow crystals (0.18 g, 69%). **R_f**: 0.35 (10 : 50 : 40 MeOH : EtOAc : hexane). **Mp**: 235 - 238 °C. Purity by **HPLC**: 99.7%. **^{1}H NMR (400 MHz; $(CD_3)_2SO$) δ_H (ppm)**: 9.74 (d, J = 1.1 Hz, 2H), 8.68 (d, J = 5.1 Hz, 2H), 8.49 (d, J = 7.5 Hz, 2H), 8.34 (dd, J = 5.1, 1.0 Hz, 2H), and 7.81 (t, J = 7.5 Hz, 1H).**^{13}C{^{1}H} NMR (101 MHz; $(CD_3)_2SO$) δ_C (ppm)**: 144.2 (C_{quat}), 143.4 (CH), 136.0 (CH), 135.5 (C_{quat}), 134.5 (C_{quat}), 124.8, (CH), 124.7 (CH), 118.5 (CH), and 117.3 (C_{quat}).. **HR-MS[M+H]$^+$**: Calculated: ($C_{16}H_{10}N_3$): 244.0869; Found: 244.0863. Characterization data matches those in the literature.[35]

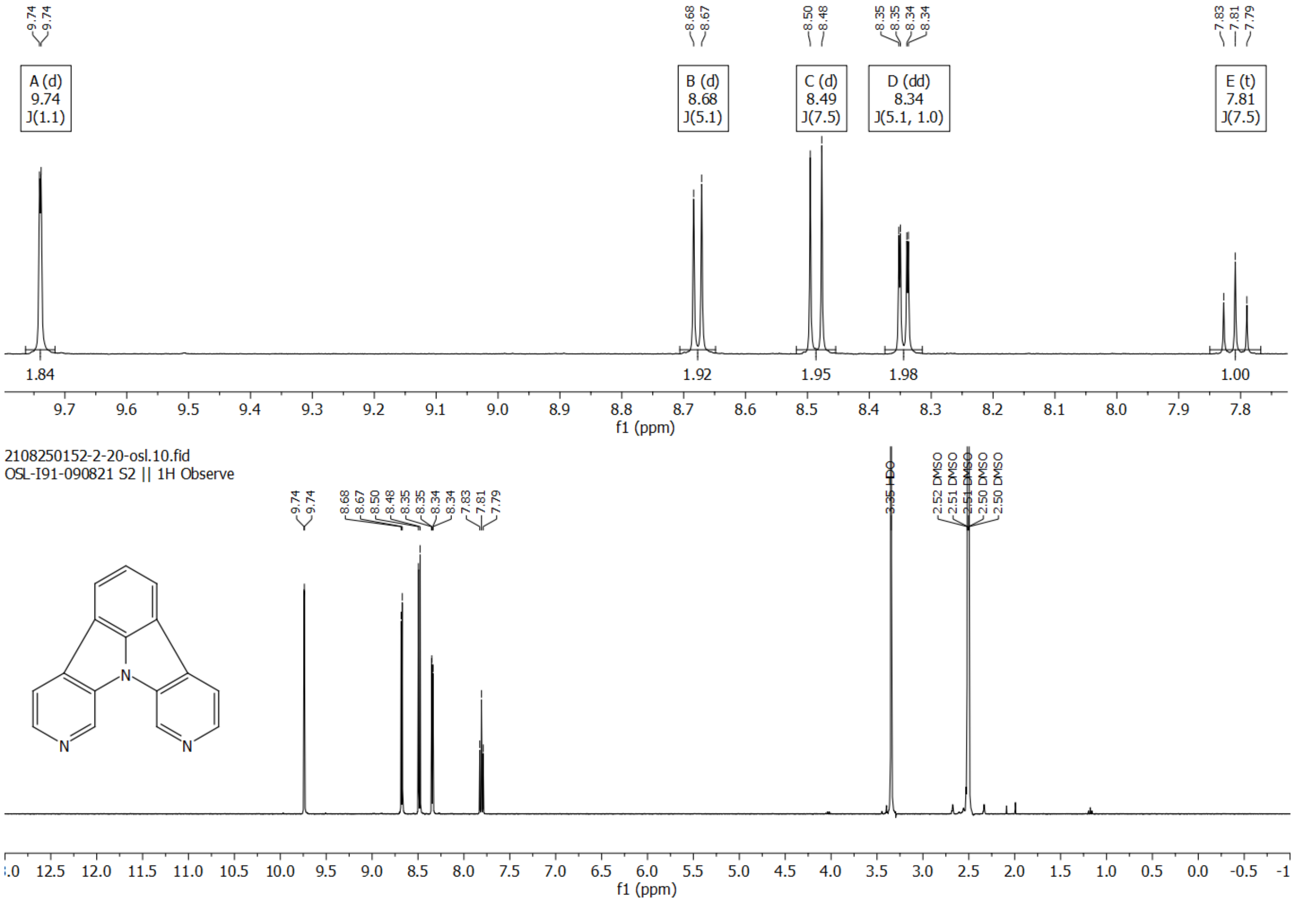

Figure **S33**. ^{1}H NMR of **6,10NICz** in d_6-DMSO.

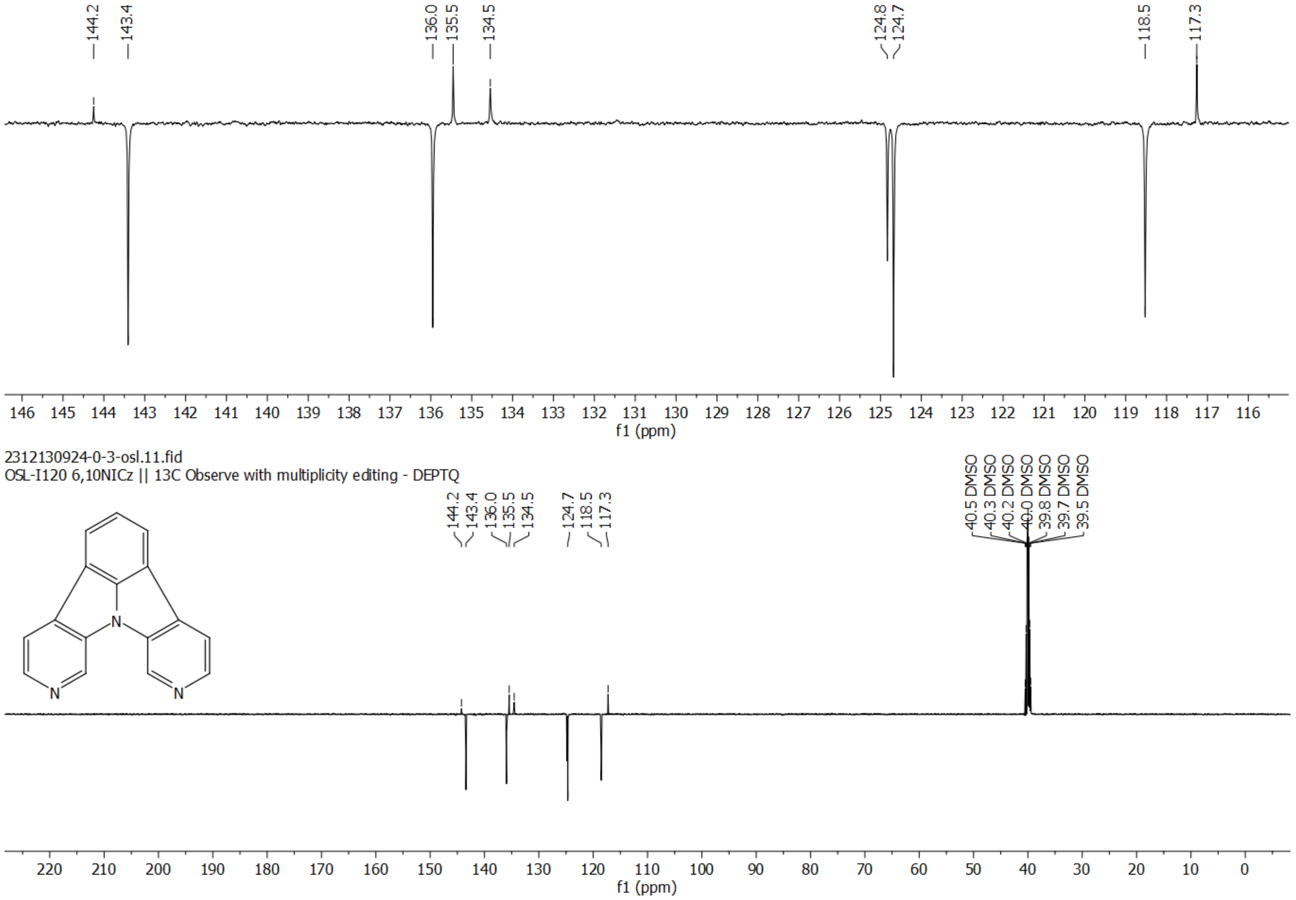

Figure **S34**. ^{13}C NMR of **6,10NICz** in d_6-DMSO.

HPLC Trace Report18Dec2023

<Sample Information>

Sample Name	: 6,10NICz I120		
Sample ID	:		
Method Filename	: 50% THF 50% water 0.6 mlmin 20 mins.lcm		
Batch Filename	: Batch.lcb		
Vial #	: 2-29	Sample Type	: Unknown
Injection Volume	: 1 uL		
Date Acquired	: 18/12/2023 14:49:27	Acquired by	: System Administrator
Date Processed	: 18/12/2023 15:21:44	Processed by	: System Administrator

<Chromatogram>

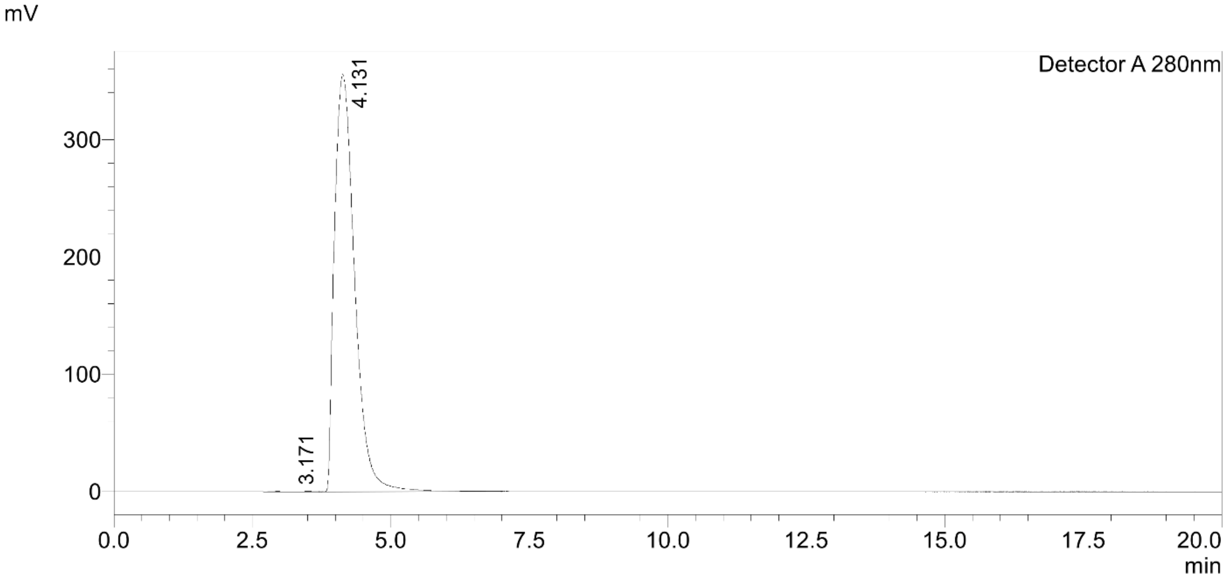

<Peak Table>

Detector A 280nm

Peak#	Ret. Time	Area	Height	Area%	Area/Height	Width at 5% Height
1	3.171	28303	941	0.313	30.091	0.855
2	4.131	9003632	355566	99.687	25.322	0.805
Total		9031934	356507	100.000		

Figure **S35**. HPLC trace of **6,10NICz**.

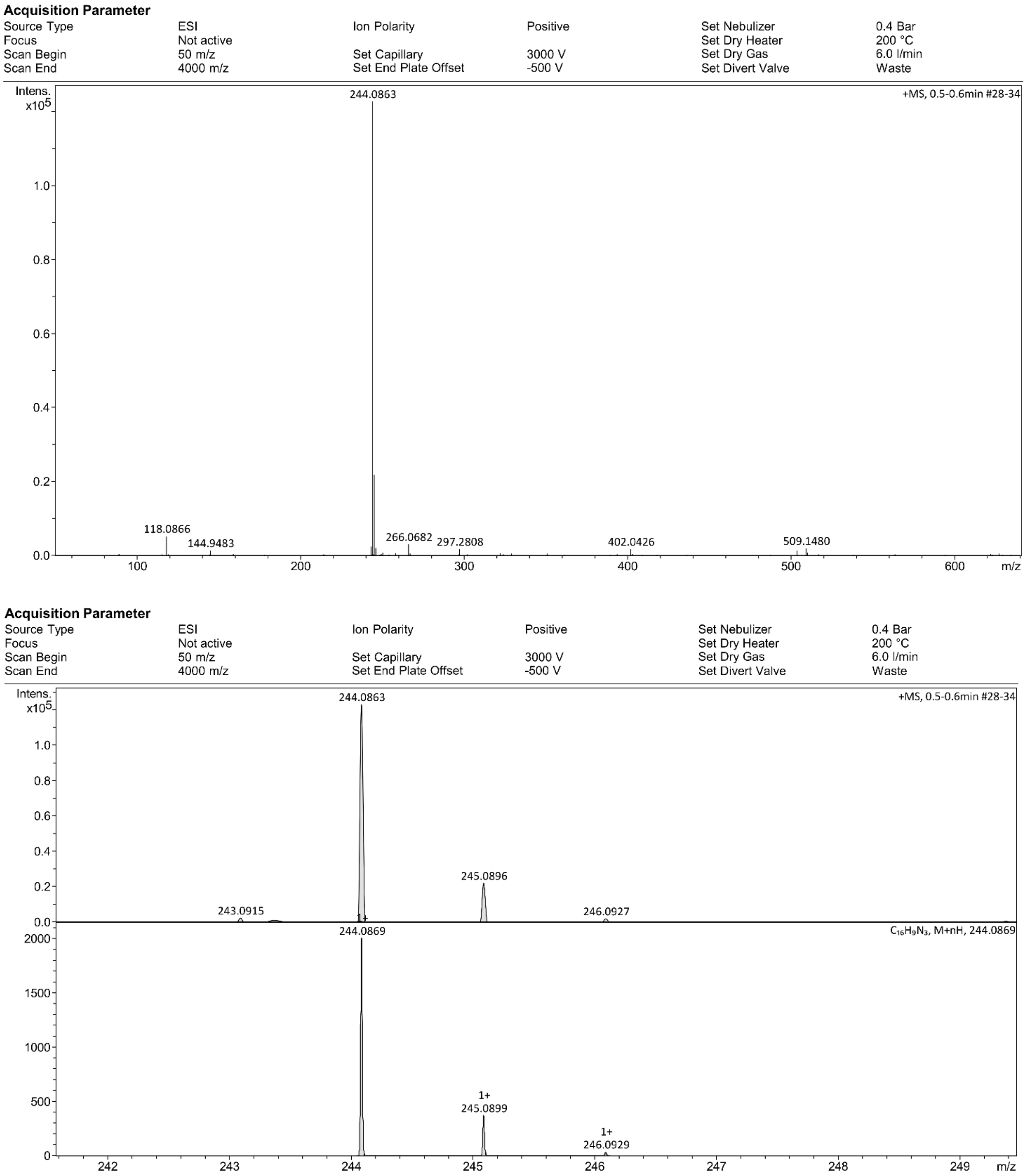

Figure **S36**. HRMS trace of **6,10NICz**.

Crystal Structures

X-ray diffraction data for compounds **4NICz**, **5NICz**, **6NICz**, **7NICz**, and **6,10NICz** were collected at 100 K using a Rigaku MM-007HF High Brilliance RA generator/confocal optics with XtaLAB P200 diffractometer [Cu Kα radiation (λ = 1.54187 Å)]. Data for all compounds analysed were

collected (using a calculated strategy) and processed (including correction for Lorentz, polarization and absorption) using CrysAlisPro.[41] Structures were solved by either direct methods (SHELXS[42]), for **4NICz**, or dual-space methods (SHELXT[43]), for all other structures, and refined by full-matrix least-squares against F^2 (SHELXL-2019/3[44]). Non-hydrogen atoms were refined anisotropically, and hydrogen atoms were refined using a riding model. The structure of **4NICz** contained channels of disordered ethyl acetate solvates along the crystallographic *b* axis which were modelled using two overlapping fragments with restraints to bond distances, angles and thermal motions. Two component twinning around [1 0 0.164 0 -1 0 0 0 -1] of **5NICz** was located using PLATON.[45] In one of the independent molecules of **6NICz** and in **7NICz** the location of the heterocyclic nitrogen was disordered with its opposite carbon in ratios of 4:1 and 11:9 respectively. This disorder was modelled with constraints on the shared sites. All other calculations were performed using the Olex2[46] interface. Selected crystallographic data are presented in Table **S1**. CCDC 2337758-2337762 contains the supplementary crystallographic data for this paper. These data can be obtained free of charge from The Cambridge Crystallographic Data Centre via www.ccdc.cam.ac.uk/structures.

Table **S1**. Selected crystallographic data.

	**4NICz**	**5NICz**	**6NICz**	**7NICz**	**6,10NICz**
**formula**	$C_{19}H_{14}N_2O$	$C_{17}H_{10}N_2$	$C_{17}H_{10}N_2$	$C_{17}H_{10}N_2$	$C_{16}H_9N_3$
**fw**	286.32	242.27	242.27	242.27	243.26
**crystal description**	Colourless needle	Colourless needle	Yellow needle	Colourless plate	Yellow needle
**crystal size [mm^3]**	0.24 × 0.02 × 0.01	0.25 × 0.015 × 0.005	0.51 × 0.03 × 0.01	0.12 × 0.04 × 0.02	0.48 × 0.01 × 0.01
**space group**	$P2_1/c$	$P2_1/c$	$P2_1/c$	$P2_1/n$	$P2_12_12_1$
***a* [Å]**	18.8259(10)	14.4657(4)	27.5407(5)	5.17603(9)	3.81280(15)
***b* [Å]**	3.8626(2)	10.9278(4)	5.17412(13)	14.7691(3)	15.0447(6)
***c* [Å]**	20.6192(13)	7.2829(3)	16.2455(3)	14.8972(3)	19.3445(10)
***β* [°]**	113.744(7)	92.362(3)	91.8492(16)	93.6727(16)	
**vol [Å]^3**	1372.45(15)	1150.30(6)	2313.76(8)	1136.48(4)	1109.65(9)
Z	4	4	8	4	4
***ρ* (calc) [g/cm^3]**	1.386	1.399	1.391	1.416	1.456
***μ* [mm^{-1}]**	0.690	0.654	0.650	0.662	0.708
**F(000)**	600.0	504.0	1008.0	504.0	504.0
**reflections collected**	13871	19900	39331	10997	11022
**independent reflections (R_{int})**	2797 (0.0560)	2341 (0.0807)	4774 (0.0827)	2305 (0.0512)	2248 (0.1186)
**parameters, restraints**	283, 151	173, 0	345, 0	173, 0	172, 0
**GoF on F^2**	1.069	1.134	1.097	1.070	1.085
**R_1 [$I > 2\sigma(I)$]s**	0.0655	0.0669	0.0545	0.0397	0.0604
**wR_2 (all data)**	0.2141	0.1858	0.1562	0.1029	0.1754
**largest diff. peak/hole [e/Å^3]**	0.38/-0.25	0.29/-0.29	0.20/-0.24	0.20/-0.18	0.26/-0.32

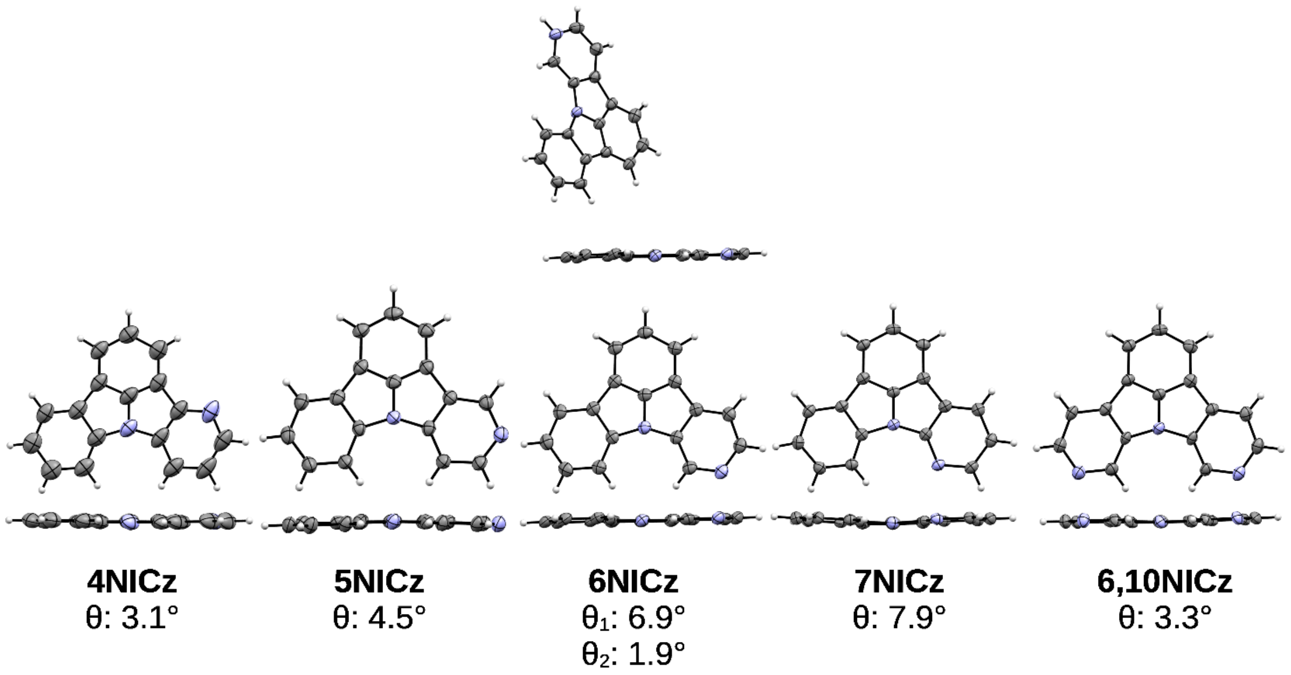

Figure **S37**. Crystal structures of the five emitters, shows as thermal ellipsoids at 50% probability. θ is the mean absolute dihedral angle between atoms C_7, C_{7a}, N_8, C_{8a}, and C_{7a}, N_8, C_{8a}, C_9. The ethyl acetate solvent in **4NICz** has been omitted for clarity. The structure of **6NICz** contained two molecules of the emitter with different torsion (top image), the θ for each has been reported.

Crystals of all five emitters were grown by layering EtOAc solutions of each emitter with hexane. Each emitter adopted almost completely planar geometry, as predicted from computations (Figure **S37**), but with a very slight dihedral angle across N_8. The greatest deviation from planarity was observed in **7NICz** with $\theta = 7.9°$. The structure of **6NICz** contained two molecules of the same emitter, one located approximately perpendicular to the other, where one of the molecules was noticeably more bent than the other ($\theta_1 = 1.9°$, $\theta_2 = 6.9$).

Additional Computational and Photophysical Data

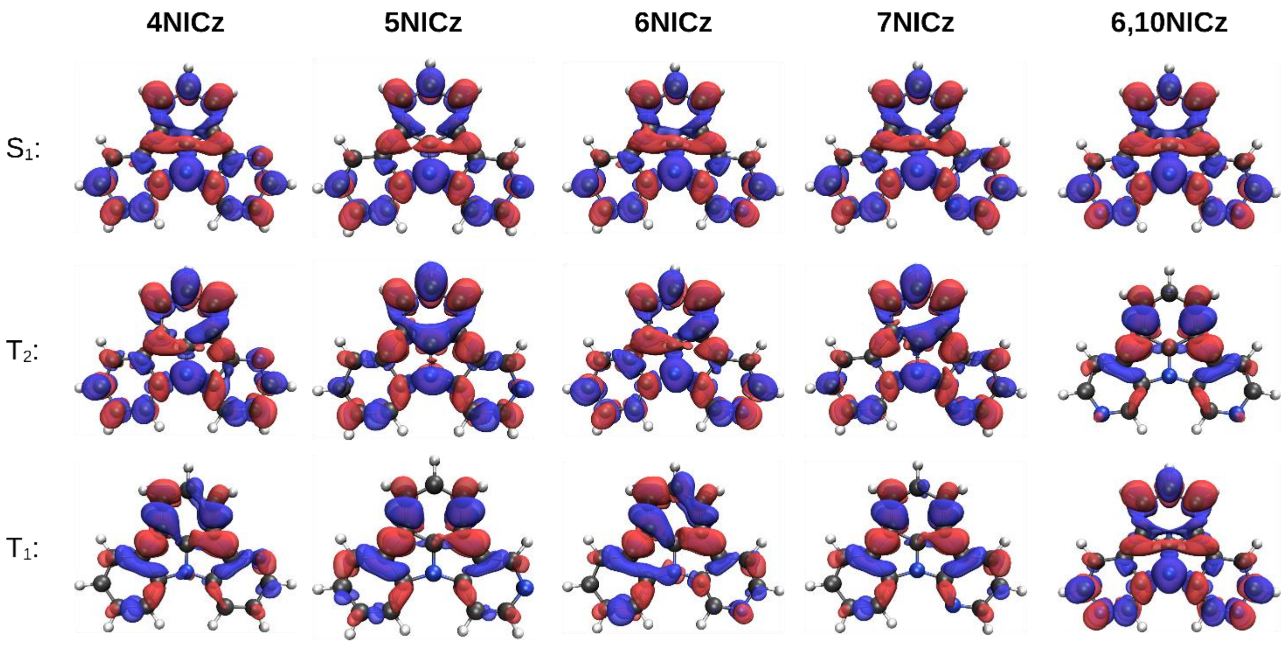

Figure **S38**. Difference density plots of the S_1, T_2 and T_1 excited states compared to the ground state. All calculations were performed using the RI-SCS-ADC(2)/cc-pVDZ level of theory, in the gas phase. The red region of the difference density plots corresponds to an increase in electron density in the excited state; the blue to a decrease.

Table **S2.** Complete excited state calculation results.

**Emitter**	**S_1 / eV**	**f_{S1}[a]**	**S_1 HOMO → LUMO[b] / %**	**S_2 / eV**	**f_{S2}**	**f_{S1}/f_{S2}[c]**	**T_1 / eV**	**T_2 / eV**
**4NICz**	3.80	0.07	78	4.32	0.14	0.5	3.45	3.64
**5NICz**	3.91	0.14	72	4.42	0.06	2.3	3.48	3.69
**6NICz**	3.69	0.13	86	4.34	0.09	1.4	3.46	3.52
**7NICz**	3.84	0.08	74	4.34	0.11	0.7	3.46	3.67
**6,10NICz**	3.62	0.15	88	4.37	0.07	2.1	3.42	3.53

[a]oscillator strength of the $S_0 \rightarrow S_1$ transition. [b]percentage contribution to the S_1 excited state from the HOMO → LUMO transition. [c]ratio of f_{S1} to f_{S2}. All calculations were performed using the RI-SCS-ADC(2)/cc-pVDZ level of theory, in the gas phase.

Table **S3**. Complete absorption properties in dilute solution

**Emitter**	**$\lambda_{abs,1}$ / nm (shoulder)**	**$\varepsilon_{abs,1}$ / 10^3 $M^{-1}cm^{-1}$ (shoulder)**	**$\lambda_{abs,2}$ / nm (shoulder)**	**$\varepsilon_{abs,2}$ / 10^3 $M^{-1}cm^{-1}$ (shoulder)**	**$\varepsilon_{abs,1}$/ $\varepsilon_{abs,2}$**	**$\lambda_{abs,3}$ / nm (shoulder)**	**$\varepsilon_{abs,3}$ / $10^3M^{-1}cm^{-1}$ (shoulder)**

**4NICz**	360 (353)	8.0 (7.9)	322 (310)	13.8 (12.8)	0.6	297 (291)	35.3 (33.2)
**5NICz**	352 (340)	22.9 (9.7)	312	6.5	3.5	< 285 nm	-
**6NICz**	374 (359)	14.0 (8.9)	323 (311)	7.7 (7.2)	1.8	< 285 nm	-
**7NICz**	359 (345)	8.2 (6.0)	318	8.7	0.9	297 (290)	26.8 (26.8)
**6,10NICz**	382 (365)	9.5 (5.4)	319 (307)	3.8 (3.3)	2.5	< 285 nm	-

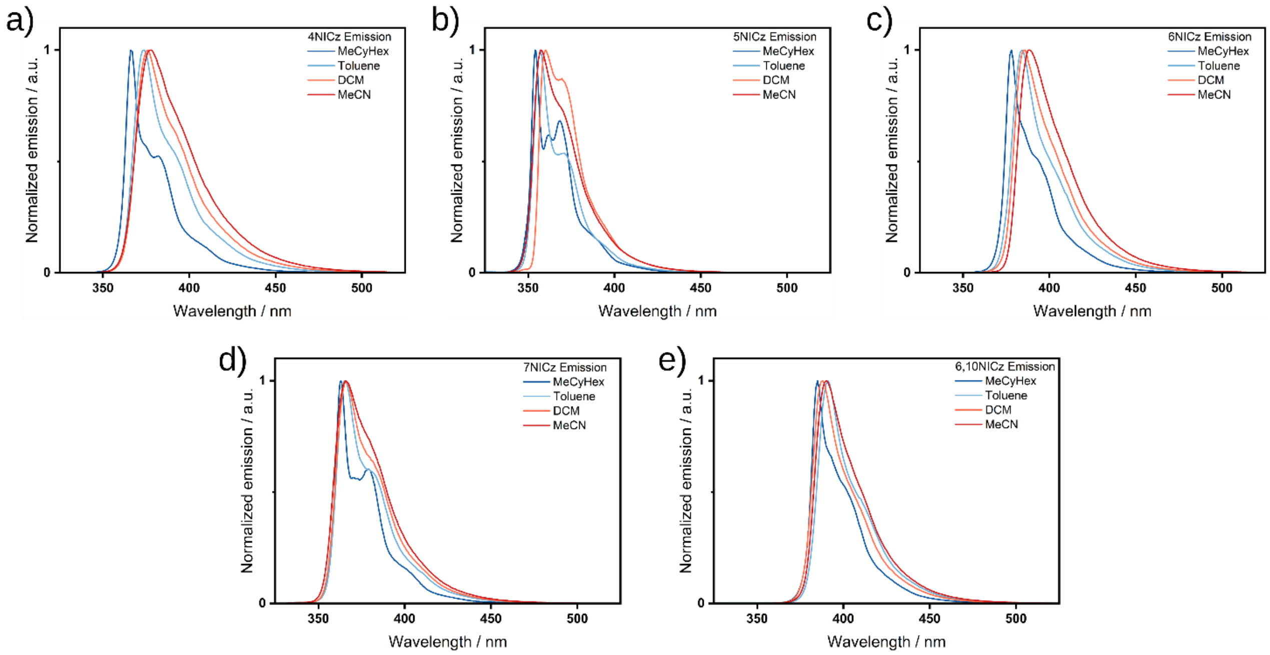

Figure **S39**. Steady-state PL spectra of a) **4NICz**, b) **5NICz**, c) **6NICz**, d) **7NICz**, and e) **6,10NICz** in methylcyclohexane (MeCyHex) Toluene, DCM and MeCN. λ_{exc} = 297 nm (**4NICz**), 287 nm (**5NICz**), 325 nm (**6NICz**), 297 nm (**7NICz**) and 286 nm (**6,10NICz**).

Table **S4**. Table of the full fitting parameters of the S_1 decay in 10 wt% PMMA films.

**Emitter**	**$\tau_{p,1}$ / ns (%)**	**$\tau_{p,2}$ / ns (%)**	**$\tau_{p,3}$ / ns (%)**	**τ_p / ns**	**$\tau_{d,1}$ / ms (%)**	**$\tau_{d,2}$ / ms (%)**	**τ_d / ms**
**4NICz**	3.3 (27)	12.2 (73)	-	9.8	-	-	-
**5NICz**	0.5 (18)	2.1 (59)	8.5 (23)	3.3	-	-	-
**6NICz**	0.9 (13)	3.4 (58)	8.8 (30)	4.7	-	-	-
**7NICz**	2.8 (20)	12.5 (80)	-	10.6	-	-	-
**6,10NICz**	0.6 (7)	2.8 (62)	6.2 (30)	1.7	2.5 (25)	15.0 (75)	11.9

Table **S5**. Table of the full fitting parameters of the S_1 decay in 1 wt% PMMA films.

Emitter	$\tau_{p,1}$ / ns (%)	$\tau_{p,2}$ / ns (%)	τ_p / ns	$\tau_{d,1}$ / ms (%)	$\tau_{d,2}$ / ms (%)	τ_d / ms
**4NICz**	-	-	-[a]	-	-	-
**5NICz**	-	-	-[a]	-	-	-
**6NICz**	1.6 (36)	4.7 (64)	3.6	21.4 (2)	501.1 (98)	489.2
**7NICz**	-	-	-[a]	-	-	-
**6,10NICz**	0.6 (18)	3.4 (82)	2.9	9.7 (33)	45.8 (67)	34.0

[a] not recordable due to the peak of emission being higher in energy than the excitation source.

Table **S6**. Table of the full fitting parameters of the T_1 decay in 1 wt% PMMA films.

Emitter	$\tau_{p,1}$ / ns (%)	$\tau_{p,2}$ / ns (%)	τ_p / ns	$\tau_{d,1}$ / s (%)	$\tau_{d,2}$ / s (%)	τ_d / s
**4NICz**	2.1 (27)	8.7 (73)	6.9	-	-	-[b]
**5NICz**	1.0 (32)	9.5 (68)	6.8	1.5	-	1.5
**6NICz**	3.5 (20)	11.3 (80)	9.8	0.04 (10)	0.9 (90)	0.8
**7NICz**	2.2 (21)	11.3 (79)	9.4	0.1 (7)	2.1 (93)	1.9
**6,10NICz**	2.8 (55)	7.2 (45)	4.8	-	-	-

[b] not recordable in the maximum time window of the spectrometer.

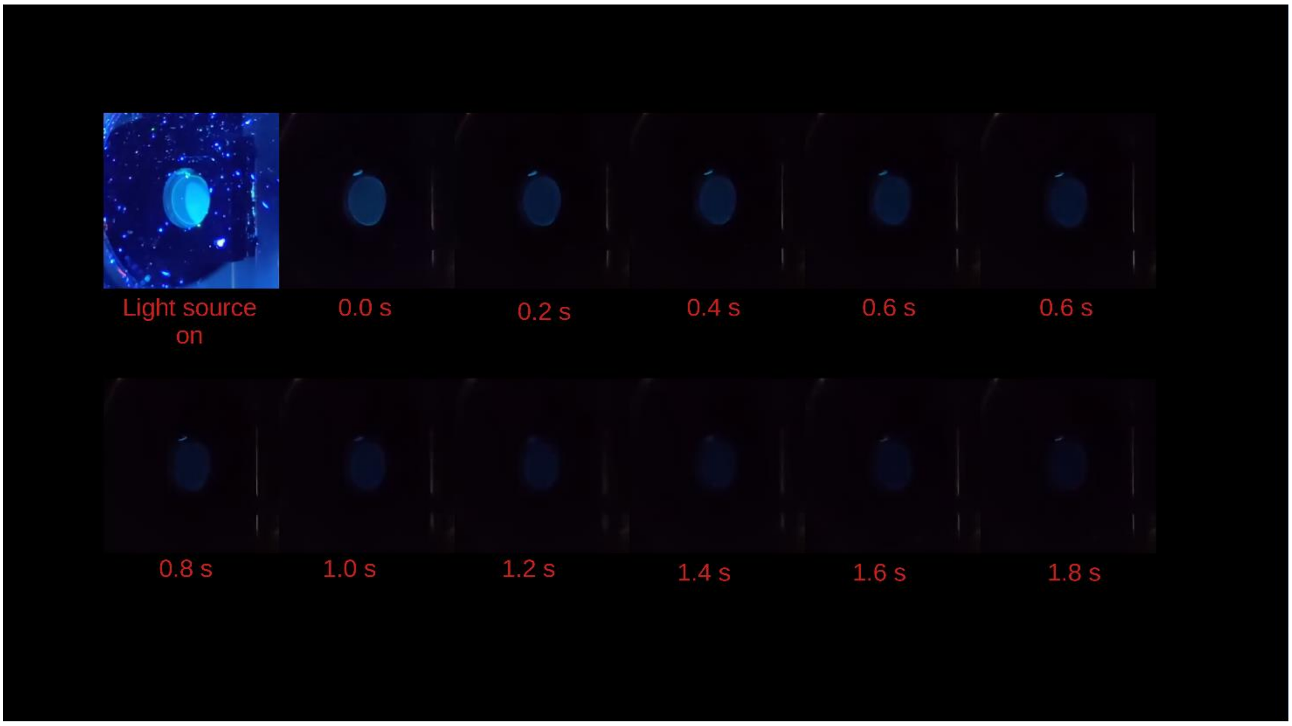

Figure **S40**. Still frames taken from a video of the room-temperature phosphorescence of **4NICz** in 1 wt% doped film in PMMA. The video was recorded using a standard smart phone camera at 30 frames-per-second (fps). Frames were extracted every 6 frames (0.2 s). Times are referenced to the first frame without external illumination. The film was excited by a hand-held UV torch.

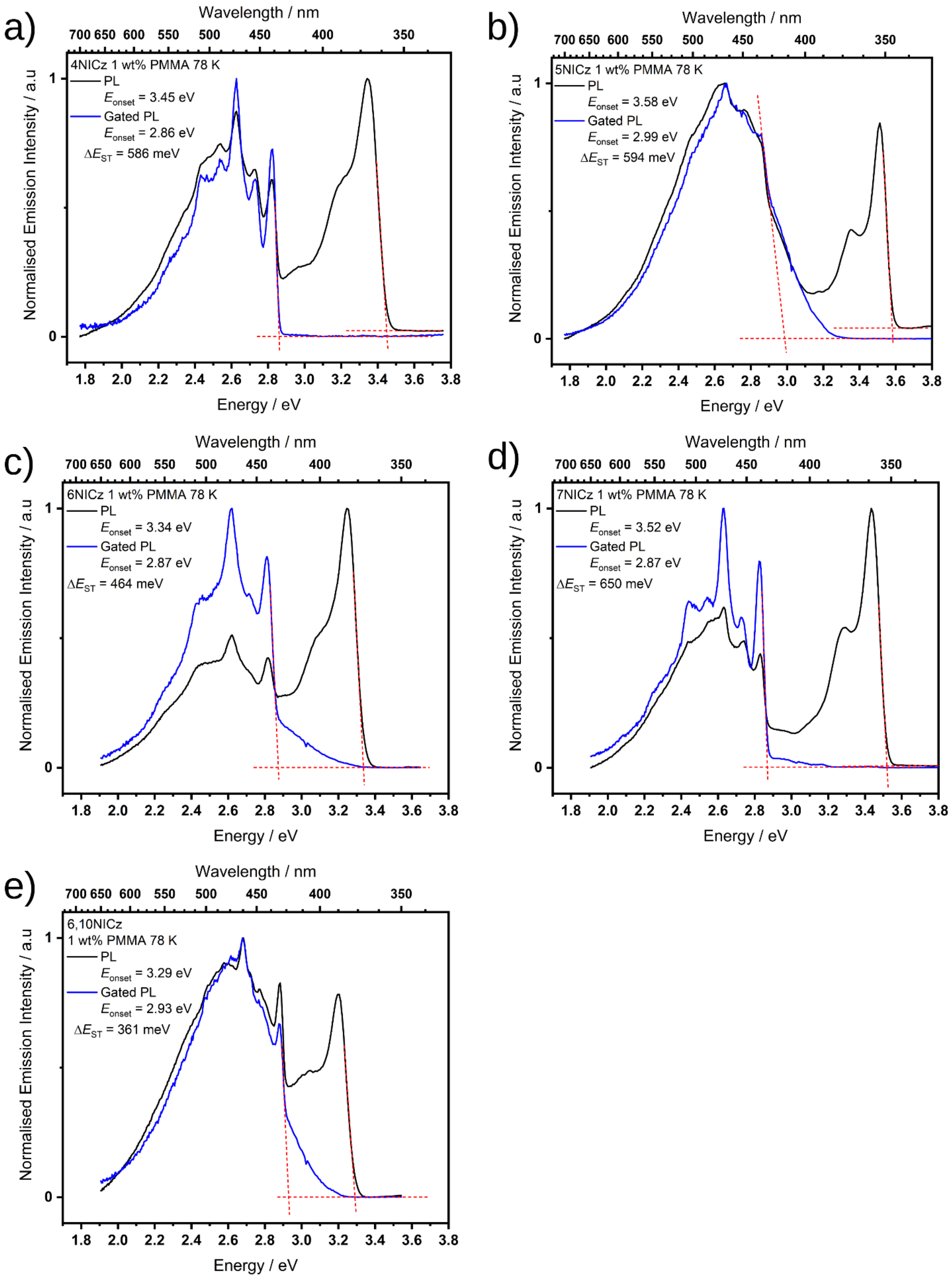

Figure **S41**. Steady-state PL and time-gated PL (1 – 10 ms) spectra of a) **4NICz**, b) **5NICz**, c) **6NICz**, d) **7NICz**, and e) **6,10NICz** in 1 wt% doped films in PMMA films at 77 K under vacuum.

λ_{exc} = 295 nm (**4NICz**), 280 nm (**5NICz**), 283 nm (**6NICz**), 294 nm (**7NICz**) and 278 nm (**6,10NICz**).

Table **S7**. Energies of the singlet and triplet excited states.

	Experimental[a]			Computational[b]		
**Emitter**	**E_{S1} / eV**	**E_{T1} / eV**	**ΔE_{ST} / eV**	**E_{S1} / eV**	**E_{T1} / eV**	**ΔE_{ST} / eV**
**4NICz**	3.45	2.86	0.59	3.80	3.45	0.35
**5NICz**	3.58	2.99	0.59	3.91	3.48	0.43
**6NICz**	3.34	2.87	0.46	3.69	3.46	0.24
**7NICz**	3.52	2.87	0.65	3.84	3.46	0.37
**6,10NICz**	3.29	2.93	0.36	3.62	3.42	0.20

[a]determined from the onset of steady state emission (S_1) and 1-10 ms time-gated emission (T_1) at 77 K in 1 wt% doped films in PMMA. [b]performed at the RI-SCS-ADC(2)/cc-pVDZ level of theory.

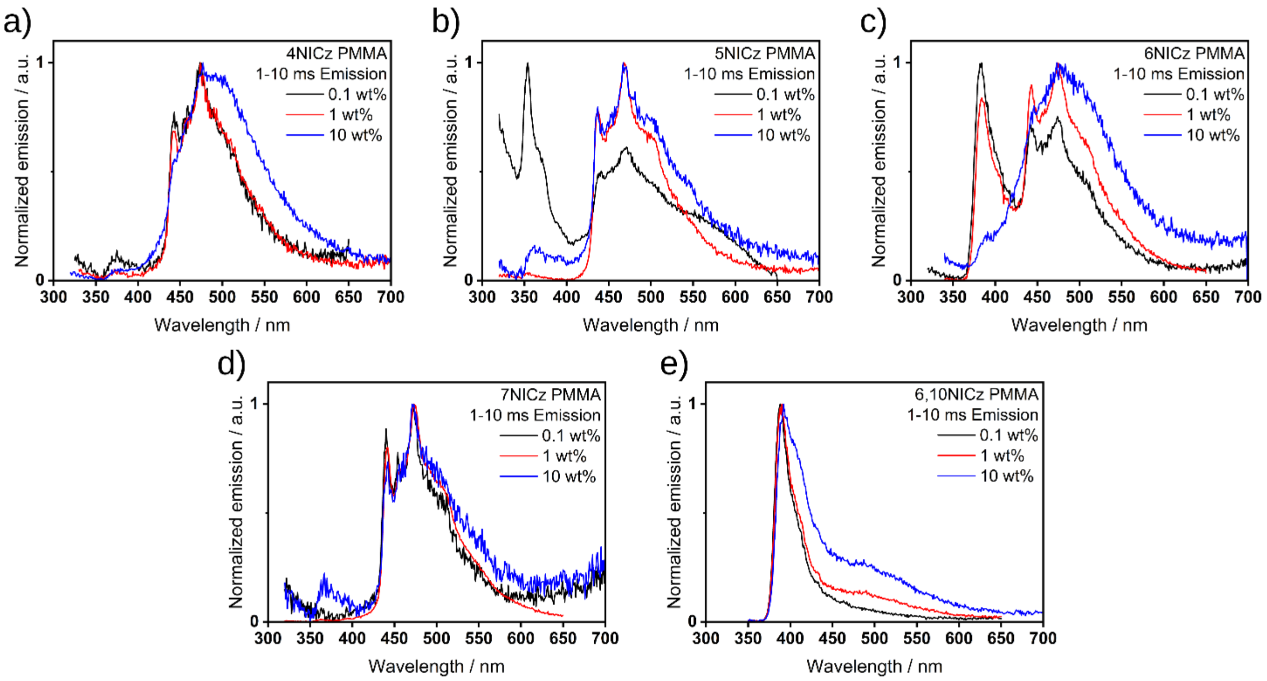

Figure **S42**. The change in 1-10 ms gated PL spectra of a) **4NICz**, b) **5NICz**, c) **6NICz**, d) **7NICz**, and e) **6,10NICz** in doped films in PMMA as a function of concentration. λ_{exc} = 295 nm (**4NICz**), 280 nm (**5NICz**), 283 nm (**6NICz**), 294 nm (**7NICz**) and 278 nm (**6,10NICz**).

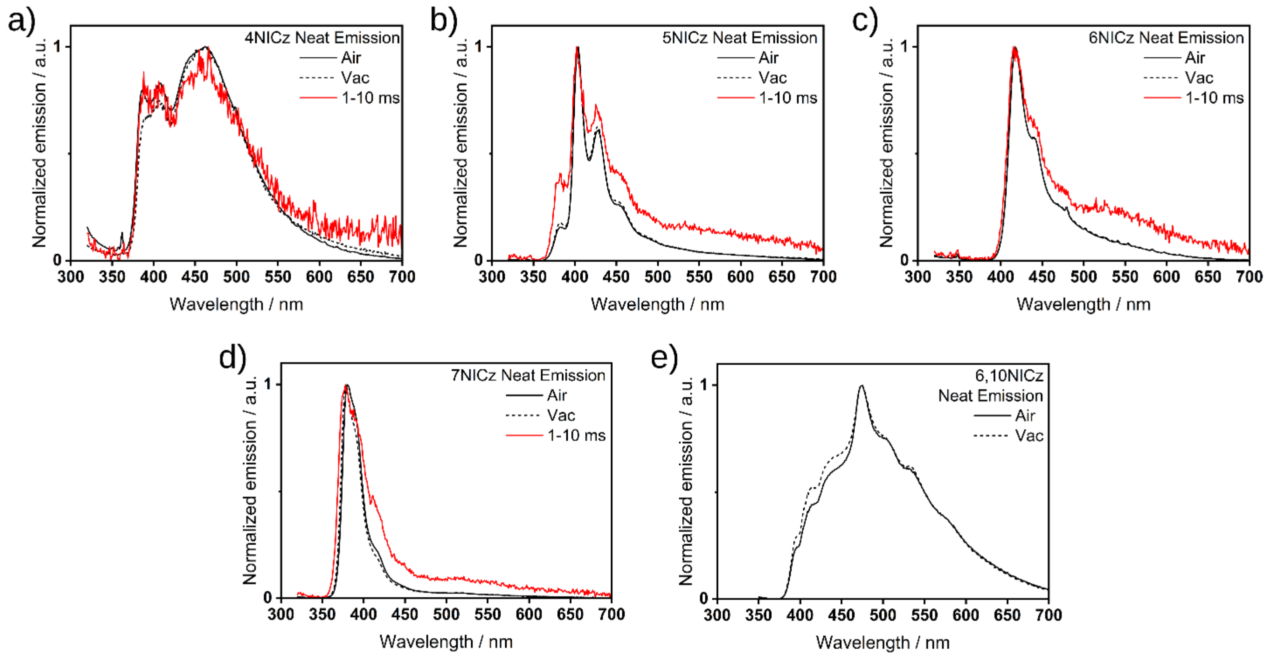

Figure **S43**. Steady state PL under air and vacuum, and 1-10 ms time-gated PL under vacuum of neat films of a) **4NICz**, b) **5NICz**, c) **6NICz**, d) **7NICz**, and e) **6,10NICz**. λ_{exc} = 295 nm (**4NICz**), 280 nm (**5NICz**), 283 nm (**6NICz**), 294 nm (**7NICz**) and 278 nm (**6,10NICz**). No time-gated PL was observed for **6,10NICz** in the neat film.

Table **S8**. Film PL of the emitters in varying concentrations.

	**0.1 wt% PMMA**		**1 wt% PMMA**				**10 wt% PMMA**		**Neat film**
**Emitter**	**$\lambda_{PL,S1}$[a] / nm**	**$\lambda_{PL,T1}$[b] / nm**	**$\lambda_{PL,S1}$[a] / nm**	**$\lambda_{PL,T1}$[b] / nm**	**$\lambda_{Phos,RT}$[c] / nm**	**$\lambda_{Phos,78K}$[d] / nm**	**$\lambda_{PL,S1}$[a] / nm**	**$\lambda_{PL,T1}$[b] / nm**	**λ_{PL} / nm (2nd peak)**
**4NICz**	370	475	371	492	474	472	385	472	462
**5NICz**	354	470	354	470	468	465	386	448	403 (427)
**6NICz**	380	473	383	473	474	473	412	475	418 (440)
**7NICz**	360	441	362	475	473	471	372	483	379
**6,10NICz**	385	486	388	487	~485	462	392	485	474

[a]Peak of the S_1 band under air. [b]Apparent peak of the T_1 band under vacuum. [c]Peak of the phosphorescence PL, as determined by gated emission over the timeframe of 1 – 10 ms, under vacuum at room temperature. [d]The same, at 77 K.

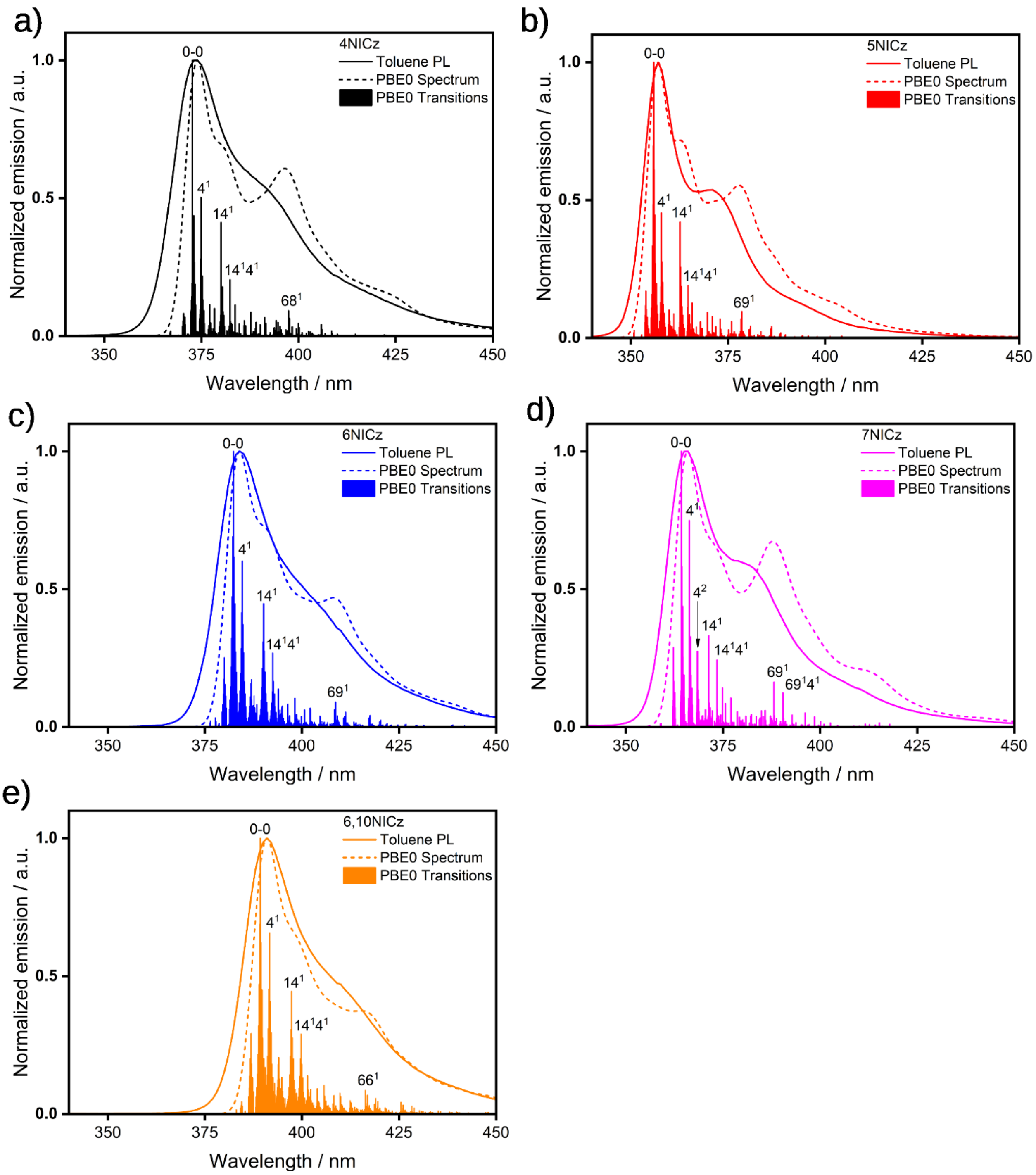

Figure **S44**. Comparison of the experimental PL spectrum in toluene and vibrationally resolved PBE0 FCHT emission spectrum from the S_1 excited state. The PBE0 calculated spectrum was simulated using Gaussian functions with full-width at half-maximum of 400 cm^{-1} (~ 0.05 eV). The PBE0 spectra and transitions were red-shifted so that the λ_{max} of both the experimental and PBE0 spectra coincide (Δ_E = 0.24, 0.25, 0.23, 0.17, and 0.24 eV, respectively). The most important transitions have been labelled x^y, where x is the vibrational mode and y the quantum number of the transition. 0-0 is the vibrational ground-state transition. The contributing vibrational modes are visualized inFigure S45

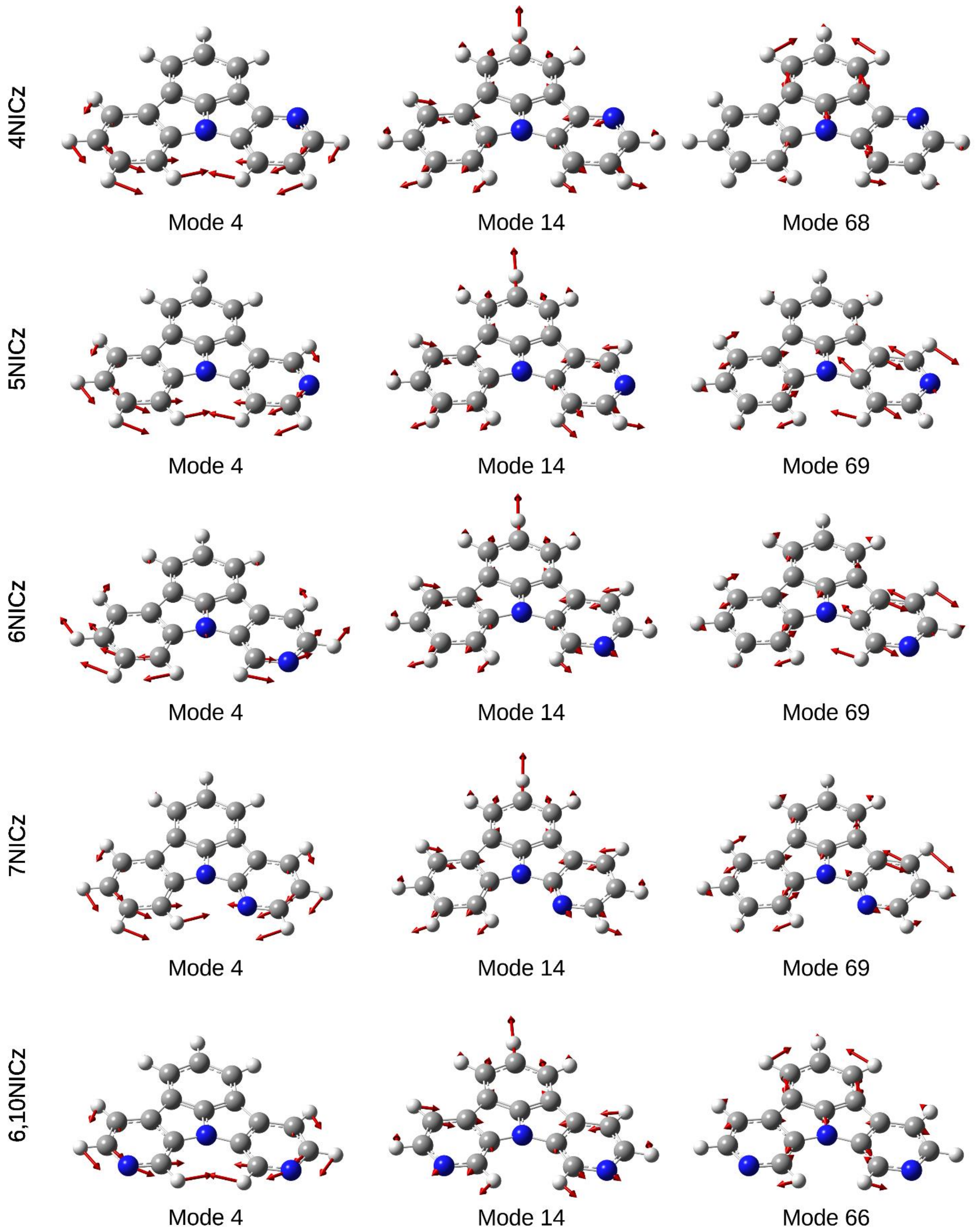

Figure **S45**. Force vectors (red arrows) of the ground-state vibrational modes of each emitter with significant contributions the simulated emission spectrum. Modes 4 and 14 are in-plane bends of the whole molecule around the central N. Modes 66/68/69 are an in-plane bond stretch over most of the molecule.

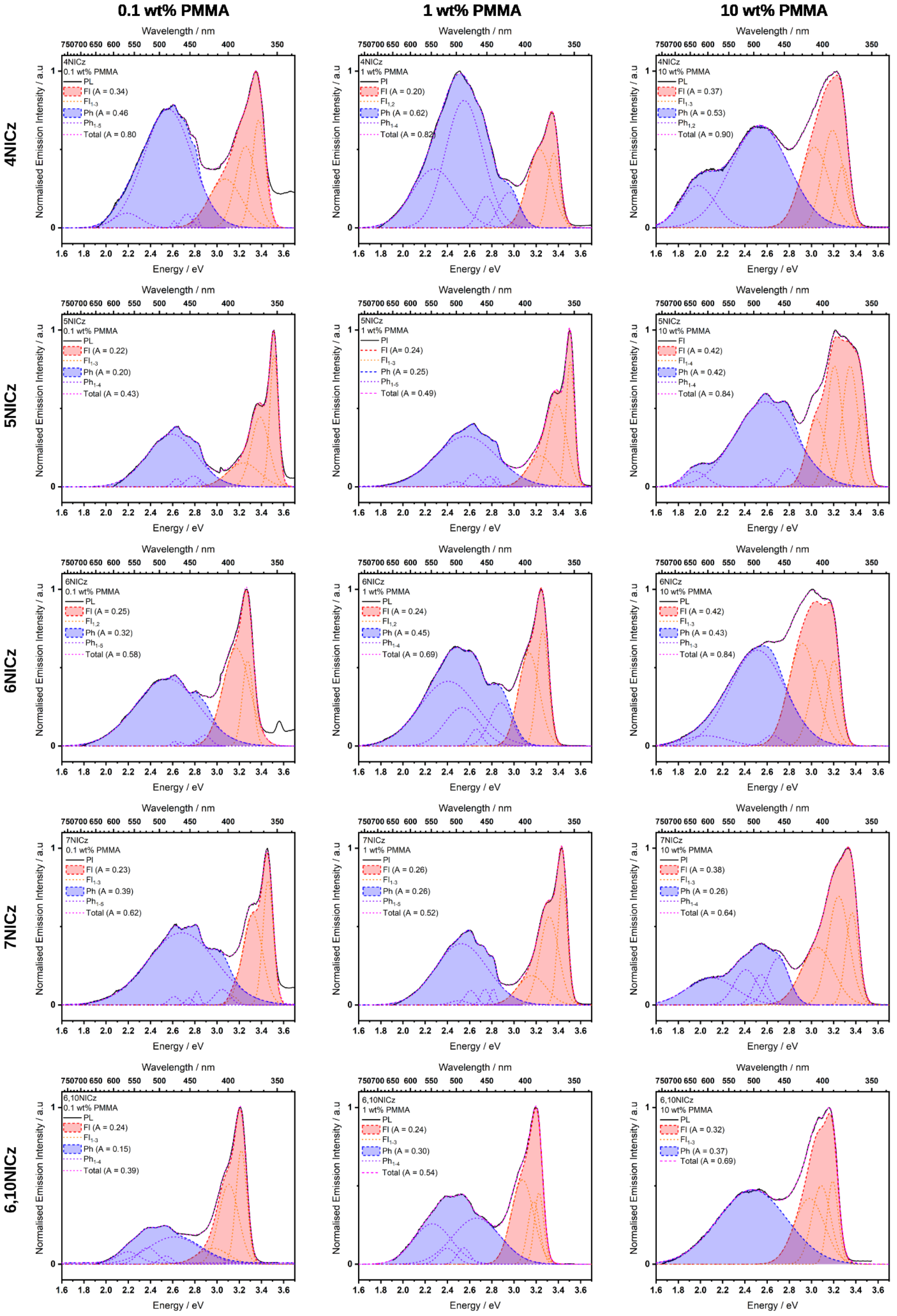

Figure **S46**. Comparison of the relative proportion of fluorescence to phosphorescence in each of

the five emitters as 0.1, 1, and 10 wt% dopants in PMMA films. The black lines show the recorded PL spectrum at room temperature under vacuum. The red and blue shaded areas are the estimated fluorescence and phosphorescence spectra respectively, determined by fitting a number of Gaussian functions to the complete PL. Each Gaussian function is shown as an orange (for fluorescence) or purple (for phosphorescence) dashed line. The total fitted spectra (the sum of all Gaussian functions) is shown as a pink dashed line. The high energy signals in the 0.1 wt% spectra are assumed to be caused by scattering from the excitation source and have not been fitted.

Table **S9**. Film PL data of the emitters at varying concentrations.

	**0.1 wt% PMMA**		**1 wt% PMMA**				**10 wt% PMMA**		**Neat film**
**Emitter**	$\lambda_{PL,S1}$[a] / nm	$\lambda_{PL,T1}$[b] / nm	$\lambda_{PL,S1}$[a] / nm	$\lambda_{PL,T1}$[b] / nm	$\lambda_{Phos,RT}$[c] / nm	$\lambda_{Phos,78K}$[d] / nm	$\lambda_{PL,S1}$[a] / nm	$\lambda_{PL,T1}$[b] / nm	λ_{PL} / nm (2nd peak)
**4NICz**	370	475	371	492	474	472	385	472	462
**5NICz**	354	470	354	470	468	465	386	448	403 (427)
**6NICz**	380	473	383	473	474	473	412	475	418 (440)
**7NICz**	360	441	362	475	473	471	372	483	379
**6,10NICz**	385	486	388	487	~485	462	392	485	474

[a]Peak of the S_1 band under air. [b]Apparent peak of the T_1 band under vacuum. [c]Peak of the phosphorescence PL, as determined by gated emission over the timeframe of 1 – 10 ms, under vacuum at room temperature. [d]The same, at 77 K.

Table **S10**. Calculated spin-orbit-coupling between S_0/T_1 and S_1/T_1.

**Emitter**	$\langle S_0 \vert H_{SO} \vert T_1 \rangle$ / cm^{-1}	$\langle S_1 \vert H_{SO} \vert T_1 \rangle$ / cm^{-1}
**4NICz**	0.005	0.759
**5NICz**	0.106	0.930
**6NICz**	0.117	0.231
**7NICz**	0.013	0.828
**6,10NICz**	0.208	0.000

Calculated using PySOC[23] from DFT calculated excited states (PBE0/6-31G**) at the DFT optimised ground state structure. As SOC is not available for ADC(2), caution should be exercised in interpreting these results.

References

[1] C. Hättig, A. Hellweg, A. Köhn, *Phys. Chem. Chem. Phys.* **2006**, *8*, 1159–1169.
[2] J. Schirmer, *Phys. Rev. A* **1982**, *26*, 2395–2416.
[3] A. B. Trofimov, J. Schirmer, *J. Phys. B At. Mol. Opt. Phys.* **1995**, *28*, 2299–2324.
[4] A. Dreuw, M. Wormit, *WIREs Comput. Mol. Sci.* **2015**, *5*, 82–95.
[5] S. Grimme, *J. Chem. Phys.* **2003**, *118*, 9095–9102.
[6] T. H. Dunning, *J. Chem. Phys.* **1989**, *90*, 1007–1023.
[7] R. A. Kendall, T. H. Dunning, R. J. Harrison, *J. Chem. Phys.* **1992**, *96*, 6796–6806.
[8] D. E. Woon, T. H. Dunning, *J. Chem. Phys.* **1993**, *98*, 1358–1371.
[9] F. Weigend, A. Köhn, C. Hättig, *J. Chem. Phys.* **2002**, *116*, 3175–3183.
[10] D. Oelkrug, H. J. Egelhaaf, J. Haiber, *Thin Solid Films* **1996**, *284–285*, 267–270.
[11] J. P. Perdew, K. Burke, M. Ernzerhof, *Phys. Rev. Lett.* **1996**, *77*, 3865–3868.
[12] J. P. Perdew, K. Burke, M. Ernzerhof, *Phys. Rev. Lett.* **1997**, *78*, 1396–1396.
[13] C. Adamo, V. Barone, *J. Chem. Phys.* **1999**, *110*, 6158–6170.
[14] S. Grimme, J. Antony, S. Ehrlich, H. Krieg, *J. Chem. Phys.* **2010**, *132*, 154104.
[15] S. Grimme, S. Ehrlich, L. Goerigk, *J. Comput. Chem.* **2011**, *32*, 1456–1465.
[16] R. Ditchfield, W. J. Hehre, J. A. Pople, *J. Chem. Phys.* **1971**, *54*, 724–728.
[17] W. J. Hehre, R. Ditchfield, J. A. Pople, *J. Chem. Phys.* **1972**, *56*, 2257–2261.
[18] P. C. Hariharan, J. A. Pople, *Theor. Chim. Acta* **1973**, *28*, 213–222.
[19] E. Runge, E. K. U. Gross, *Phys. Rev. Lett.* **1984**, *52*, 997–1000.
[20] E. K. U. Gross, W. Kohn, in *Adv. Quantum Chem.*, Elsevier, **1990**, pp. 255–291.
[21] R. Bauernschmitt, R. Ahlrichs, *Chem. Phys. Lett.* **1996**, *256*, 454–464.
[22] F. Santoro, A. Lami, R. Improta, J. Bloino, V. Barone, *J. Chem. Phys.* **2008**, *128*, 224311.
[23] X. Gao, S. Bai, D. Fazzi, T. Niehaus, M. Barbatti, W. Thiel, *J. Chem. Theory Comput.* **2017**, *13*, 515–524.
[24] O. S. Lee, M. C. Gather, E. Zysman-Colman, *Digit. Discov.* **2024**, *3*, 1695–1713.
[25] N. M. O'Boyle, A. L. Tenderholt, K. M. Langner, *J. Comput. Chem.* **2008**, *29*, 839–845.
[26] N. M. O'Boyle, M. Banck, C. A. James, C. Morley, T. Vandermeersch, G. R. Hutchison, *J. Cheminformatics* **2011**, *3*, 33.
[27] N. M. O'Boyle, C. Morley, G. R. Hutchison, *Chem. Cent. J.* **2008**, *2*, 5.
[28] W. Humphrey, A. Dalke, K. Schulten, *J. Mol. Graph.* **1996**, *14*, 33–38.
[29] J. Stone, An Efficient Library for Parallel Ray Tracing and Animation, PhD thesis, Computer Science Department, University of Missouri-Rolla, **1998**.
[30] J. D. Hunter, *Comput. Sci. Eng.* **2007**, *9*, 90–95.
[31] G. A. Crosby, J. N. Demas, *J. Phys. Chem.* **1971**, *75*, 991–1024.
[32] W. H. Melhuish, *J. Phys. Chem.* **1961**, *65*, 229–235.
[33] P. Virtanen, R. Gommers, T. E. Oliphant, M. Haberland, T. Reddy, D. Cournapeau, E. Burovski, P. Peterson, W. Weckesser, J. Bright, S. J. van der Walt, M. Brett, J. Wilson, K. Jarrod Millman, N. Mayorov, A. R. ~J. Nelson, E. Jones, R. Kern, E. Larson, C. J. Carey, İ. Polat, Y. Feng, E. W. Moore, J. Vand erPlas, D. Laxalde, J. Perktold, R. Cimrman, I. Henriksen, E. ~A. Quintero, C. R. Harris, A. M. Archibald, A. H. Ribeiro, F. Pedregosa, P. van Mulbregt, S. 1. 0 Contributors, *Nat. Methods* **2020**, *17*, 261–272.
[34] Y. Tsuchiya, S. Diesing, F. Bencheikh, Y. Wada, P. L. Dos Santos, H. Kaji, E. Zysman-Colman, I. D. W. Samuel, C. Adachi, *J. Phys. Chem. A* **2021**, *125*, 8074–8089.
[35] T. Kader, B. Stöger, J. Fröhlich, P. Kautny, *Chem. - Eur. J.* **2019**, *25*, 4412–4425.
[36] A. W. Freeman, M. Urvoy, M. E. Criswell, *J. Org. Chem.* **2005**, *70*, 5014–5019.
[37] R. J. Burns, I. K. Mati, K. B. Muchowska, C. Adam, S. L. Cockroft, *Angew. Chem. Int. Ed.* **2020**, *59*, 16717–16724.
[38] Y. Huang, H. Song, Y. Liu, Q. Wang, *Chem. – Eur. J.* **2018**, *24*, 2065–2069.

[39] S. D. Durham, B. Sierra, M. J. Gomez, J. K. Tran, M. O. Anderson, N. A. Whittington-Davis, S. Eagon, *Tetrahedron Lett.* **2017**, *58*, 2747–2750.
[40] S. Gaikwad, D. Kamble, P. Lokhande, *Tetrahedron Lett.* **2018**, *59*, 2387–2392.
[41] **2023**.
[42] G. M. Sheldrick, *Acta Crystallogr. A* **2008**, *64*, 112–122.
[43] G. M. Sheldrick, *Acta Crystallogr. Sect. Found. Adv.* **2015**, *71*, 3–8.
[44] G. M. Sheldrick, *Acta Crystallogr. Sect. C Struct. Chem.* **2015**, *71*, 3–8.
[45] A. L. Spek, *Acta Crystallogr. D Biol. Crystallogr.* **2009**, *65*, 148–155.
[46] O. V. Dolomanov, L. J. Bourhis, R. J. Gildea, J. A. K. Howard, H. Puschmann, *J. Appl. Crystallogr.* **2009**, *42*, 339–341.
